# A distinct circular DNA profile intersects with proteome changes in the genotoxic stress-related *hSOD1*^*G93A*^ model of ALS

**DOI:** 10.1186/s13578-023-01116-1

**Published:** 2023-09-13

**Authors:** Daniela Gerovska, Julie B. Noer, Yating Qin, Quratul Ain, Donjetë Januzi, Matthias Schwab, Otto W. Witte, Marcos J. Araúzo-Bravo, Alexandra Kretz

**Affiliations:** 1 Computational Biology and Systems Biomedicine, Biodonostia Health Research Institute, 20014 San Sebastian, Spain; 2 Department of Biology, Section for Ecology and Evolution, University of Copenhagen, 2100 Copenhagen, Denmark; 3 Department of Neurology, Jena University Hospital, 07747 Jena, Thuringia Germany; 4 Department of Internal Medicine IV, Hepatology, Jena University Hospital, 07747 Jena, Thuringia Germany; 5 Jena Center for Healthy Ageing, Jena University Hospital, Jena, Thuringia Germany; 6 Basque Foundation for Science, IKERBASQUE, 48013 Bilbao, Spain; 7 Max Planck Institute for Molecular Biomedicine, Computational Biology and Bioinformatics Group, 48149 Münster, North Rhine-Westphalia Germany; 8 Department of Cell Biology and Histology, Faculty of Medicine and Nursing, University of Basque Country (UPV/EHU), 48940 Leioa, Spain

**Keywords:** Amyotrophic lateral sclerosis (ALS), ALS circulome, ALS proteome, Circular DNA, Differential analysis, DNA damage and repair, eccDNA, Extrachromosomal circular DNA, Genetic heterogeneity, Neurodegeneration

## Abstract

**Background:**

Numerous genes, including *SOD1*, mutated in familial and sporadic amyotrophic lateral sclerosis (f/sALS) share a role in DNA damage and repair, emphasizing genome disintegration in ALS. One possible outcome of chromosomal instability and repair processes is extrachromosomal circular DNA (eccDNA) formation. Therefore, eccDNA might accumulate in f/sALS with yet unknown function.

**Methods:**

We combined rolling circle amplification with linear DNA digestion to purify eccDNA from the cervical spinal cord of 9 co-isogenic symptomatic *hSOD1*^*G93A*^ mutants and 10 controls, followed by deep short-read sequencing. We mapped the eccDNAs and performed differential analysis based on the split read signal of the eccDNAs, referred as DifCir, between the ALS and control specimens, to find differentially produced per gene circles (DPpGC) in the two groups. Compared were eccDNA abundances, length distributions and genic profiles. We further assessed proteome alterations in ALS by mass spectrometry, and matched the DPpGCs with differentially expressed proteins (DEPs) in ALS. Additionally, we aligned the ALS-specific DPpGCs to ALS risk gene databases.

**Results:**

We found a six-fold enrichment in the number of unique eccDNAs in the genotoxic ALS-model relative to controls. We uncovered a distinct genic circulome profile characterized by 225 up-DPpGCs, *i.e.*, genes that produced more eccDNAs from distinct gene sequences in ALS than under control conditions. The inter-sample recurrence rate was at least 89% for the top 6 up-DPpGCs. ALS proteome analyses revealed 42 corresponding DEPs, of which 19 underlying genes were itemized for an ALS risk in GWAS databases. The up-DPpGCs and their DEP tandems mainly impart neuron-specific functions, and gene set enrichment analyses indicated an overrepresentation of the adenylate cyclase modulating G protein pathway.

**Conclusions:**

We prove, for the first time, a significant enrichment of eccDNA in the ALS-affected spinal cord. Our triple circulome, proteome and genome approach provide indication for a potential importance of certain eccDNAs in ALS neurodegeneration and a yet unconsidered role as ALS biomarkers. The related functional pathways might open up new targets for therapeutic intervention.

**Graphical Abstract:**

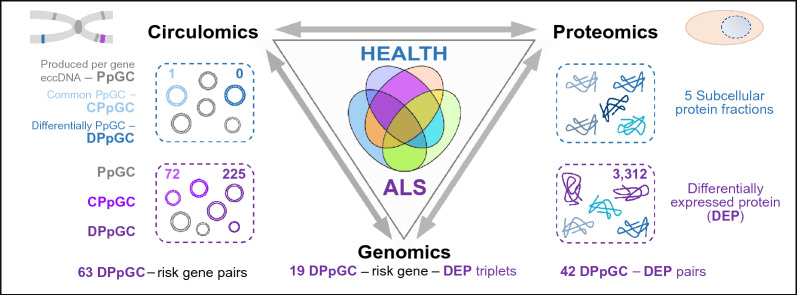

**Supplementary Information:**

The online version contains supplementary material available at 10.1186/s13578-023-01116-1.

## Background

Loss of DNA integrity is a hallmark of aging [1] and pivotal in the pathophysiology of age-related neurodegenerative disorders, including amyotrophic lateral sclerosis (ALS). Threat to DNA integrity in ALS is crucially linked to inherited or somatic alterations in genes that occupy a primary or additional role in genome protection, DNA damage response (DDR) signaling, and DNA repair. Among them are the *C9ORF72* [2–4], *SOD1* [5–8], *TDP-43* [9–11] and *FUS* [12–15] genes accounting for ~ 70% of familial ALS (fALS) and ~ 12% of sporadic ALS (sALS) cases. The same applies to variants in susceptibility or modifier *loci* of lower frequency such as *C21ORF2* [16], *NEK1* [17] and *SETX* [18], among others.

Mutations in the *SOD1* gene are responsible for ~ 20% of the fALS and a percentage of sALS cases and thus represent the second most common genetic source of human ALS [for review, see 19]. The encoded Cu, Zn-superoxide dismutase 1 (SOD1) enzyme plays a crucial role in genome protection against endogenous and environmental reactive oxygen species (ROS). First, it reduces ROS by catalyzing superoxide anion radicals into O_2_ and H_2_O_2_ [20]. Second, it participates in the DDR [21] and activates the promotors of genes involved in ROS-resistance and DNA damage repair [5]. Accordingly, pathogenic *SOD1* loss- and gain-of-function mutations are reasoned to augment oxidative genomic damage partly through impaired dismutase function and primarily through increased hydroxyl radical production [22–25]. In support, markers of oxidative genomic damage are elevated in body fluids of ALS patients carrying *SOD1* mutations [26, 27] and in spinal cord tissue of *SOD1*^*G93A*^ mouse mutants [28, 29], while transfection with WT *hSOD1* reduces the level of ROS and DNA damage after exposure to H_2_O_2_ [7, 23]. Apart from causing oxidative genomic damage, ROS accumulation propagates R-loops, triple-stranded DNA:RNA hybrids formed at sites of gene transcription, which can provoke double strand breaks (DSB) and genomic instability if their resolution is hampered and induce specific DNA repair pathways [30, 31]. Particularly at repeat structures, double-R-loop configurations involving bidirectional transcription are prone for improper realignment of the DNA strand to the complementary strand and the activation of error-prone repair mechanisms [32].

Apart from these ALS-related genetic factors impacting genome integrity and repair, the central nervous system (CNS) is endogenously prone to accumulate DNA damage: Postmitotic neurons are severely restricted in error-free DNA repair cascades [33] and lack homologous recombination (HR). In parallel, neurons face persistent exposure to endogenous ROS due to their high oxygen consumption [34], conveying the risk for single strand breaks (SSB) and DSB through nearby strand oxidations. Moreover, the high transcriptional activity in neurons predisposes for strand injuries, *e.g.*, by implicating breaks and nicks from R-loop processing and cleavage [30]. Finally, neurons might accumulate unrepaired genomic injuries as they remain lifelong unreplaced. However, how DNA damage influences ALS is still not fully understood.

Outstanding among the pathophysiological consequences involved in multi-order sequelae of genomic instability, DDR and DSB repair is the formation of extrachromosomal circular DNA (eccDNA), a by-product of chromothripsis and chromoplexy (*i.e.*, shattering and *de novo* assembly of one or more chromosomes), DNA deletions, inversions, recombination events, breakage-fusion-bridge (BFB) cycles and likely BFB-related copy number amplifications [35–39], all events of which can result directly from DSB or from their repair-related resection and excision processes [38, 40]. Accordingly, eccDNAs exist in sizes ranging from a few hundred bp (micro-DNA) to kilo bp (small polydispersed circular DNA; spcDNA) or several mega bp (ecDNA; double minutes), which form from all parts of the human genome [37] and have been detected in all tissues examined so far, including brain tissue [38]. Circular DNA can amplify pathogenicity genes in conjunction with their enhancers on larger circles [36, 41, 42] and boost transcription through higher copy numbers, rearrangement of genomic segments, new enhancer topologies, improved chromatin accessibility on the circles, and spatial clustering of multiple ecDNAs [36, 41, 43, 44]. Accordingly, the presence of ecDNA is a negative prognostic factor in tumors [36, 45]. Besides large ecDNAs, healthy cells and tumor cells also produce small eccDNAs [37, 38] of yet poorly defined function. Small eccDNAs can interfere with gene expression from the linear genome, *e.g.*, through promoter-independent transcription into small regulatory miRNAs or the generation of exon-derived si-like RNAs [46]. Similar to ecDNA, they might operate as mobile vehicles whose docking and interaction with chromatin modifies transcription throughout the genome [47]. Moreover, as circles can be chimeric for different *loci* and integrate back into the linear genome [43], they contribute to genetic heterogeneity. Irrespective of size, episomal circular DNA has also been reported to elicit pro-inflammatory immune cascades that aggravate the course of many neurodegenerative disorders [48–51]. Notably, DNA damage itself recruits key factors of the related inflammatory cascades to nuclear sites of DSB where they inhibit DNA repair pathways [52] and hence such mutual interaction might further sustain eccDNA production.

Despite expeditious progress in eccDNA research, the role of eccDNA in non-tumor CNS disorders and in the rising proportion of neurodegenerative entities implicating hereditary genomic instability is unknown. According to the knowledge delineated, we inferred that ALS neurodegeneration increases the levels of circular DNA. To test this hypothesis, we screened for eccDNA in the spinal cord of a murine *hSOD1*^*G93A*^ genotoxic stress model of ALS and contrasted the eccDNA profiles with wild type conditions. To understand whether the circulome specifically released in symptomatic animals correlates with the ALS pathophysiology, we compared the catalog of genes up-producing eccDNA in ALS with protein products changed in the ALS proteome and with pre-established genomic ALS risk and susceptibility *loci*.

## Methods

### Animals

The B6.Cg-Tg(SOD1*G93A)1Gur/J strain (RRID: IMSR_JAX:004435) served as *Mus musculus* animal model [53] to study ALS under *SOD1*^*G93A*^ mutational conditions that provoke f/sALS in humans [54]. Motor symptoms in male mutants developed at ages between 18 and 21 weeks, and were assessed according to a standardized, investigator-neutral clinical score protocol. Inclusion required the manifestation of a defined severity score within the target age indicated. Sex- and age-matched C57BL/6J mice served as controls. Animals that did not timely develop a motor phenotype were excluded from the study.

### Tissue extraction

After score approval, animals were euthanized by an overdose of volatile isoflurane CP^®^ (CP Pharma, Burgdorf, Germany). The spine was microscopically dissected from surrounding tissue, the vertebral column was opened and the myelon was exposed. The spinal cord was excavated *in toto*, detached from meninges and transferred to ice-cold PBS. The cervical segment was dissected from thoracic and lumbar parts, collected into Eppendorf tubes and immediately frozen at − 80 ℃ for further analysis.

### DNA isolation

DNA extraction from cervical spinal cord was identically performed for control and mutant samples, following kit-facilitated standard procedures (Nucleospin tissue DNA/RNA and Protein kit; Macherhey Nagel GmbH & Co KG, Germany). Briefly, each tissue specimen was thawed and homogenized with a motorized pestle applying 25 µl of proteinase k to the initial buffer system. Each suspension was vortexed vigorously and the homogenate was kept at 56 ℃ overnight. The next day, the samples were vortexed, supplemented by the indicated buffer solution and incubated at 70 ℃ for 10 min. For DNA extraction, 210 µl of ethanol were added to each sample suspension. Solutions were poured on the nucleospin columns, placed into the collection tubes and centrifuged at 11,000 × g for 1 min. The flow-through was discarded and the columns were again centrifuged as aforementioned. The extracted DNA was washed in 500 µl of appropriate buffer solution and centrifuged. The flow-through was discarded and the column was centrifuged as before. The washing step was repeated with an adequate buffer solution. The flow-through was discarded and the column was centrifuged as before to dry the silica membrane, then transferred to a sterile 1.5 ml Eppendorf tube. The collected genomic DNA was eluted in 30 µl of appropriate pre-warmed buffer and incubated at RT for 2 min, then centrifuged at 11,000 × *g* for 2 min. The genomic DNA concentration of each sample was measured spectrophotometrically by NanoDrop systems.

### EccDNA purification

Total DNA extracted from mouse cervical spinal cord was digested with exonuclease V (M0345, New England Biolabs, Ipswich, MA, USA) to remove linear DNA. 50,000 copies of the plasmid p4339 (gift from Charlie Boone, University of Toronto), 20,000 copies of YGPM25009 (gift from Michael Lisby, University of Copenhagen, UCH; backbone derived from pGP564, Open Biosystems, Huntsville, AL, USA) and 10,000 copies of pBR322 (New England Biolabs) were added as internal controls to 400 ng of DNA per sample. These DNA mixtures were incubated at 37 ℃ with 30 IU exonuclease V (RecBCD), 1 × NEBuffer 4 and 1 mM ATP. Every 24 h, 30 IU fresh exonuclease V and 1 mM ATP were added to the reactions, which were incubated for 7 days in total. Thereafter, exonuclease reactions were inactivated for 30 min at 70 ℃ and subjected to DNA clean-up with Agencourt AMPure XP beads (A63881, BD, Franklin Lakes, NJ, USA) according to the manufacturer’s instructions. Briefly, reactions were mixed with 1.8 × beads solution and incubated at RT for 5 min to bind. Supernatants were discarded, beads were washed twice with 80% ethanol and briefly evaporated. DNA was eluted in 60 µl of 10 mM Tris–HCl, pH 8.0. Removal of linear DNA was confirmed by PCR analysis on the presence and absence of the *Cox5b* sequence in DNA before and after treatment, respectively. The numbers of unique eccDNAs were normalized to the total DNA amount while the enrichment of plasmid *pBR322* DNA relative to single control *Cox5b* gene concentration was not assessed. 15 µl of exonuclease-digested DNA were subjected to rolling circle amplification (RCA) using TruePrime RCA (370025, 4BaseBio, Trueprime Whole Genome Amplification kit) according to manufacturer’s instructions. Briefly, DNA was denatured for 3 min with buffer D and neutralized with buffer N. Denatured DNA was mixed with enzyme 1, enzyme 2, dNTPs and reaction buffer and incubated at 30 ℃ for 48 h. Afterwards, reactions were inactivated for 10 min at 65 ℃.

### Short-read sequencing

After DNA extraction from control and ALS samples, the φ29-polymerase amplified DNA strands were sheared into fragments of 200 bp and ~ 350–400 bp in size using a Covaris LE220 ultrasonicator (Covaris, Brighton, UK) with operating parameters set as the following: 20 s ultrasound, acoustic duty factor 25%, peak incident power 500 W, cycles per burst 500, 24 cycles. Appropriate fragment size (200–500 bp) was captured with the aid of AMPure XP beads (Agencourt, Beverly, USA). Fragmented DNA was then incubated with bacteriophage T4 DNA polymerase (ENZYMATICS, Beverly, USA) for 30 min at 20 ℃ for trimming to obtain blunt ends devoid of 3′-overhangs or 5′-gaps, which were further 3′-adenlyated to create sticky ends. These DNA fragments were ligated at both ends to T-tailed adapters and amplified via PCR according to the following step-wise procedures: 3 min at 95 ℃ followed by 8 cycles of 20 s at 98 ℃; 15 s at 60 ℃; 30 s at 72 ℃; 10 min at 72 ℃ for further elongation. The obtained PCR products were purified via AMPure XP beads (Agencourt, Beverly). The DNA library was sequenced on BGISEQ-500 by paired-end sequencing of 150 bp intervals both from the forward and reverse strand, with an average sequencing data of 138 M reads per library, to provide high-quality alignment across all genomic regions.

### Mapping of eccDNA and quantification of produced per gene eccDNAs (PpGCs)

Mapping and quantification of produced per gene eccDNAs (PpGCs) was realized as described [55, 56]. EccDNA reads were mapped with Circle_Finder [38, 57] using as arguments the mm10 built of the mouse genome and a minNonOverlap threshold between two split reads of 10 bp. Clusters of eccDNAs within a distance smaller than *D*_*min*_ = 10 bp were coalesced and the number of the split reads detecting them were summed up as split reads of the merged eccDNAs. EccDNAs with less than two split reads were excluded. EccDNA annotations were accomplished with bedtools intersect [58] using Genecode gene coordinates. EccDNA sizes were assessed from mt-free DNAs, setting 100 Kbp as cutoff for all short circles. Next, the number of split reads of all those eccDNAs that carried the same gene or fragment of a gene were added up to obtain the unscaled produced per gene eccDNA (PpGC_*i*_) sum for each gene *i*. For scaling by gene length, each PpGC_*i*_ was multiplied by a scale factor *L*_*Max*_/*L*_*i*_, where *L*_*Max*_ is the length of the longest gene found in the dataset, and *L*_*i*_ is the length of the gene *i*. Finally, data equalization was performed by the log_2_(PpGC + 1) transformation of the quantified PpGCs to obtain the final PpGCs.

### Identification of differentially produced per gene eccDNAs (DPpGCs)

We used our method, DifCir, for differential analysis of sequenced purified eccDNA data based on their split read signal [55, 56]. We calculated the mean values of the PpGCs for each group of replicates and selected for differentially produced per gene eccDNAs (DPpGCs), whose absolute difference of the means between the two groups was more than a selection threshold θ_DPpGC_ = onefold change (FC) in log_2_ scale. Finally, we selected the statistically significant DPpGCs using a significance threshold of α_DPpGC_ = 0.01.

### Democratic method for finding common PpGCs (CPpGCs)

Similar to the procedure previously reported in transcriptomics [59], DNA methylomics [60] and circulomics [55, 56], we implemented a democratic method to search for commonly abundant molecules in different samples. To find the minimum cutoff value of PpGC to be considered as a positive vote, we calculated the empirical PpGC distribution for controls and ALS. As threshold, we chose the ceiling of the PpGC (ceil(7.0476)) that corresponded to the maximum of the distribution and which numerically equaled to 8. For the ALS group, the corresponding ceil threshold was (ceil(8.00402)) that equated to 9. Finally, we selected the genes that showed at least 3 votes for both control and ALS samples.

### Extrachromosomal telomere repeats (ECTR) assessment

We estimated the total length of extrachromosomal telomere repeat length (ECTR) in Kbp on the eccDNA circles for each sample adapting the TelSeq software to work on mouse circular DNA-seq data [61].

### Protein isolation from cervical myelon and subcellular fractionation

Ventral cervical spinal cord comprising the motor neuron population was dissected from the dorsal moiety, homogenized via Ultra-Turrax (IKA Labortechnik, Germany) in ice-cold lysis buffer supplemented with a protease inhibitor cocktail (Sigma-Aldrich, Germany) and subjected to sonication (BANDELIN electronic, Germany) or mechanical straining through a syringe (Ø 400 μm). Subcellular protein fractionation was performed as for cortical brain tissue [62], with adaptions for spinal cord, based on a ‘subcellular protein fractionation kit for tissue’ (Thermo Fisher Scientific). Briefly, 50 mg (w/w) of freshly isolated tissue were washed in ice-cold PBS, cut into pieces and added to CE-B buffer. The suspension was homogenized, strained and centrifuged at 500 × *g* for 5 min at 4 ℃. The supernatant was separated as cytoplasmic ‘CP’ fraction. The pellet was reconstituted in ME-B buffer, incubated for 10 min at 4 ℃ on a shaker and centrifuged at 3000 × *g* for 5 min. The supernatant was separated as membrane ‘ME’ fraction. The pellet was reconstituted in NE-B buffer and incubated for 30 min on a shaker, then centrifuged at 5000 × *g* for 5 min. The supernatant was separated as soluble nuclear ‘sNE’ fraction. The NE-B buffer was supplemented with CaCl_2_ and MNase at RT and added to the pellet. After 30 min of incubation, the suspension was centrifuged at 16,000 × *g*. The supernatant was separated as chromatin-bound ‘cNE’ nuclear fraction. The pellet was mixed with PE-B buffer and incubated for 10 min at RT, followed by centrifugation at 16,000 × *g* for 5 min. The supernatant was collected as cytoskeletal ‘Cysk’ fraction. All buffers were supplemented with HALT protease inhibitors (1:100). CE-B and ME-B were applied as ice-cold solutions, NE-B and PE-B at RF. Protein concentrations were assessed with the Bradford Assay using the QuickStart™ Bradford Dye Reagent (Bio-Rad, Hercules, CA, USA). To validate purity, 10 μg of each subfraction were examined by a standard SDS-PAGE-based western blot protocol, applying primary antibodies against GAPDH (rabbit α-GAPDH, clone 14C10, 1:8000, Cell Signaling Technology Cat# 2118, RRID: AB_561053) or Histone H3 (α-Histone H3, 1:10,000, Abcam Cat# ab1791, RRID: AB_302613), and further approved by MS-based marker protein enrichments (unpublished data).

### Mass spectrometry

Sub-cellular fractions corresponding to approximately 30 μg of protein extract were processed as in Buczak et al. [63]. Briefly, proteins were solubilized by addition of SDS to a final concentration of 2% (v/v), followed by sonication in Bioruptor Plus (Diagenode) and heating for 10 min at 95 ℃. Following reduction and alkylation, proteins were precipitated by cold acetone precipitation. The resulting pellets were resuspended in digestion buffer (3 M Urea in 100 mM HEPES, pH 8.0) and digested by consecutive addition of LysC (Wako, 3 h at 37 ℃) and trypsin (Promega, 16 h at 37 ℃). The obtained digested peptides were acidified and desalted with a Waters Oasis^®^ HLB µElution Plate 30 µm (Waters) following the manufacturer’s instructions. The desalted peptides were dissolved in 5% (v/v) acetonitrile, 0.1% (v/v) formic acid to a peptide concentration of approximately 1 μg/μl and spiked with iRT peptides (Biognosys AG) prior to analysis by LC–MS/MS. For spectral library generation, a pool of approximately 1 μg of reconstituted peptides for each fraction was analyzed using Data Dependent Acquisition (DDA) using the nanoAcquity UPLC system (Waters) fitted with a trapping (nanoAcquity Symmetry C18, 5 μm, 180 μm × 20 mm, Waters) and an analytical column (nanoAcquity BEH C18, 2.5 μm, 75 μm × 250 mm, Waters). The outlet of the analytical column was coupled directly to an Orbitrap Fusion Lumos (Thermo Fisher) using the Proxeon nanospray source. The samples were loaded with a constant flow of solvent A. Peptides were eluted via a non-linear gradient from 0 to 40% solution B in 120 min. The peptides were introduced into the mass spectrometer via a Pico-Tip Emitter 360 μm OD × 20 μm ID; 10 μm tip (FS360-20-10-D-20, New Objective). Total run time was 145 min, including clean-up and column re-equilibration. The RF lens was set to 30%. The conditions for DDA data acquisition were as follows: Full scan MS spectra with mass range 350–1650 m/z were acquired in profile mode in the Orbitrap with resolution of 60,000 FWHM. The filling time was set at maximum of 50 ms with limitation of 2 × 10^5^ ions. The “Top Speed” method was employed to take the maximum number of precursor ions (with an intensity threshold of 5 × 10^4^) from the full scan MS for fragmentation (using HCD collision energy, 30%) and quadrupole isolation (1.4 Da window) and measurement in the Orbitrap (resolution 15,000 FWHM, fixed first mass 120 m/z), with a cycle time of 3 s. The MIPS (monoisotopic precursor selection) peptide algorithm was employed but with relaxed restrictions when too few precursors meeting the criteria were found. The fragmentation was performed after accumulation of 2 × 10^5^ ions or after filling time of 22 ms for each precursor ion (whichever occurred first). MS/MS data were acquired in centroid mode. Only multiply charged (2+ to 7+) precursor ions were selected for MS/MS. Dynamic exclusion was employed with maximum retention period of 15 s and relative mass window of 10 ppm. Isotopes were excluded. For the DIA data acquisition, approx. 1 μg of reconstituted peptides for each sample were loaded and the same gradient conditions were applied to the LC as for the DDA. The MS conditions were varied as follows: Full scan MS spectra with mass range 350–1650 m/z were acquired in profile mode in the Orbitrap with resolution of 120,000 FWHM. The filling time was set at maximum of 20 ms with limitation of 5 × 10^5^ ions. DIA scans were acquired with 34 mass window segments of differing widths across the MS1 mass range with a cycle time of 3 s. HCD fragmentation (30% collision energy) was applied, and MS/MS spectra were acquired in the Orbitrap at a resolution of 30,000 FWHM over the mass range 200–2000 m/z after accumulation of 2 × 10^5^ ions or after filling time of 70 ms (whichever occurred first). Ions were injected for all available parallelizable time. Data were acquired in profile mode. For data acquisition and processing of the raw data Xcalibur v4.0, Tune v2.1 (Thermo Fisher) were used. Acquired data were processed using Spectronaut Professional v11 (Biognosys AG). Raw files (DDA and DIA) were searched against the mouse UniProt database (*Mus musculus*, entry only, release 2016_01) with a list of common contaminants appended using Pulsar (Biognosys AG) with default settings. The library contained 112,444 precursors corresponding to 5687 protein groups based on Spectronaut protein inference. For quantification, raw DIA files were searched against the project-specific library using default BGS factory settings, except: Major Group Quantity = Median peptide quantity; Minor Group Quantity = Median precursor quantity; Minor Group Top N = OFF; Normalization Strategy = Local normalization; Row Selection = Qvalue sparse.

### Immunofluorescence

Mice were exposed to an overdose of vaporized Isoflurane CP^®^. Transcardial perfusion was initiated at onset of cardiorespiratory arrest by applying a 4% formaldehyde solution in PBS (pH 7.3). The entire spinal cord axis was exposed, the cervical segment was dissected, cryoprotected in sucrose and frozen in 2-methylbutane. Serial axial cryo-sections were sliced at 25 µm (Leica Biosystems, Germany). Immunofluorescent staining was preceded by antigen retrieval with 0.5% saponine. To prevent non-specific epitope binding, slices were pre-incubated in 10% NGS or NDS in 3% BSA/PBS containing 0.3% Triton X-100 for at least 2 h at RT. Primary antibodies (mouse α-NFH, clone SMI-32, 1:500; Biolegend Cat# 801,701, RRID: AB_2564642; rabbit α-mH2A1, 1:600; Abcam Cat# ab183041; RRID: AB_2716576) were diluted in 2% NDS or NGS in 3% BSA/PBS containing 0.3% Triton X-100 and incubated at 4 ℃ for 48 h (α-mH2A1) or overnight (α-NFH). After washing trice in PBS, the slices were reacted with an appropriate secondary antibody solution containing 10% NGDS or NGS in 3% BSA/PBS supplemented with 0.3% Triton X-100, followed by further washing steps. Cell nuclei were counterstained with 2 µg/ml 4,6-diamidino-2-phenylindole dihydrochloride (DAPI) in PBS for 5 min and washed trice. For microscopy, samples were mounted with Fluoromount-G^®^ (SouthernBiotech, USA).

Specimens were imaged as z-stacks using confocal laser scanning microscopy (LSM 710 or LSM 900; Zeiss, Germany) applying 40 × and 63 × immersion oil objectives and processed with the ZEN 3.0 software (Zeiss, Germany). For quantitative analyses, 8–10 serial ventral horn slices per specimen were systematically screened. n = 3 in the control and ALS group.

### Statistical analyses

Statistical significance for the comparison of unique eccDNA frequencies between control and ALS samples were calculated according to the non-parametric Wilcoxon rank sum test (*n* = 10 for controls, *n* = 9 for ALS samples). For the assessment of chromosomal *loci* enriched in genes that generated up-DPpGCs, a hypergeometric test was used. DPpGC analyses were performed based on Student’s *t*-test.

Differential abundance testing of proteins was performed in Spectronaut using a paired *t*-test between replicates. *P*-values were corrected for multiple testing with the method described by Storey [64]. Protein groups were considered as significantly affected if they displayed a *q*-value < 0.05 and an absolute average log_2_ ratio > 0.38. For histological analyses, *p*-values < 0.05 assessed by two-way ANOVA were considered as statistically significant.

## Results

### Genotoxic stress in SOD1^G93A^ mutants

Oxidative genomic damage is increased in spinal cord tissue of *SOD1*^*G93A*^ mouse mutants as approved by OdG and γH2AX markers [26–29]. Recent studies indicate that the macrodomain-containing core histone variant macroH2A1 (mH2A1) is involved in DDR and DNA damage repair following its attraction to DSB [65]. Under oxidative stress conditions, it stabilizes the DDR sensor poly(ADP)ribose PAR, reduces oxidative DNA damage and prevents cell death [66]. According to this role, we observed an increased number of nuclear mH2A1^+^ *foci* in *hSOD1*^*G93A*^ mutant SMI-32^+^ motor neurons (MN) relative to control MN as quantified from grey matter areas (Additional file 1: Fig. S1A–C). In contrast to other histone variants, mH2A1 is transcribed throughout the cell cycle and not limited to S phase. To further prove this, mH2A1 detection in post-replicative MN was assessed under co-expression of a cell cycle-sensitive Fucci (fluorescent ubiquitination-based cell cycle indicator) reporter [67] thus confirming a G_0_/G_1_ state (Additional file 1: Fig. S1A, B). These observations complement previous studies on genotoxic stress evaluation in the *SOD1*^*G93A*^ strain [26–29].

### Circulomics and proteomics workflow

**Fig. 1:**
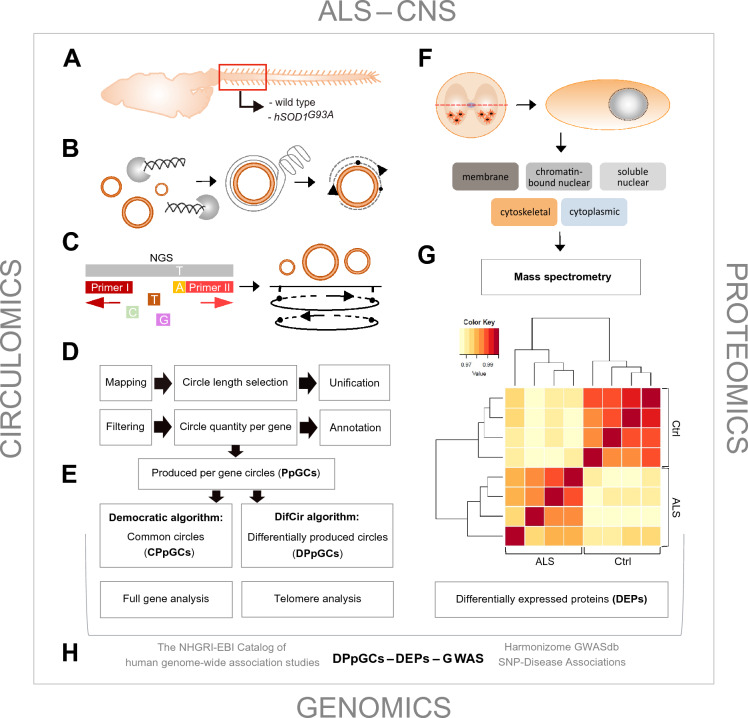
Experimental setup and workflow for circulomics and proteomics profiling in spinal cord. **A** Total DNA was purified from cervical myelon of control and symptomatic *hSOD1*^*G93A*^ mutant animals. **B** Circular DNA purged from linear DNA was amplified and assembled. **C**. EccDNA was subjected to short-read sequencing and mapped to genomic coordinates. **D**–**E** Computational quantitative algorithms were generated that in silico reconstructed circle sizes, determined the originating genomic coordinates and profiled them as being common or different in each group. Moreover, algorithms were designed to find full gene and telomere-specific circles. Filter means excluded mitochondrial circular DNA. **F**–**G** Mass spectrometry-based proteomic studies were performed on cervical spinal cord in a cell compartment-specific manner, dissecting 5 subcellular fractions. Changes in protein abundances between ALS and control specimens, as exemplified in the heatmap for the cytoplasmic (CP) fraction, were selected for eccDNA-related gene products. **H** Output genes from eccDNA and proteome analyses were referenced to GWAS. For eccDNA analyses, *n* = 9 for ALS and *n* = 10 for control samples. For MS studies, *n* = 5 for both groups. ALS, *hSOD1*^*G93A*^ mutant samples; Ctrl, control samples

Under corresponding conditions, eccDNA was isolated from cervical spinal cord of 9 symptomatic *hSOD1*^*G93A*^ mutant mice and 10 controls. In short, for each individual sample eccDNA was enriched from 400 ng total genomic DNA by removal of linear DNA with exonuclease, followed by RCA. The obtained eccDNA pool was normalized against each sample’s total DNA amount, followed by paired-end short read sequencing as described earlier [37]. Mapping of the purified circles discovered hundreds to thousands of eccDNAs in the control and disease-affected *Mus musculus* cervical spinal cord, from which the gene-specific eccDNAs, PpGCs, were calculated. DifCir-based differential analysis [55, 56] of the genic eccDNAs identified a set of PpGCs that were differentially produced in ALS, DPpGCs, and that were up-produced under disease-like conditions (up-DPpGCs) compared to controls. Applying the democratic method [56, 59, 60] for finding common PpGCs (CPpGCs) expanded the set of eccDNA gene hotspots. The experimental setup and computational workflow for eccDNA mapping and differential analysis, and subsequent proteomics analysis, is presented in Fig. 1.

### Unique eccDNAs are more abundant in the ALS diseased than control CNS

**Fig. 2:**
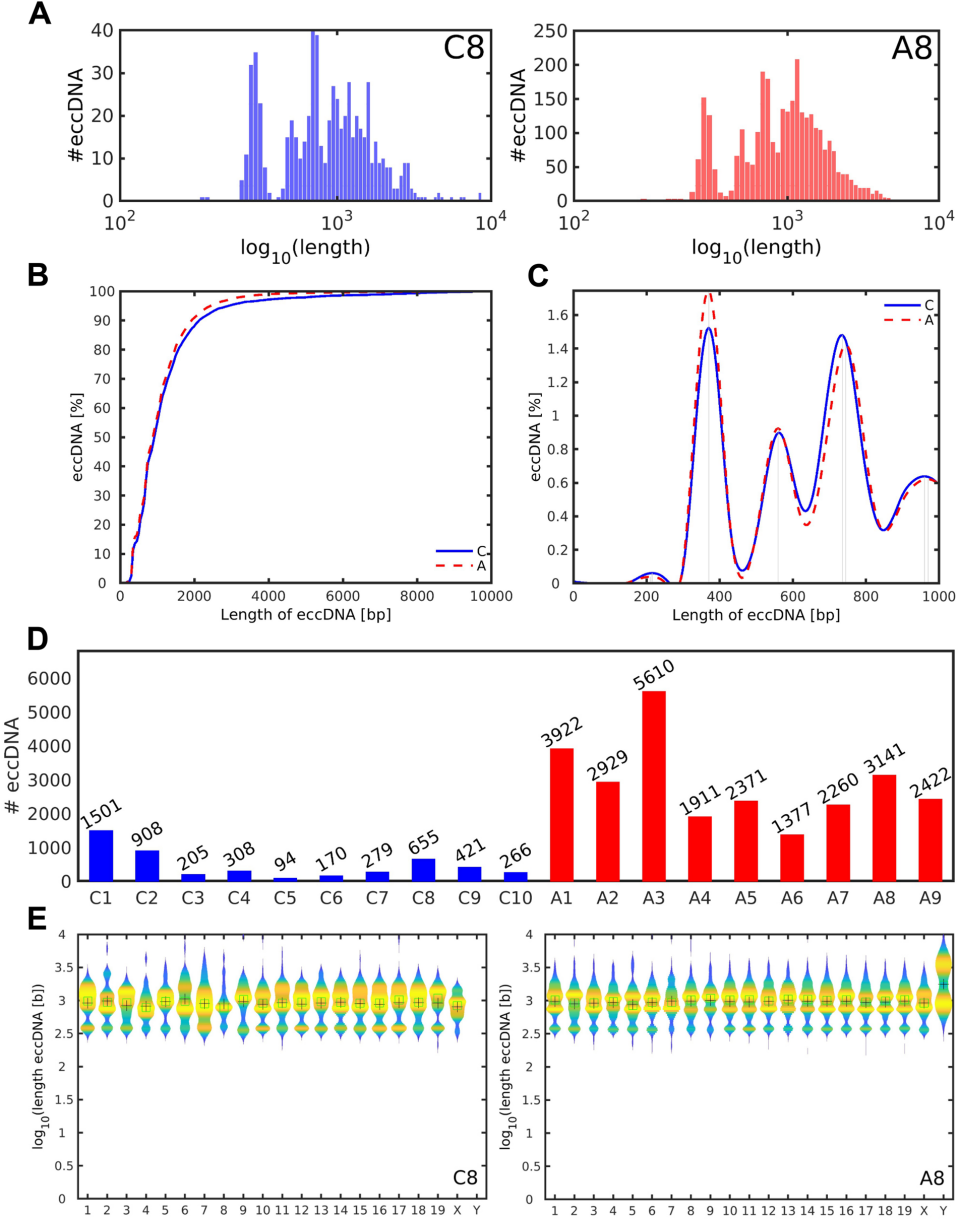
Length-adjusted frequencies of unique eccDNAs. **A** Exemplified for a control (C8; blue) and ALS (A8; red) sample are the length-determined frequencies of unique eccDNA species, as assessed across the entire murine chromosomal set up to a size of 10^4^ bp. **B** Plateau of eccDNA frequencies (in %) between 10^3^ and 10^4^ bp. **C** Periodic enrichment of eccDNAs up to a size of 10^3^ bp in both groups. Vertical lines mark the local peak maxima after smoothing. **D** Bar plot indicating cumulative numbers of unique eccDNAs for each control (C1-C10 in blue) and ALS (A1-A9 in red) sample up to a size threshold of 10^5^ bp. **E** Violin plots of the length distributions of eccDNAs (< 10^5^ bp) across all murine chromosomes as exemplified for a control (C8) and ALS (A8) sample. Mean and median eccDNA read lengths derived from each chromosome are displayed as red crosses and green squares, respectively. Data points are overexposed in light blue. For controls, *n* = 10; for ALS samples, *n* = 9

Almost all, 99%, of the mapped unique eccDNAs in the *hSOD1*^*G93A*^ and control samples covered eccDNA sizes of < 10^4^ bp. Detailed length-stratified profiling, as exemplified in the histograms of Fig. 2A, and illustrated for all control and ALS samples in the histograms of Additional file 2: Fig. S2A-B, revealed that the majority of eccDNAs were smaller than ~ 10^4^ bp in size (Fig. 2A; Additional file 2: Fig. S2A, B). Overall size distributions of eccDNAs were similar for *hSOD1*^*G93A*^ and control tissues (Fig. 2A, B; Additional file 2: Fig. S2A, B). Notably, in both groups eccDNA exhibited a periodic enrichment with a size-interval of approximately every ~ 200 bp (Fig. 2C), which is in accordance to prior indications of 188 bp regular intervals in mouse embryonic stem cells (mESCs) [68]. Whether such consistent observations can indicate an association with the nucleosome repeat length (NRL) in mouse, comprising the 147 bp of core nucleosomes and their linker stretches, needs further investigations. NRL varies between species, cell types and genomic locations, but can occupy more than 200 bp in mouse [69].

Based on our genotoxic stress model, we anticipated eccDNA accumulations in spinal cord of the *hSOD1*^*G93A*^ mutant. To verify this notion, we assessed the sample-individual load of eccDNA species for each group. Irrespective of their absolute quantities, which are not assessable from RCA, the numbers of unique, mappable eccDNAs across all chromosomes up to a size of 10^5^ bp were defined. These ranged from 94 to 1501 eccDNAs in controls (μ ± SEM = 480.7 ± 137.3) up to 1377–5610 eccDNAs in ALS samples (μ ± SEM = 2882.6 ± 419.3), revealing a statistically significant, six-fold increase in the number of unique eccDNAs under disease conditions (*p*-value = 2.165^–05^; Fig. 2D) (where μ is the mean and SEM the standard error of the mean). We next explored the chromosomal origin of the eccDNA species prevailing in the CNS, and particularly in presence of the *hSOD1*^*G93A*^ mutation. In compliance with a genome-wide impact of the SOD1 enzyme dysfunction and endogenous ROS on chromatin structures, eccDNA provenience was genomically scattered in both the ALS and control samples and arose from all 19 murine autosomes (Fig. 2E; Additional file 3: Fig. S3A, B).

### Split-reads based differential analysis identifies a distinctive genic eccDNA profile in ALS spinal cord

**Fig. 3:**
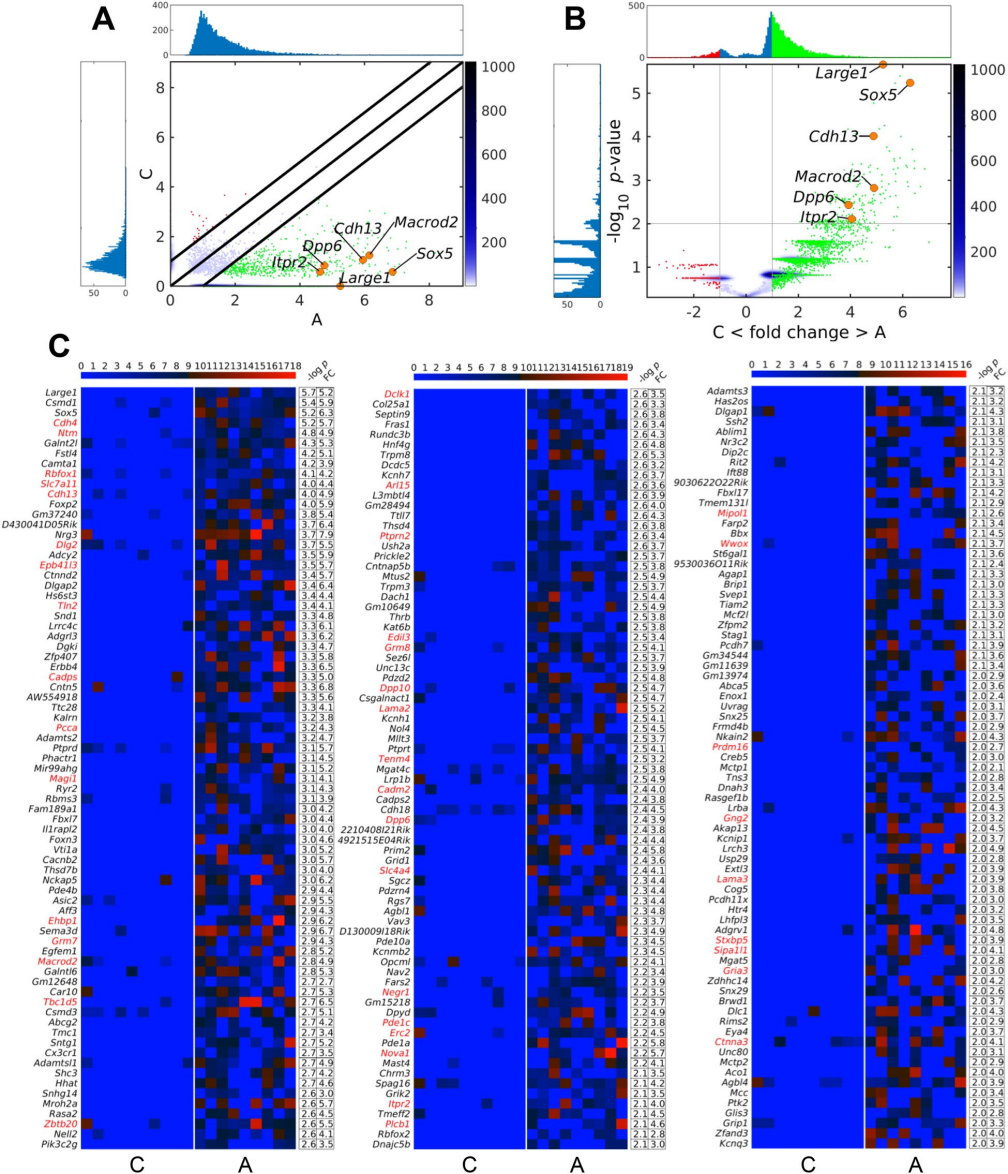
Identification of a distinctive eccDNA profile in ALS based on split-reads differential analysis. **A** For differentiation of PpGCs in ALS and control samples, boundaries were set at log_2_ FC 1 (FC 2 in linear scale) as indicated in the pairwise scatter plot by diagonal black lines. PpGCs spectra are visualized by histograms (blue). Those up- or down-produced in abscissa compared with ordinate samples are shown with green and red dots, respectively; orange dots indicate significant DPpGCs from selected genes. Color bar depicts scattering density, with darker blue corresponding to higher scattering density. PpGCs levels are scaled in log_2_. **B** Volcano plot of the PpGCs in ALS *versus* control samples. The vertical black lines are the boundaries of the log_2_ FC 1 thresholds in the eccDNA production levels between the groups. The horizontal black line is the 2-boundary of the *p*-value in -log_10_ scale (corresponding to *p*-value = 0.01 in linear scale) in ALS *versus* control samples. PpGCs beyond these thresholds were inferred to be significantly distinct from control eccDNAs and denominated as DPpGCs. Their distributions are visualized by histograms. DPpGCs up-produced in ALS samples are indicated with green dots; orange dots indicate the most significant DPpGCs. **C** Heatmaps of the 225 genes that led to up-DPpGCs formations in ALS relative to control samples. Color bars codify the value of PpGCs in log_2_ scale. Higher level of difference corresponds to a redder color. The -log_10_ (*p*-value) of the up-DPpGCs and the FC in log_2_ scale are presented in a two-column table to the right of the respective heatmaps. These annotations are displayed in descending order of statistical significance. Gene names in red mark the cases with corresponding proteome changes. A-C: C, control samples; A, ALS samples

To assess if any of the genes in the mouse genome were particularly prone to form eccDNA under the two different conditions, we calculated the PpGC for the control and ALS samples. A heatmap of the top 1000 most variable PpGCs is shown in Additional file 4: Fig. S4. The paired scatter plot in Fig. 3A discloses differences in the PpGCs produced in the *hSOD1*^*G93A*^ mutants and control specimens. For a log_2_ fold change (FC) > 1 (FC ≥ 2 on the linear scale) and a -log_10_ *p*-value ≥ 2.0 (significance level α = 0.01) as statistical thresholds, we identified 225 up-DPpGCs, corresponding to genes that were particularly prone to form eccDNA under ALS conditions, *i.e.*, in an ALS-specific pattern (Fig. 3B). No up-DPpGCs were identified in the controls relative to ALS for the same thresholds.

**Fig. 4:**
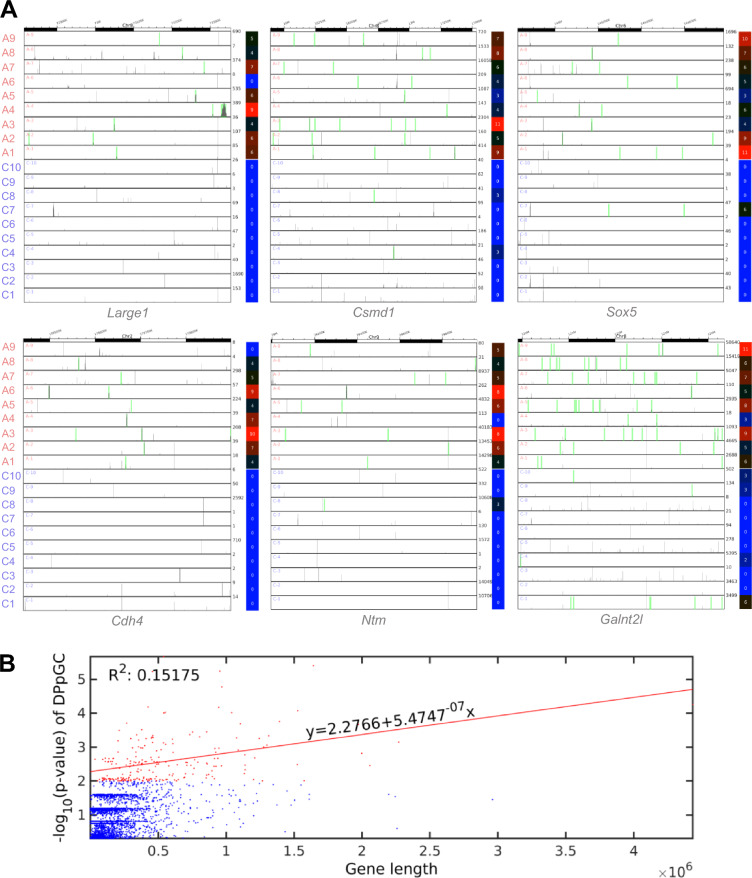
Coordinates of eccDNAs hotspot *loci*, gene coverages and relation to gene lengths. **A** Track plots of the *loci* of the six top-ranked up-DPpGCs in ALS. The genes and corresponding gene coverages represent the results in relation to controls. Each horizontal line represents the length of a gene. The green bars represent the deletion/excision *loci* of the eccDNA. The color bars codify the value of PpGCs in a log_2_ scale. **B** Relation between the -log_10_ (*p*-value) of the DPpGCs from ALS spinal cord and the size of the underlying gene. Red dots discriminate the 225 statistically significant up-DPpGCs in ALS from all other PpGCs (blue). The red line is the regression line of the -log_10_ (*p*-value) of the statistically significant up-DPpGCs in ALS in function of the respective gene lengths

The top up-DPpGCs were shed by the following gene *loci*: *Large1* (-log_10_ *p*-value = 5.7, FC = 5.2), *Csmd1* (-log_10_ *p*-value = 5.4, FC = 5.9), *Sox5* (− log_10_ *p*-value = 5.2, FC = 6.3), *Cdh4* (− log_10_ *p*-value = 5.2, FC = 5.7), *Ntm* (− log_10_ *p*-value = 4.8, FC = 4.9), and *Galnt2l* (− log_10_ *p*-value = 4.3, FC = 5.3), as indicated in Fig. 3C. EccDNA release from each of these 6 hotspot *loci* was consistently detected in at least 8 out of the 9 ALS samples investigated (Fig. 3C). In Fig. 4A, these 6 top up-DPpGCs are further illustrated as ‘linear’ circle track plots together with their coverages, while solely the linear circle plots are shown for all remaining up-DPpGCs in Additional file 5: Fig. S5. The linear plots illustrate that up-DPpGCs are represented by eccDNA carrying gene fragments only but no full genes, and the lack of sequence overlap of genic eccDNAs among the samples.

When considering gene size, we found these 6 hotspot genes to be large and, except for *Large1*, to be exclusively or dominantly transcribed in CNS and skeletal muscle cells, as deduced from single cell cluster expression patterns annotated in the Human Protein Atlas (2022) for different tissues and cell types. To explore a putative linear correlation between the probability to excise eccDNA and the originating gene length, the -log_10_ (*p*-values) of the 225 hotspot genes were plotted against the size of the corresponding genes. The resulting regression line (Fig. 4B) and correlation coefficient of *R*^*2*^ = 0.15175 indicated that, though long genes were involved, the propensity to shed eccDNA in our ALS model was not correlated with the size of the underlying genes.

### Association of eccDNA gene hotspots with ALS risk loci shows 63 genes in common

**Fig. 5:**
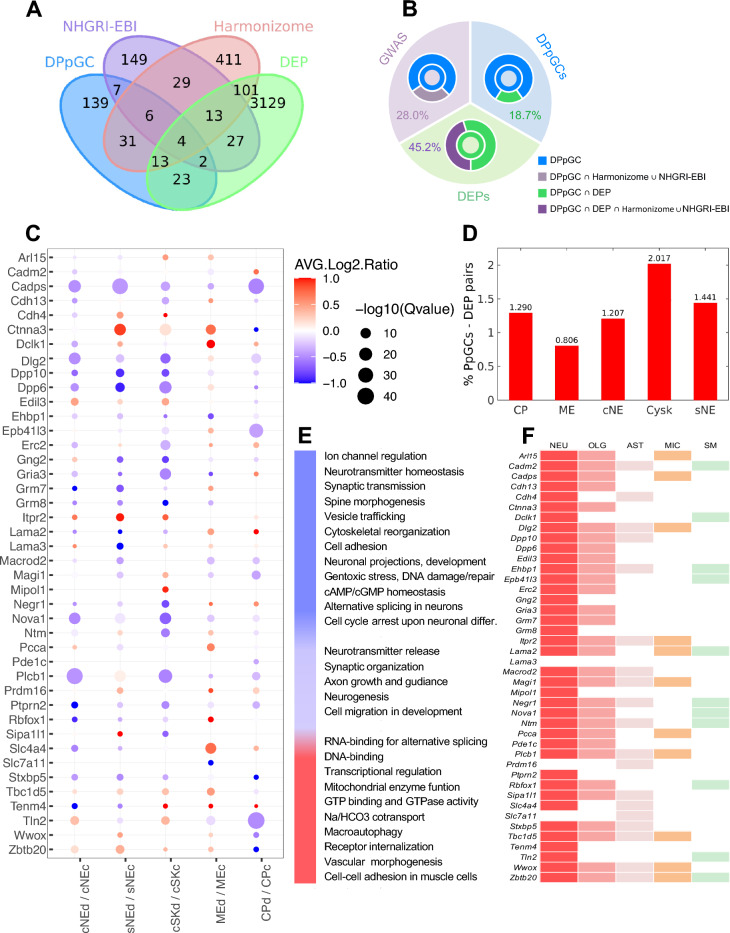
Association of up-DPpGCs with ALS proteome changes and ALS risk genes. **A** Venn diagram displaying intersections of DPpGCs up-produced in ALS with NHGRI-EBI Catalog of human GWAS, Harmonizome database, and the ALS DEPs. **B** Overlap of DPpGCs (blue) with proteomic DEPs (green) and GWAS ALS risk gene annotations (purple). In total, 28.0% of DPpGC related genes conveyed an ALS risk in GWAS, and 18.7% showed a DEP pendant; an additional ALS risk annotation for those with DEP partner was found for 45.2%. **C** Pair-correlation of DPpGCs and DEPs in ALS. Out of the 225 DPpGCs, 42 showed altered protein abundance. While all DPpGCs were up-produced, DEPs were up- or down in content. DPpGCs with strongest up-regulation (large dot diameters) predominantly showed discordant DEP regulation (blue in color code). Up-DPpGCs and DEPs are illustrated in red; down-regulated DEPs are marked in blue. *sNE* soluble nuclear fraction, *cNE* chromatin-bound nuclear fraction, *Cysk* cytoskeletal fraction, *CP* cytoplasmic fraction, *ME* membrane fraction. *n* = 9 for DPpGCs; *n* = 5 for DEPs. *p*-value for DPpGCs ≤ 0.01; *q*-value for DEPs < 0.05. Threshold average log_2_ ratio for DEPs > 0.38. **D** Percentages of DPpGCs pair-correlated with DEPs in the 5 proteomics fractions in ALS. **E** Cellular functions of the 42 DPpGC—DEP tandems, according to GeneCards (https://www.genecards.org/cgi-bin/carddisp.pl?gene). Pairs conveying neuronal and ALS-counteracting functions were primarily down-regulated (blue on color bar), while those mediating a more general cell biology were up-regulated (red on color bar). Some functional categories were either up- or downregulated in the different cellular subfractions (purple on color bar). **F** Color-encoded in the map are the cell types with enriched expression of the individual genes, as assessed from the Human Protein Atlas. The cell type with clustered expression is indicated in the heading. White background color indicates no clustering. NEU, neurons, OLG, oligodendrocytes; AST, astrocytes; MIC, microglia; SM, skeletal muscle cells

For a comprehensive assessment of whether the 225 up-DPpGCs carry a signature linked to the ALS pathophysiology, we referenced them to publicly available databases that aggregate genetic variants associated with fALS/sALS disorders from genome wide association studies (GWAS). In total, 610 gene sets were notified in association with the term ‘amyotrophic lateral sclerosis’ in the integrative Harmonizome database, which collects information for human and mouse species from GWAS and other genetic association studies and displays empirical cumulative probabilities for the 10% with strongest associations in a standardized fashion [70]. Applying the ‘GWASdb SNP-Disease Associations’ setting for output search [70], 608 genes were found listed in association with the disease ‘amyotrophic lateral sclerosis’. Among, we found 54 listed genes to overlap with our 225 up-DPpGCs, 3 of which, *Csmd1, Sox5, Cdh4* were among the aforementioned 6 up-DPpGCs with highest statistical significance (Fig. 5A, B). The total list is found in Additional file 7: Table S1. Notably, all of these disease-associated genes were also positively screened in the category of ‘GWASds SNP-Phenotype Associations’, indicating relevance as for symptom manifestation and disease penetrance.

In the context of a second, high-quality curated repository, the National Human Genome Research Institute—European Bioinformatics Institute (NHGRI-EBI) Catalog of human genome-wide association studies [71], approximately 350 variant associations were displayed for the trait ‘ALS’, underlying defined GWAS curation cutoff values of *p*-value ≤ 1.0 × 10^–5^ for single SNP-traits as described in the recently updated deposition resource [71]. From this database collecting human associations only, we could extract 237 unique genes associated with ALS (state: 16/01/2023), 19 of which were overlapping with our 225 up-DPpGCs (Fig. 5A, B; Additional file 7: Table S1). These comprised the genes *Adamtsl1, Asic2, Camta1, Creb5, Ctnnd2, Dach1*, *Dpp6, Erbb4, Grid1, Itpr2, Kalrn, Kcnmb2, Lama2, Lama3, Macrod2, Mir99ahg, Opcml, Ptprn2,* and *Trpm8*. Notably, 10 of them, *Asic2*, *Ctnnd2*, *Creb5*, *Erbb4*, *Itpr2*, *Kalrn*, *Lama3*, *Macrod2*, *Opcml*, and *Ptprn2*, were recorded both in the Harmonizome and NHGRI-EBI study assortment (Fig. 5A, B; Additional file 7: Table S1). In total, we found 63 out of the 225 up-DPpGCs, 28.0%, as being indexed in these two databases (Fig. 5A, B; Additional file 7: Table S1). The convergence of ALS risk gene considerations established in experimental studies and human GWAS with our eccDNA analyses is comprehensively illustrated in Fig. 5A and B and detailed in Additional file 7: Table S1.

### EccDNA levels in ALS correlate with the ALS proteome

To investigate whether genes prone to form eccDNA were also affected at the proteome level, we assessed changes in protein abundances in *hSOD1*^*G93A*^ and control spinal cord via mass spectrometry. Proteins found to be differentially expressed in the ALS proteome when referenced to the healthy control proteome (*q*-value < 0.05; absolute average log_2_ ratio > 0.38; Fig. 5C) were extracted from a cell compartment-specific analysis (Fig. 1F, G). In total, 3312 differentially expressed proteins (DEPs) were identified. Purity of the 5 subcellular fractions harvested was recently confirmed mass spectrometrically by marker proteins (unpublished data). Overall, when relating our ALS-specific catalogue of eccDNA hotspot genes to the ALS sub-proteome in the respective cell compartment, we found that the highest percentage of eccDNA releasing genes in ALS had counterparts in the Cysk (2.0%) and sNE (1.4%) fractions, followed by comparable percentages in the CP and cNE fractions (1.3% and 1.2%), and lowest in the ME fraction (0.8%) (Fig. 5D).

In total, 42 out of the 225 up-DPpGCs, *i.e.*, 18.7%, exhibited a coincidentally altered protein level under ALS relative to control conditions in at least one of the cellular subfractions (Fig. 5A–C). While all involved genes were up-producing eccDNAs in the *hSOD1*^*G93A*^ mutants compared to controls, protein counterparts were either up- or downregulated (Fig. 5C). DEP pendants for the up-DPpGCs with strongest *q*-values (-log_10_ scaled) were mainly discordantly down-regulated (Fig. 5C). Seventeen of these 42 DPpGC—DEP tandems overlapped with ALS-associated gene annotations in the Harmonizome database, including *Cadm2, Cdh4*, *Cdh13, Ctnna3, Dclk1, Gria3, Grm7, Grm8, Itpr2, Lama3, Macrod2, Plcb1, Prmd16, Ptprn2, Rbfox1, Wwox,* and *Zbtb20* (Fig. 5A, B; Additional file 7: Table S1), two of which, *Macrod2*, and *Itpr2*, also intersected with the human GWAS catalogue (Fig. 5A, B; Additional file 7: Table S1). Additionally, the DPpGC—DEP pairs related to *Dpp6* and *Lama2* were found registered in the human GWAS database (Fig. 5A, B: Additional file 7: Table S1). Thus, 19 out of 42, *i.e.*, 45.2% of the DPpGC—DEP couples were ascribed to an ALS risk (Fig. 5A, B; Additional file 7: Table S1), indicating triple DPpGC-DEP-GWAS risk associations. In comparison to the 28.0% of all up-DPpGCs that were overlapping with ALS risk gene annotations (Fig. 5A, B; Additional file 7: Table S1), these protein correlations strengthened our preceding indications of an ALS-related, *SOD1*^*G93A*^- coined eccDNA profile in the spinal cord of affected animals. Prevailing functional denotations associated with the up-DPpGC—down-DEP pairs, according to GeneCards^®^ annotations complemented by manual research, are summarized in Fig. 5E. Itemized are neurotransmitter homeostasis, synaptic transmission, establishment of neuronal projections, cytoskeletal reorganization, cAMP/cGMP homeostasis and DNA repair, all of which are of relevance in the ALS pathology. Screening of single cell expression clusters based on the Human Protein Atlas (2022) proved that the genes underlying these DPpGC—DEP tandems were predominantly expressed in neurons and glia entities, and some of them showed neural and muscular clusters (Fig. 5F).

**Fig. 6:**
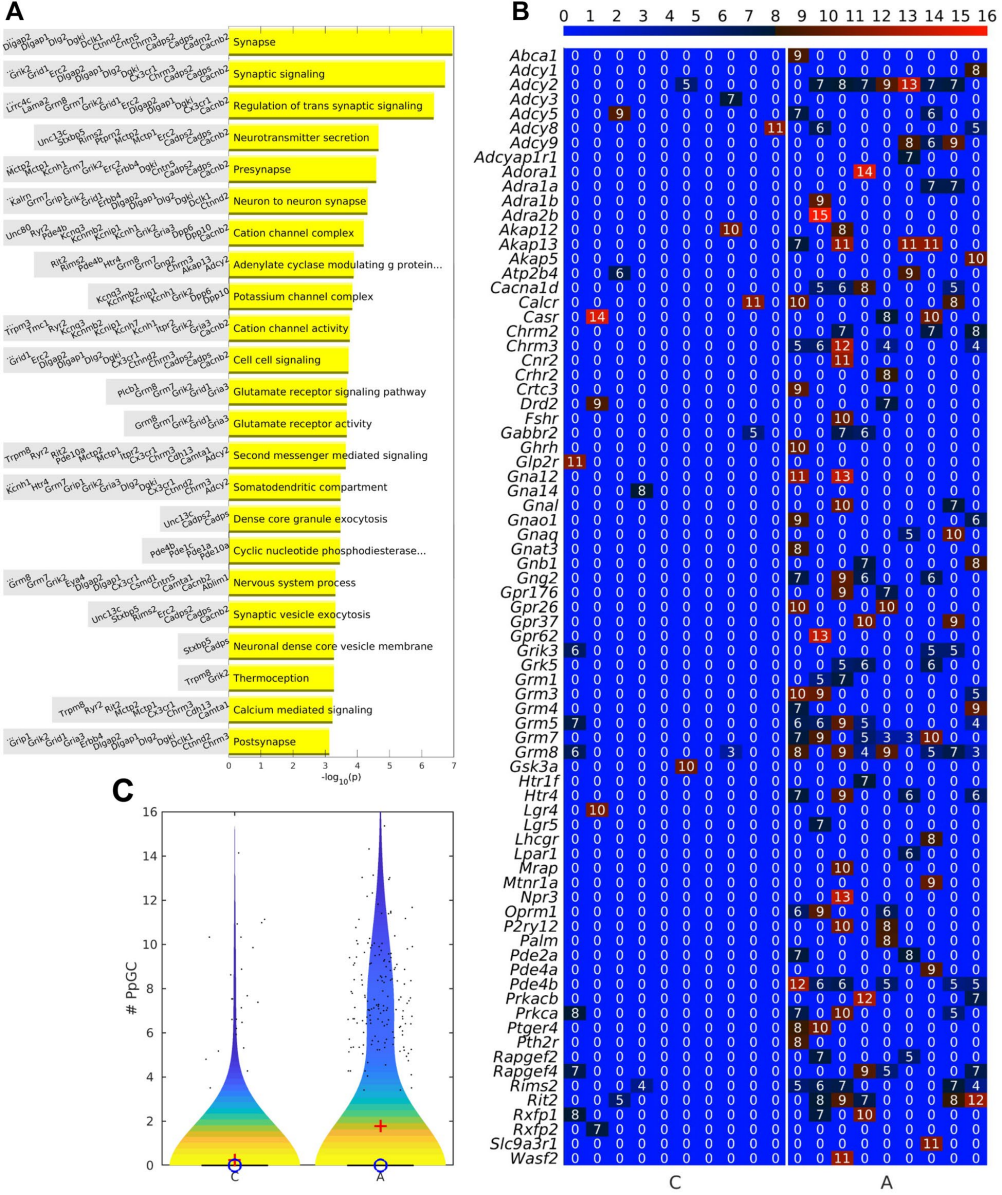
Gene set enrichment analyses (GSEA) for the 225 DPpGCs up-regulated in ALS. **A** Functional categories and GSEA representing genes ranked according to their log2 (*p-*values). **B** Heatmap of all eccDNA embedded genes (DPpGCs and PpGCs) that are related to the adenylate cyclase modulating G protein signaling pathway found enriched by the GO term analyses given in (A). **C** Violin plot of the eccDNA-related genes assigned to the GO term ‘adenylate cyclase modulating G protein pathway’ in the control and ALS samples. The graph reveals a numerical enrichment of gene representants in ALS

Additionally, the majority of DPpGCs that remained devoid of a DEP pendant were generated from neuronal genes, as indicated by gene set enrichment analyses (GSEA) (Fig. 6A). Among the gene ontology (GO) terms enriched, our analyses discovered the class of ‘adenylate cyclase modulating G proteins’ as an overrepresented category that included 10 related genes, *Adcy2*, *Akap13*, *Chrm3*, *Gng2*, *Grm7*, *Grm8*, *Htr4*, *Pde4b*, *Rims2* and *Rit2* (Fig. 6A), all of which up-produced DPpGCs under ALS conditions. The entire fraction of PpGCs generated from genes of the adenylate cyclase signaling pathway are displayed in Fig. 6B, and their numerical overrepresentation is illustrated in the violin plot of Fig. 6C.

### Higher number of PpGCs from RDC neural coding genes in ALS than in controls

**Fig. 7:**
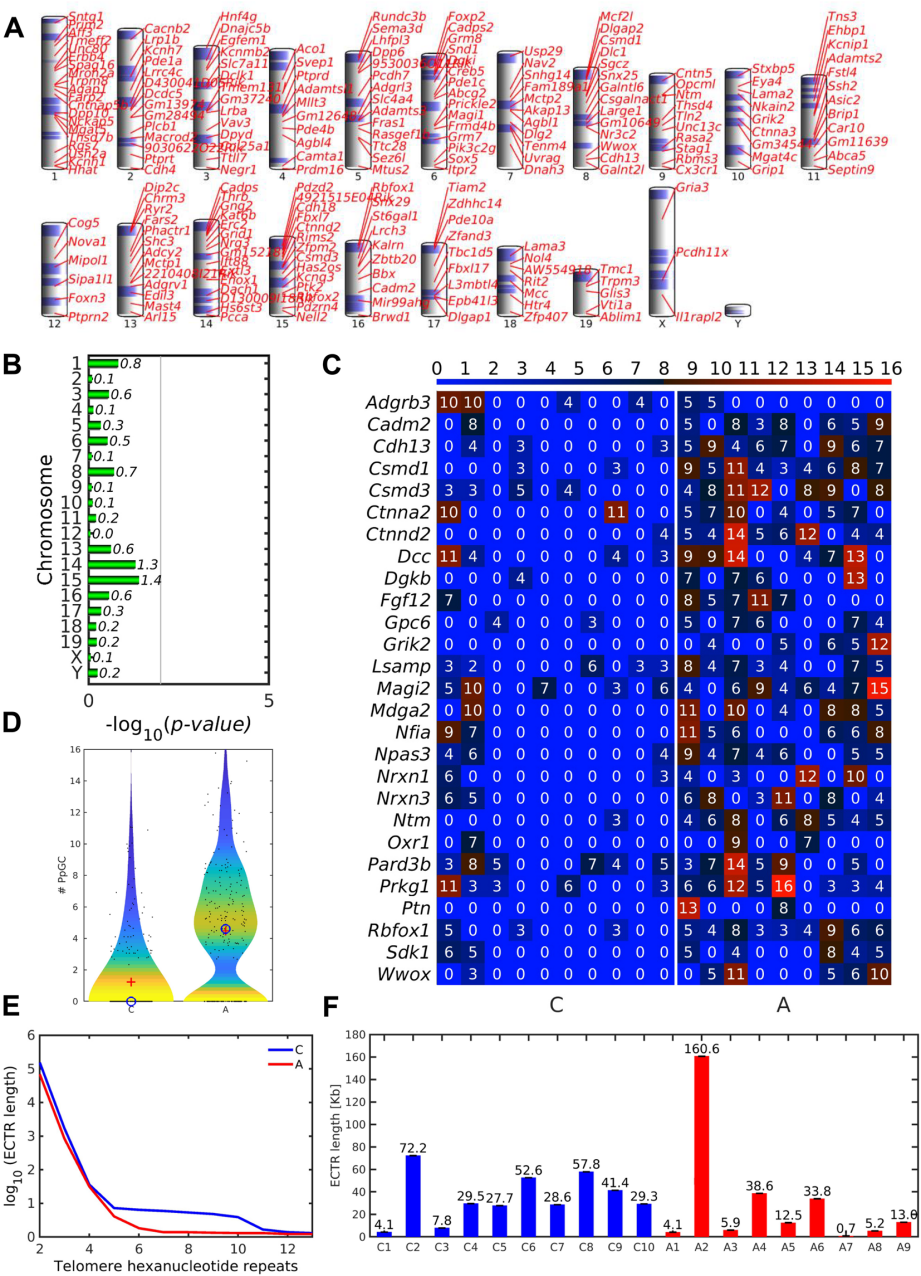
Chromosomal mapping of DPpGCs, of PpGCs in RDC genes, and telomere related ECTR. **A** Chromosomal landscaping of the genomic *loci* giving rise to statistically significant up-DPpGCs in ALS relative to controls. **B** Chromosome-specific up-DPpGC enrichment scores in ALS. **C** Heatmap of the PpGCs derived from the 27 RDC-associated neural coding genes identified in mice [73], as detected in control and ALS samples. **D** Violin plots illustrating the distribution of the PpGCs from the 27 RDC-associated neural coding genes in controls and ALS. The blue circle and the red cross mark the position of the median and the mean of the distributions, respectively. **E** Length (log_10_) of repetitive telomere structures (ECTR) on eccDNA estimated for different numbers of hexanucleotide repeats in control (blue) and ALS samples (red). **F** Length of ECTR estimated for at least 4 hexanucleotide repeats for each control (blue) and ALS (red) sample per group. C, control samples; A, ALS samples; ECTR, extrachromosomal telomere repeats. For ALS samples, *n* = 9; for control samples, *n* = 10

To explore a putative preponderance in eccDNA production by chromosome structure as found described recently [72], the parent *loci* of the 225 up-DPpGCs in ALS were mapped back to the murine chromosomal set (Fig. 7A). No chromosome proved to be significantly enriched in up-DPpGC shedding (Fig. 7B).

Predisposing factors for genes to liberate eccDNA can be structural or functional, and might be associated with susceptibility *loci* for DSB. For mice, 27 neural coding genes are known to harbor recurrent DSB clusters (RDC), as demasked under conditions of deficient DNA repair and replication stress [73]. Ten of these 27 RDC genes (37.0%), *i.e., Csmd1, Csmd3, Ntm, Cdh13, Magi1, Grik2, Rbfox1, Ctnnd2, Cadm2 and Wwox* also occurred in our list of up-DPpGCs in ALS, emphasizing the role of genomic instability in ALS-related circular DNA production. We assessed the differences in PpGCs between ALS and controls for the complete set of these 27 RDC genes (Fig. 7C) and found a higher number of RDC-related PpGCs in ALS than in control specimens (Fig. 7C, D), implicating a FC of 3.6185 for the ratio built from the ALS and control means (*p*-value = 3.1704^–26^).

### No difference in extrachromosomal telomere repeats between ALS and controls

Telomeres are particularly susceptible to oxidative stress compared to the bulk genome [74]. We therefore tested whether telomeres were more prone to form eccDNA in the ALS model by estimating the sample-specific length of telomeric TTAGGG minisatellites on eccDNA circles, termed extrachromosomal telomere repeats (ECTR). We observed slightly longer ECTR in control as compared to ALS samples (Fig. 7E), with the difference being more pronounced for the range of 5–10 repeats (Fig. 7E). To estimate the total ECTR length, a sequence of 4 hexanucleotide repeats was chosen since this length was not exceeded in most samples. The mean total TTAGGG repeat length on ECTR showed high inter-sample variability (Fig. 7F) but was predicted to be 35.1 ± 6.8 Kbp (*n* = 10) for control samples and 30.5 ± 16.9 Kbp for ALS samples (*n* = 9) and thus was comparable between the groups (*p-*value = 0.397; Fig. 7F).

### Common PpGCs in ALS are frequent and predominantly specific

**Fig. 8:**
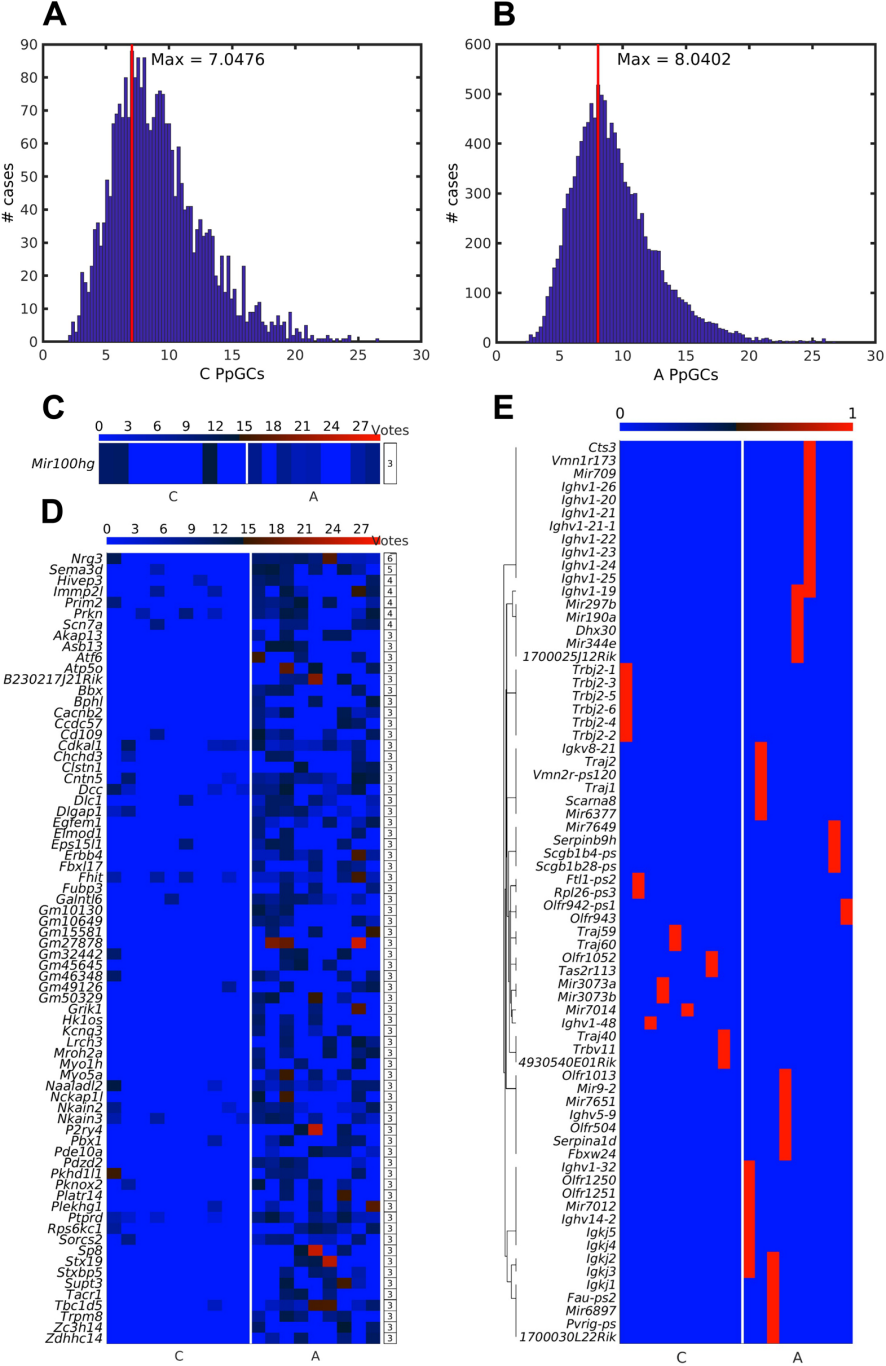
Inter-group distribution of the common PpGCs (CPpGCs) and full gene eccDNAs. **A**–**B** Histogram of all PpGCs in (**A**) control and (**B**) ALS samples. The vertical red line shows the position of the maximum of the distribution. **C**–**D** Heatmaps of the CPpGCs in (**C**) control and in (**D**) ALS as obtained with the democratic method. The color bars codify the value of the PpGCs in a log_2_ scale. The number of votes for each CPpGC is presented in tables to the right of the heatmaps. **E** Boolean heatmap of the entirety of whole coding genes embedded in eccDNA in any of the samples. Blue and red colors correspond to the presence and absence of full genes, respectively. In A-E: C, control samples; A, ALS samples

To further identify PpGCs that are common either in the control or ALS group, denoted as CPpGCs, we employed the democratic method that shows a group-intrinsic over-representation independently of an inter-group differentiation. To find the minimum eccDNA production values to be considered as a positive vote, the empirical distributions of the PpGCs were assessed for ALS and control samples (Fig. 8A, B). As threshold parameter served the ceiling of the maximum, which was 8 and 9 for control and ALS, respectively. Considered were all genes that cumulated at least 3 votes (Fig. 8C, D).

In total, we identified 72 CPpGCs in ALS (Fig. 8D), 25 of which were among the DPpGCs already retrieved with the DifCir algorithm. Notably, the CPpGCs derived from the *Scn7a* gene and those originating from the *Cdkal1*, *Chchd3*, *Dcc*, *Fhit*, *Naaladl2*, *Pbx1*, and *Sorcs2* genes intersected with the NHGRI-EBI and Harmonizome databases, respectively. The remaining expanded the ALS eccDNA profile. By contrast, only one CPpGC was detected in control samples, *Mir100hg* (Fig. 8C). The miRNAs deriving from the *miR-100/let-7a-2/miR-125b-1* cluster host gene, *Mir100hg*, are transcribed in spinal cord and upregulated in presence of the *hSOD1*^*G93A*^ mutation [75]. Likewise, *miR-125b-5p* is induced in FUS-mutated motor neurons and therein associated with an impaired DDR and DNA damage accumulation [76]. According to such role, CPpGC from this gene was also found in the ALS samples. Hence, it was not specific for healthy spinal cord tissue. Notably, miRNAs from this cluster can also modulate inflammatory responses that might be relevant for ALS [77, 78].

### EccDNA carry full genes

When screening all eccDNAs for full gene sequences, independently of thresholds for differential production and sample types, we gathered replicates of 235 uncropped genes or pseudogenes (Additional file 6: Fig. S6, A, B), 69 of which carried coding functions. Notably, 100% of the ALS samples provided full gene eccDNAs excised from numerous gene *loci* (μ ± SEM = 0.085 ± 0.011; Fig. 8E). By contrast, 80% of the control samples embedded full genes on their circles, involving a minor number of gene *loci* (0.028 ± 0.0062; Fig. 8E). These data attest a significantly higher probability to excise full gene sequences from the ALS than from the control genome (*p-*value = 4.19^–6^). Regarding inter-sample recurrence, the *immunoglobulin κ joining-2 and -3* (*Igkj2*, *Igkj3) loci* were iterated by two ALS samples (Fig. 8E) and thus showed minor sample overlap. As the underlying highly repetitive, polymorphic *locus* is subjected to V-D-J recombination, class switch recombination and somatic hypermutations [79], all of which are engaged in eccDNA genesis, it might reflect a general source of circular DNA synthesis, unrelated to the ALS pathophysiology, or originate from residual blood cells in the extracted samples. Accordingly, most prominently represented were the lymphocyte-related *Tra/Trb* and *Igh/Igl loci* encoding B- and T-cell receptor specificity in response to pathogens and other antigens. A second cluster relied in sensory receptor signaling engaging the extremely large *Olfr* multi-gene family [80]. In conclusion, as lacking intra-group re-occurrence and presenting with single-event eccDNA, whole genes did not contribute to ALS hotspot *loci*.

## Discussion

The role of circular DNA in CNS disorders and in clinically and genetically heterogeneous ALS [81] is undefined. Here, we show that in spinal cord of symptomatic *hSOD1*^*G93A*^ mutant mice mimicking an ALS phenotype the release of eccDNA is several-fold increased, especially from 225 genes. Notably, the inter-sample iteration rate of the statistically top 6 eccDNAs showing a profiled overproduction uniquely in the ALS group was 89–100%. Among the 225 hotspot genes, 63 were found associated with an ALS risk in GWAS [70, 71], and 42 paralleled an altered abundance at the protein level. A dual itemization in ALS risk datasets and change at the protein level applied to 45.2% of the DPpGC—DEP tandems. Functional interrogations indicated that many of these pairs carry specific roles in CNS and neuromuscular cell biology, such as in glutamate homeostasis, synaptic transmission, neurotransmitter release, development of axonal projections, cytoskeleton organization and DNA damage and repair. In contrast to the eccDNA—GWAS alignments, these DPpGC—DEP couples exhibited an even more specific pattern in that they covered neuronal processes with capability to counteract the damage profile incurring in ALS, as being coined by synapse loss, calcium dyshomeostasis, degradation of the cytoskeletal architecture and DNA damage. Broader eccDNA-related functions, though also with an established role in ALS, encompassed regulations of DNA and RNA binding, RNA splicing, gene transcription, mitochondrial enzyme function and autophagy. As the eccDNA-corresponding gene products with a specific neuro-muscular location and function were primarily reduced in the ALS proteome, up-production of mapping eccDNA species might reflect increased DNA damage in these genes, as they might be preferentially transcribed to compensation for the ALS-related functional loss. This scenario might hence propagate a loss-of-function vicious circle. By contrast, most of the gene products involving ubiquitous biological relevance were up-produced both at the eccDNA and protein level.

Irrespective of protein pendants, GSEA of our 225 hot spot genes identified the adenylate cyclase modulating G protein coupled receptor signaling pathway to be among the most enriched GO terms, involving at least 10 related genes, *i.e.*, *Adcy2*, *Akap13*, *Chrm3*, *Gng2*, *Grm7*, *Grm8*, *Htr4*, *Pde4b*, *Rims2*, *Rit2*. G-protein coupled receptors (GPCR) belong to a large heterogenous membrane receptor family that transmits cellular signals through the modulation of adenylyl cyclase activity, followed by changes in the intracellular concentration of cyclic AMP (cAMP). Proteins that interfere with GPCR signaling can modulate the cGAS–STING pathway, a pro-inflammatory cascade that serves as an immune-sensor of ectopic, primary double-stranded DNA [for review, see 82, 83]. Upon binding of ectopic DNA, the cyclic GMP-AMP synthase cGAS catalyzes GTP and ATP conversion into GMP-AMP (cGAMP), which binds to STING, a stimulator of type I interferon (IFN) genes. Consecutive type I IFNα/β responses are reciprocally suppressed via TGF-β signaling [84]. Apart from the GO enrichment of GPCR, we identified several up-DPpGCs in ALS, *Thsd4*, *Dach1*, *Wwox, Prdm16, Mgat5*, and one miRNA gene, *Mir100hg,* in the category of CPpGCs in ALS and controls that operate in the antagonization of TGF-β signaling [77, 85–89]. This link could mean that eccDNAs, in their entity as ectopic DNA, influence innate immunity via cGas–STING in ALS. In support, both GPCR and cGAS–STING targeting has recently been discussed as a putative novel therapeutic target in ALS spectrum disorders [48, 51, 90]. Derepression of cGAS–STING might have particular relevance in *hSOD1*^*G93A*^-related ALS as oxidative damage triggers resistance towards TREX1 nuclease activity, which degrades ectopic and extrachromosomal DNA [91].

Whether the large muscular dystrophy gene ‘like-acetylglucosaminyltransferase’, or *Large*, which was among the top eccDNA hot spot genes, might have a role in ALS, deserves further explorations. Mutations in the *LARGE* gene, encoding a glycosyltransferase essential for dystroglycan glycosylation, causes muscular dystrophy in humans that is also entailed by defects in signal transmission at neuromuscular junctions and CNS pathologies.

As a third observation linked to ALS, among the eccDNA—DEP couples we identified several members of the *IgLON* family, *Negr1 (IgLON4)* and *Ntm (IgLON2)*, with *Ntm* ranking among the eccDNA hotspots with the strongest statistical regulation. *Opcml (IgLON1)*, which completes this family of cell adhesion molecules together with *IgLON5*, was also among the 225 eccDNA hotspots though missed a protein correlate. The human IgLON5 family is part of an immunoglobulin superfamily abundantly expressed in neurons, and which is related to the autoimmune-encephalitic and neurodegenerative Anti-IgLON5 syndrome, an important differential diagnosis in motor-neuron disease-like phenotypic presentations involving muscle weakness, stiffness and fasciculations [92–94]. Therefore, it appears tempting to assume that the accumulation of a non-random eccDNA collection is released in direct connection to the neuro-muscular pathology. Similarly, when assessing healthy skeletal muscle in humans, Møller *et al.* could identify the highly transcribed *TTN* gene as a hotspot from which eccDNA is released with higher probability than from other *loci* [37]. Whether such patterned eccDNA profile detected here operates as a direct genomic modifier with putative impact on the gene products and the disease course needs further evaluation, *e.g.*, via CRISPR-C-mediated phenotype characterizations [95]. The possibility that eccDNAs interact as si-like RNAs [46] or as mobile modifiers [47] with linear chromatin structure and ALS-related pathogenicity genes will be of great clinical momentousness. The high inter-sample repetition detected for the eccDNA hotspots might further allow these eccDNAs to serve as novel biomarkers in ALS, and have the potential to complement the recently defined cut-off values for neurofilament light chain [96] or the suggested biomarker role of miRNAs [97].

Neuronal genes are prominent as for their large sizes that predispose for RDC [73] and eccDNA release [72]. While our computational differential analysis method, DifCir, scales for gene length, we further referenced the eccDNA hotspots to corresponding gene sizes and ruled out a strong correlation. Thus, a bias by gene size does not explain the neuro-muscular dominance of expression and function of the 225 genes identified. Rather, these observations are in accordance with our hypothesis that eccDNA arises from genomic instability in the *SOD1*^*G93A*^-related ALS model. Also, a broad number of our hotspot genes were among the neural genes recently identified in neural stem and progenitor cells to form RDC as demonstrated under conditions of c-NHEJ repair deficiency and replication stress [73]. Though significantly associated with gene length in the study of Wei and colleagues, the moiety of large genes that harbored RDC accounted only for a small portion of all, actively transcribed neural target genes larger than 100 Kbp, and normalization for gene length still identified these genes as fragile *loci* [73]. Thus, gene size is apparently not a sufficient parameter to explain their preponderance for eccDNA or RDC formation. Mechanistically, as these genes are highly conserved between mouse and human, and biologically related to nervous system development and skeletal muscle maturation and related to an ALS risk in several instances, they might unmask ALS as a neurodevelopmental rather than a pure neurodegenerative disorder. In support is the continuing conjecture that ALS etiopathogenesis is initiated early in development and might also have a preclinical origin in stem and progenitor cell deregulations. The studies that currently address adult neurogenesis in ALS patients and rodent animal models are mainly carried out in the context of *SOD1* variants and suggest altered neural progenitor cell (NPC) proliferation, migration and commitment in adult neurogenic niches of brain and spinal cord [98–100]. As mutant NPC are particularly vulnerable towards oxidative stress [101], genomic instability in these lineage precursors might be a yet unconsidered factor in *SOD1*-related ALS manifestation. Further studies will elucidate whether RDC identified in mice can predict common fragile sites in ALS patients, possibly via eccDNA profiling.

Still, our study faces certain limitations. Evidence for the accumulation of patterned eccDNA is currently restricted to our *hSOD1*^*G93A*^ animal model of ALS. The phenotype in this mutant is expected to arise from a genotoxic gain-of-function in peroxidase activity rather than impaired SOD1 dismutase properties [7, 25]. Therefore, this study can profit from a second control group that overexpresses WT *hSOD1* with a copy number similar to the mutant allele. As WT *hSOD1* overexpression reduces oxidative stress and DNA damage under H_2_O_2_ exposure, it might, however, bear some risk to overestimate the effects obtained [7, 23]. Still, study validation in other ALS models entailing impaired genome stability and protection is required. Likewise, due to the propensity to form co-transcriptional R-loops at disease-relevant repetitive sites, *C9orf72* mutants will provide valuable information as for the contribution of DNA:RNA hybrids in circular DNA genesis also in other neurodegenerative repeat expansion disorders. Finally, expansion of eccDNA analyses to other non-replicative tissues and liquid biopsies will validate the CNS-specificity of the current observations and help set thresholds of eccDNA loads in ALS neurodegeneration. Further specifications in male and female mice will aid elucidate the sexual dimorphism encountered in younger ALS patients at the genome level and improve our understanding of sex-related genotype–phenotype presentations.

Methodically, our RCA- and short read-based algorithm is likely to underestimate long-read eccDNA. Therefore, strategies that allow the generation of high-confident consensus sequences of full eccDNA length, such as Nanopore or PacBio sequencing technologies will improve the characterization of eccDNA in the CNS. For a refined understanding of the eccDNA source mechanisms, analysis pipelines that resolve complex eccDNA composites from multiple break events are necessitated. Applying such advanced technologies, Wanchai and colleagues recently suggested that only 1–2% of the circles in murine cortical tissue comprise a complex, multi-locular genomic origin [102]. Accounting for the suggested capability to embed pathophysiologically impactful sequences, in analogy to the recently described regulator and enhancer functions of long ecDNAs in tumors [47], the modes of interaction with the linear genome will greatly broaden our understanding of small eccDNA in the context of neurological disorders. To allow conclusions in humans and to understand whether eccDNA can be an early causal event in ALS, as suggested for cancer [103], future investigations are required.

## Conclusion

Our study shows, for the first time, the accumulation of eccDNA in ALS neurodegeneration and is the unprecedented approach to couple episomal with genome-encoded variations in ALS. Given that the primal susceptibility to produce eccDNA originates from mutations in ALS-related genes that operate in genome protection, it might represent a yet unconsidered genomic factor on which clinically heterogeneous, but indiscriminate fALS and sALS entities converge on. Prospective studies covering quantitative and qualitative eccDNA profiles in other neurodegenerative disorders and in non-CNS tissues will reveal whether eccDNA can qualify for a diagnostic or predictive biomarker role in f/sALS and even have a causal role in neurodegeneration. In conclusion, eccDNA might add up a new, heteroplasmy-like factor that drives somatic mosaicism and contributes to the genetic, and putatively clinical heterogeneity in ALS.

### Supplementary Information

**Additional file 1: Figure S1. **Oxidative stress-induced chromatin changes in ALS motor neurons. **A** Representative multichannel photomicrograph of the axially transected cervical myelon in a control (Ctrl) and *hSOD1*^*G93A*^ (ALS) mutant. Rarified diseased SMI-32^+^ motor neurons (MNs; purple) of the anterior horn exhibited a higher frequency of mH2A1^+^ foci (green) in their nuclei (blue) than control MNs. The mH2A1^+^ chromatin foci are markers of DDR in response to oxidative DNA damage, with their location indicating the activation of PAR-dependent repair cascades. **B** Higher resolution single-channel images of an individual MN (dotted square) depicted in a control (Ctrl) and mutant (ALS) specimen. The nuclear Fucci red^+^ reporter signal assures the cell cycle-arrested, post-replicative state of SMI-32^+^ MNs. **C** Quantitative evaluation of mH2A1^+^ nuclear foci in control (Ctrl) and *hSOD1*^*G93A*^ mutant (ALS) MNs. Indicated *p*-values were assessed by two-way ANOVA. **p < 0.01. For each group, n = 3. Scale bar in A, B: 20 μm. Channel illustration in A, B engaged pseudo-coloring.

**Additional file 2: Figure S2.** Sample-individual distributions of length-sorted eccDNAs, related to Figure 2. The histograms indicate the eccDNA distributions up to a size of 10^4^ bp. Represented are the results for (A) control (C1-10; blue) and (B) ALS (A1-9; red) samples after removal of mt-DNA sequences and after merging and exclusion of eccDNAs with less than 2 split reads.

**Additional file 3: Figure S3. **Violin plots of the length distribution of eccDNAs < 10^5^ bp for all murine chromosomes and samples. Represented are the results after removal of mt-DNA sequences and after merging and exclusion of eccDNAs with less than 2 split reads. For (A) control (C1-10) and (B) ALS (A1-9) samples, the mean and median eccDNA read lengths cumulatively assigned to a certain chromosome are displayed as red crosses and green squares, respectively. Data points are overexposed in light blue. For A, n = 10 control and B, n = 9 ALS samples.

**Additional file 4: Figure S4. **Heatmap of the top 1000 most variable eccDNAs produced per gene (PpGCs). The color bar on the top codifies the PpGCs in log_2_ scale. Higher values of PpGCs correspond to redder color. C, control; A, ALS group.

**Additional file 5 Figure S5.** Topology of the 225 up-DPpGCs in the ALS versus control genome, related to Figure 4. Each linear circular DNA panel aligns the position (black rectangles) of the unique DPpGCs to their originating hotspot genes. Each horizontal line represents the length of a gene; the red lines correspond to ALS (**A**), the blue lines to control (**C**) samples.

**Additional file 6: Figure S6. **Whole coding genes on eccDNAs. **A** Chromosomal landscaping of the genomic *loci* giving rise to whole coding genes on eccDNAs specific for ALS conditions. The genes in potential *loci *clusters are marked in magenta. **B** List of whole coding genes on eccDNAs that are specific for control samples (blue) and ALS samples (red) or found in both groups (green).

**Additional file 7: Table S1. **Main intersections of the 225 up-DPpGCs with DEPs and with ALS-associated *loci *annotated in GWAS databases, according to the Venn diagram in Fig. 5A.

## Data Availability

The circulomics data discussed in this publication have been deposited in NCBI’s Gene Expression Omnibus and are accessible through GEO Series accession number GSE214718 (https://www.ncbi.nlm.nih.gov/geo/query/acc.cgi?acc = GSE214718). The mass spectrometry proteomics data have been deposited to the MassIVE repository with the dataset identifier MSV000091740. Further information and requests for resources and reagents should be directed to Marcos J. Araúzo-Bravo (mararabra@yahoo.co.uk) and Alexandra Kretz (alexandra.kretz@med.uni-jena.de).

## Abbreviations

(f/s) ALS: Familial/sporadic amyotrophic lateral sclerosis
BFB: Breakage fusion bridge
CNS: Central nervous system
c-NHEJ: Classical non-homologous end joining
CRISPR: Clustered regularly interspaced short palindromic repeats
CAS9: CRISPR associated protein 9
cGAS: Cyclic GMP-AMP synthase
CPpGC: Common produced per gene eccDNA
DDR: DNA damage response
DEP: Differentially expressed protein
DPpGC: Differentially produced per gene eccDNA
DSB: Double strand break
eccDNA: Extrachromosomal circular DNA
ECTR: Extrachromosomal telomere repeat
Fucci: fluorescent ubiquitination-based cell cycle indicator
GPCR: G-protein coupled receptors
GSEA: Gene set enrichment analysis
GWAS: Genome wide association studies
HR: Homologous recombination
hSOD1: Human superoxide dismutase 1
IFNα/β: Interferonα/β
mESC: Mouse embryonic stem cells
NPC: Neural progenitor cells
NRL: Nucleosome repeat length
NHGRI-EBI: National Human Genome Research Institute—European Bioinformatics Institute
PpGC: Produced per gene eccDNA
RCA: Rolling circle amplification
RDC: Recurrent double strand break cluster
ROS: Reactive oxygen species
SEM: Standard error of the mean
SNP: Single nucleotide polymorphism
SOD1: Superoxide dismutase 1
SpcDNA: Short polydispersed circular DNA
SSB: Single strand break
STING: Stimulator of interferon genes
TGF-β: Transforming growth factor β
WT: Wild type

## References

[1] Schumacher B, Pothof J, Vijg J, Hoeijmakers JHJ (2021). The central role of DNA damage in the ageing process. Nature.

[2] Walker C, Herranz-Martin S, Karyka E, Liao C, Lewis K, Elsayed W, Lukashchuk V, Chiang SC, Ray S, Mulcahy PJ (2017). C9orf72 expansion disrupts ATM-mediated chromosomal break repair. Nat Neurosci.

[3] Farg MA, Konopka A, Soo KY, Ito D, Atkin JD (2017). The DNA damage response (DDR) is induced by the C9orf72 repeat expansion in amyotrophic lateral sclerosis. Hum Mol Genet.

[4] Andrade NS, Ramic M, Esanov R, Liu W, Rybin MJ, Gaidosh G, Abdallah A, Del'Olio S, Huff TC, Chee NT (2020). Dipeptide repeat proteins inhibit homology-directed DNA double strand break repair in C9ORF72 ALS/FTD. Mol Neurodegener.

[5] Tsang CK, Liu Y, Thomas J, Zhang Y, Zheng XF (2014). Superoxide dismutase 1 acts as a nuclear transcription factor to regulate oxidative stress resistance. Nat Commun.

[6] Barbosa LF, Cerqueira FM, Macedo AF, Garcia CC, Angeli JP, Schumacher RI, Sogayar MC, Augusto O, Carrì MT, Di Mascio P, Medeiros MH (2010). Increased SOD1 association with chromatin, DNA damage, p53 activation, and apoptosis in a cellular model of SOD1-linked ALS. Biochim Biophys Acta.

[7] Sau D, De Biasi S, Vitellaro-Zuccarello L, Riso P, Guarnieri S, Porrini M, Simeoni S, Crippa V, Onesto E, Palazzolo I (2007). Mutation of SOD1 in ALS: a gain of a loss of function. Hum Mol Genet.

[8] Martin LJ, Chen K, Liu Z (2005). Adult motor neuron apoptosis is mediated by nitric oxide and Fas death receptor linked by DNA damage and p53 activation. J Neurosci.

[9] Mitra J, Guerrero EN, Hegde PM, Liachko NF, Wang H, Vasquez V, Gao J, Pandey A, Taylor JP, Kraemer BC (2019). Motor neuron disease-associated loss of nuclear TDP-43 is linked to DNA double-strand break repair defects. Proc Natl Acad Sci U S A.

[10] Konopka A, Whelan DR, Jamali MS, Perri E, Shahheydari H, Toth RP, Parakh S, Robinson T, Cheong A, Mehta P (2020). Impaired NHEJ repair in amyotrophic lateral sclerosis is associated with TDP-43 mutations. Mol Neurodegener.

[11] Giannini M, Bayona-Feliu A, Sproviero D, Barroso SI, Cereda C, Aguilera A (2020). TDP-43 mutations link amyotrophic lateral sclerosis with R-loop homeostasis and R loop-mediated DNA damage. PLoS Genet.

[12] Wang H, Guo W, Mitra J, Hegde PM, Vandoorne T, Eckelmann BJ, Mitra S, Tomkinson AE, Van Den Bosch L, Hegde ML (2018). Mutant FUS causes DNA ligation defects to inhibit oxidative damage repair in amyotrophic lateral sclerosis. Nat Commun.

[13] Wang WY, Pan L, Su SC, Quinn EJ, Sasaki M, Jimenez JC, Mackenzie IR, Huang EJ, Tsai LH (2013). Interaction of FUS and HDAC1 regulates DNA damage response and repair in neurons. Nat Neurosci.

[14] Naumann M, Pal A, Goswami A, Lojewski X, Japtok J, Vehlow A, Naujock M, Günther R, Jin M, Stanslowsky N (2018). Impaired DNA damage response signaling by FUS-NLS mutations leads to neurodegeneration and FUS aggregate formation. Nat Commun.

[15] Rulten SL, Rotheray A, Green RL, Grundy GJ, Moore DA, Gómez-Herreros F, Hafezparast M, Caldecott KW (2014). PARP-1 dependent recruitment of the amyotrophic lateral sclerosis-associated protein FUS/TLS to sites of oxidative DNA damage. Nucleic Acids Res.

[16] Fang X, Lin H, Wang X, Zuo Q, Qin J, Zhang P (2015). The NEK1 interactor, C21ORF2, is required for efficient DNA damage repair. Acta Biochim Biophys Sin.

[17] Higelin J, Catanese A, Semelink-Sedlacek LL, Oeztuerk S, Lutz AK, Bausinger J, Barbi G, Speit G, Andersen PM, Ludolph AC (2018). NEK1 loss-of-function mutation induces DNA damage accumulation in ALS patient-derived motoneurons. Stem Cell Res.

[18] Kannan A, Cuartas J, Gangwani P, Branzei D, Gangwani L (2022). Mutation in senataxin alters the mechanism of R-loop resolution in amyotrophic lateral sclerosis 4. Brain.

[19] Sun Y, Curle AJ, Haider AM, Balmus G (2020). The role of DNA damage response in amyotrophic lateral sclerosis. Essays Biochem.

[20] de Belleroche J, Orrell RW, Virgo L, Habgood J, Gardiner IM, Malaspina A, Kaushik N, Mitchell J, Greenwood J (1998). Copper, zinc superoxide dismutase (SOD1) and its role in neuronal function and disease with particular relevance to motor neurone disease/amyotrophic lateral sclerosis. Biochem Soc Trans.

[21] Li J, Song M, Moh S, Kim H, Kim DH (2019). Cytoplasmic restriction of mutated SOD1 impairs the DNA repair process in spinal cord neurons. Cells.

[22] Borchelt DR, Lee MK, Slunt HS, Guarnieri M, Xu ZS, Wong PC, Brown RH, Price DL, Sisodia SS, Cleveland DW (1994). Superoxide dismutase 1 with mutations linked to familial amyotrophic lateral sclerosis possesses significant activity. Proc Natl Acad Sci U S A.

[23] Brasil AA, Magalhães RSS, De Carvalho MDC, Paiva I, Gerhardt E, Pereira MD, Outeiro TF, Eleutherio ECA (2018). Implications of fALS mutations on sod1 function and oligomerization in cell models. Mol Neurobiol.

[24] Hayward LJ, Rodriguez JA, Kim JW, Tiwari A, Goto JJ, Cabelli DE, Valentine JS, Brown RH (2002). Decreased metallation and activity in subsets of mutant superoxide dismutases associated with familial amyotrophic lateral sclerosis. J Biol Chem.

[25] Liu R, Narla RK, Kurinov I, Li B, Uckun FM (1999). Increased hydroxyl radical production and apoptosis in PC12 neuron cells expressing the gain-of-function mutant G93A SOD1 gene. Radiat Res.

[26] Bogdanov M, Brown RH, Matson W, Smart R, Hayden D, O'Donnell H, Flint Beal M, Cudkowicz M (2000). Increased oxidative damage to DNA in ALS patients. Free Radic Biol Med.

[27] Ihara Y, Nobukuni K, Takata H, Hayabara T (2005). Oxidative stress and metal content in blood and cerebrospinal fluid of amyotrophic lateral sclerosis patients with and without a Cu Zn-superoxide dismutase mutation. Neurol Res.

[28] Aguirre N, Beal MF, Matson WR, Bogdanov MB (2005). Increased oxidative damage to DNA in an animal model of amyotrophic lateral sclerosis. Free Radic Res.

[29] Fang L, Teuchert M, Huber-Abel F, Schattauer D, Hendrich C, Dorst J, Zettlmeissel H, Wlaschek M, Scharffetter-Kochanek K, Kapfer T (2010). MMP-2 and MMP-9 are elevated in spinal cord and skin in a mouse model of ALS. J Neurol Sci.

[30] Cristini A, Ricci G, Britton S, Salimbeni S, Huang SN, Marinello J, Calsou P, Pommier Y, Favre G, Capranico G (2019). Dual processing of R-loops and topoisomerase I induces transcription-dependent DNA double-strand breaks. Cell Rep.

[31] Teng Y, Yadav T, Duan M, Tan J, Xiang Y, Gao B, Xu J, Liang Z, Liu Y, Nakajima S (2018). ROS-induced R loops trigger a transcription-coupled but BRCA1/2-independent homologous recombination pathway through CSB. Nat Commun.

[32] Reddy K, Schmidt MH, Geist JM, Thakkar NP, Panigrahi GB, Wang YH, Pearson CE (2014). Processing of double-R-loops in (CAG)·(CTG) and C9orf72 (GGGGCC)·(GGCCCC) repeats causes instability. Nucleic Acids Res.

[33] Rass U, Ahel I, West SC (2007). Defective DNA repair and neurodegenerative disease. Cell.

[34] Wang H, Dharmalingam P, Vasquez V, Mitra J, Boldogh I, Rao KS, Kent TA, Mitra S, Hegde ML (2017). Chronic oxidative damage together with genome repair deficiency in the neurons is a double whammy for neurodegeneration: Is damage response signaling a potential therapeutic target?. Mech Ageing Dev.

[35] Henriksen RA, Jenjaroenpun P, Sjostrom IB, Jensen KR, Prada-Luengo I, Wongsurawat T, Nookaew I, Regenberg B (2022). Circular DNA in the human germline and its association with recombination. Mol Cell.

[36] Kim H, Nguyen NP, Turner K, Wu S, Gujar AD, Luebeck J, Liu J, Deshpande V, Rajkumar U, Namburi S (2020). Extrachromosomal DNA is associated with oncogene amplification and poor outcome across multiple cancers. Nat Genet.

[37] Møller HD, Mohiyuddin M, Prada-Luengo I, Sailani MR, Halling JF, Plomgaard P, Maretty L, Hansen AJ, Snyder MP, Pilegaard H (2018). Circular DNA elements of chromosomal origin are common in healthy human somatic tissue. Nat Commun.

[38] Shibata Y, Kumar P, Layer R, Willcox S, Gagan JR, Griffith JD, Dutta A (2012). Extrachromosomal microDNAs and chromosomal microdeletions in normal tissues. Science.

[39] Singer MJ, Mesner LD, Friedman CL, Trask BJ, Hamlin JL (2000). Amplification of the human dihydrofolate reductase gene via double minutes is initiated by chromosome breaks. Proc Natl Acad Sci U S A.

[40] Paulsen T, Malapati P, Shibata Y, Wilson B, Eki R, Benamar M, Abbas T, Dutta A (2021). MicroDNA levels are dependent on MMEJ, repressed by c-NHEJ pathway, and stimulated by DNA damage. Nucleic Acids Res.

[41] Morton AR, Dogan-Artun N, Faber ZJ, MacLeod G, Bartels CF, Piazza MS, Allan KC, Mack SC, Wang X, Gimple RC (2019). Functional enhancers shape extrachromosomal oncogene amplifications. Cell.

[42] Turner KM, Deshpande V, Beyter D, Koga T, Rusert J, Lee C, Li B, Arden K, Ren B, Nathanson DA (2017). Extrachromosomal oncogene amplification drives tumour evolution and genetic heterogeneity. Nature.

[43] Koche RP, Rodriguez-Fos E, Helmsauer K, Burkert M, MacArthur IC, Maag J, Chamorro R, Munoz-Perez N, Puiggros M, Dorado Garcia H (2020). Extrachromosomal circular DNA drives oncogenic genome remodeling in neuroblastoma. Nat Genet.

[44] Wu S, Turner KM, Nguyen N, Raviram R, Erb M, Santini J, Luebeck J, Rajkumar U, Diao Y, Li B (2019). Circular ecDNA promotes accessible chromatin and high oncogene expression. Nature.

[45] Helmsauer K, Valieva ME, Ali S, Chamorro Gonzalez R, Schopflin R, Roefzaad C, Bei Y, Dorado Garcia H, Rodriguez-Fos E, Puiggros M (2020). Enhancer hijacking determines extrachromosomal circular MYCN amplicon architecture in neuroblastoma. Nat Commun.

[46] Paulsen T, Shibata Y, Kumar P, Dillon L, Dutta A (2019). Small extrachromosomal circular DNAs, microDNA, produce short regulatory RNAs that suppress gene expression independent of canonical promoters. Nucleic Acids Res.

[47] Zhu Y, Gujar AD, Wong CH, Tjong H, Ngan CY, Gong L, Chen YA, Kim H, Liu J, Li M (2021). Oncogenic extrachromosomal DNA functions as mobile enhancers to globally amplify chromosomal transcription. Cancer Cell.

[48] McCauley ME, O'Rourke JG, Yanez A, Markman JL, Ho R, Wang X, Chen S, Lall D, Jin M, Muhammad A (2020). C9orf72 in myeloid cells suppresses STING-induced inflammation. Nature.

[49] Sharma M, Rajendrarao S, Shahani N, Ramírez-Jarquín UN, Subramaniam S (2020). Cyclic GMP-AMP synthase promotes the inflammatory and autophagy responses in Huntington disease. Proc Natl Acad Sci U S A.

[50] Sliter DA, Martinez J, Hao L, Chen X, Sun N, Fischer TD, Burman JL, Li Y, Zhang Z, Narendra DP (2018). Parkin and PINK1 mitigate STING-induced inflammation. Nature.

[51] Yu CH, Davidson S, Harapas CR, Hilton JB, Mlodzianoski MJ, Laohamonthonkul P, Louis C, Low RRJ, Moecking J, De Nardo D (2020). TDP-43 Triggers mitochondrial DNA release via mPTP to activate cGAS/STING in ALS. Cell.

[52] Liu H, Zhang H, Wu X, Ma D, Wu J, Wang L, Jiang Y, Fei Y, Zhu C, Tan R (2018). Nuclear cGAS suppresses DNA repair and promotes tumorigenesis. Nature.

[53] Gurney ME, Pu H, Chiu AY, Dal Canto MC, Polchow CY, Alexander DD, Caliendo J, Hentati A, Kwon YW, Deng HX (1994). Motor neuron degeneration in mice that express a human Cu Zn superoxide dismutase mutation. Science.

[54] Rosen DR (1993). Mutations in Cu/Zn superoxide dismutase gene are associated with familial amyotrophic lateral sclerosis. Nature.

[55] Gerovska D, Araúzo-Bravo MJ (2023). Skeletal muscles of sedentary and physically active aged people have distinctive genic extrachromosomal circular DNA profiles. Int J Mol Sci.

[56] Gerovska D, Araúzo-Bravo MJ (2023). Systemic lupus erythematosus patients with DNASE1L3·deficiency have a distinctive and specific genic circular DNA profile in plasma. Cells.

[57] Kumar P, Kiran S, Saha S, Su Z, Paulsen T, Chatrath A, Shibata Y, Shibata E, Dutta A (2020). ATAC-seq identifies thousands of extrachromosomal circular DNA in cancer and cell lines. Sci Adv.

[58] Quinlan AR, Hall IM (2010). BEDTools: a flexible suite of utilities for comparing genomic features. Bioinformatics.

[59] Infante A, Gener B, Vázquez M, Olivares N, Arrieta A, Grau G, Llano I, Madero L, Bueno AM, Sagastizabal B (2021). Reiterative infusions of MSCs improve pediatric osteogenesis imperfecta eliciting a pro-osteogenic paracrine response: TERCELOI clinical trial. Clin Transl Med.

[60] Araúzo-Bravo MJ, Erichsen L, Ott P, Beermann A, Sheikh J, Gerovska D, Thimm C, Bendhack ML, Santourlidis S (2022). Consistent DNA hypomethylations in prostate cancer. Int J Mol Sci.

[61] Ding Z, Mangino M, Aviv A, Spector T, Durbin R (2014). Estimating telomere length from whole genome sequence data. Nucleic Acids Res.

[62] Ain Q, Schmeer C, Penndorf D, Fischer M, Bondeva T, Forster M, Haenold R, Witte OW, Kretz A (2018). Cell cycle-dependent and -independent telomere shortening accompanies murine brain aging. Aging.

[63] Buczak K, Kirkpatrick JM, Truckenmueller F, Santinha D, Ferreira L, Roessler S, Singer S, Beck M, Ori A (2020). Spatially resolved analysis of FFPE tissue proteomes by quantitative mass spectrometry. Nat Protoc.

[64] Storey JD (2002). A direct approach to false discovery rates. J Royal Stat Soc: Ser B (Stat Methodol).

[65] Khurana S, Kruhlak MJ, Kim J, Tran AD, Liu J, Nyswaner K, Shi L, Jailwala P, Sung MH, Hakim O, Oberdoerffer P (2014). A macrohistone variant links dynamic chromatin compaction to BRCA1-dependent genome maintenance. Cell Rep.

[66] Ruiz PD, Hamilton GA, Park JW, Gamble MJ (2019). MacroH2A1 regulation of poly(ADP-Ribose) synthesis and stability prevents necrosis and promotes DNA repair. Mol Cell Biol.

[67] Sakaue-Sawano A, Kurokawa H, Morimura T, Hanyu A, Hama H, Osawa H, Kashiwagi S, Fukami K, Miyata T, Miyoshi H (2008). Visualizing spatiotemporal dynamics of multicellular cell-cycle progression. Cell.

[68] Wang Y, Wang M, Djekidel MN, Chen H, Liu D, Alt FW, Zhang Y (2021). eccDNAs are apoptotic products with high innate immunostimulatory activity. Nature.

[69] Beshnova DA, Cherstvy AG, Vainshtein Y, Teif VB (2014). Regulation of the nucleosome repeat length in vivo by the DNA sequence, protein concentrations and long-range interactions. PLoS Comput Biol.

[70] Rouillard AD, Gundersen GW, Fernandez NF, Wang Z, Monteiro CD, McDermott MG, Ma'ayan A (2016). The harmonizome: a collection of processed datasets gathered to serve and mine knowledge about genes and proteins. Database.

[71] Sollis E, Mosaku A, Abid A, Buniello A, Cerezo M, Gil L, Groza T, Güneş O, Hall P, Hayhurst J (2023). The NHGRI-EBI GWAS catalog: knowledgebase and deposition resource. Nucleic Acids Res.

[72] Dillon LW, Kumar P, Shibata Y, Wang YH, Willcox S, Griffith JD, Pommier Y, Takeda S, Dutta A (2015). Production of extrachromosomal MicroDNAs is linked to mismatch repair pathways and transcriptional activity. Cell Rep.

[73] Wei PC, Chang AN, Kao J, Du Z, Meyers RM, Alt FW, Schwer B (2016). Long neural genes harbor recurrent DNA break clusters in neural stem/progenitor cells. Cell.

[74] Hewitt G, Jurk D, Marques FD, Correia-Melo C, Hardy T, Gackowska A, Anderson R, Taschuk M, Mann J, Passos JF (2012). Telomeres are favoured targets of a persistent DNA damage response in ageing and stress-induced senescence. Nat Commun.

[75] Parisi C, Napoli G, Amadio S, Spalloni A, Apolloni S, Longone P, Volonte C (2016). MicroRNA-125b regulates microglia activation and motor neuron death in ALS. Cell Death Differ.

[76] Nogami M, Ishikawa M, Doi A, Sano O, Sone T, Akiyama T, Aoki M, Nakanishi A, Ogi K, Yano M, Okano H (2021). Identification of hub molecules of FUS-ALS by bayesian gene regulatory network analysis of iPSC model: iBRN. Neurobiol Dis.

[77] Papoutsoglou P, Rodrigues-Junior DM, Moren A, Bergman A, Ponten F, Coulouarn C, Caja L, Heldin CH, Moustakas A (2021). The noncoding MIR100HG RNA enhances the autocrine function of transforming growth factor beta signaling. Oncogene.

[78] Rasheed Z, Rasheed N, Abdulmonem WA, Khan MI (2019). MicroRNA-125b-5p regulates IL-1beta induced inflammatory genes via targeting TRAF6-mediated MAPKs and NF-kappaB signaling in human osteoarthritic chondrocytes. Sci Rep.

[79] Retter I, Chevillard C, Scharfe M, Conrad A, Hafner M, Im TH, Ludewig M, Nordsiek G, Severitt S, Thies S (2007). Sequence and characterization of the Ig heavy chain constant and partial variable region of the mouse strain 129S1. J Immunol.

[80] Barnes IHA, Ibarra-Soria X, Fitzgerald S, Gonzalez JM, Davidson C, Hardy MP, Manthravadi D, Van Gerven L, Jorissen M, Zeng Z (2020). Expert curation of the human and mouse olfactory receptor gene repertoires identifies conserved coding regions split across two exons. BMC Genomics.

[81] van Blitterswijk M, van Es MA, Hennekam EA, Dooijes D, van Rheenen W, Medic J, Bourque PR, Schelhaas HJ, van der Kooi AJ, de Visser M (2012). Evidence for an oligogenic basis of amyotrophic lateral sclerosis. Hum Mol Genet.

[82] Decout A, Katz JD, Venkatraman S, Ablasser A (2021). The cGAS-STING pathway as a therapeutic target in inflammatory diseases. Nat Rev Immunol.

[83] Fryer AL, Abdullah A, Taylor JM, Crack PJ (2021). The complexity of the cGAS-STING pathway in CNS pathologies. Front Neurosci.

[84] Guerin MV, Regnier F, Feuillet V, Vimeux L, Weiss JM, Bismuth G, Altan-Bonnet G, Guilbert T, Thoreau M, Finisguerra V (2019). TGFbeta blocks IFNalpha/beta release and tumor rejection in spontaneous mammary tumors. Nat Commun.

[85] Di Zazzo E, De Rosa C, Abbondanza C, Moncharmont B (2013). PRDM proteins: molecular mechanisms in signal transduction and transcriptional regulation. Biology.

[86] Ferguson BW, Gao X, Zelazowski MJ, Lee J, Jeter CR, Abba MC, Aldaz CM (2013). The cancer gene WWOX behaves as an inhibitor of SMAD3 transcriptional activity via direct binding. BMC Cancer.

[87] Liu J, Huang Z, Chen HN, Qin S, Chen Y, Jiang J, Zhang Z, Luo M, Ye Q, Xie N (2021). ZNF37A promotes tumor metastasis through transcriptional control of THSD4/TGF-beta axis in colorectal cancer. Oncogene.

[88] Partridge EA, Le Roy C, Di Guglielmo GM, Pawling J, Cheung P, Granovsky M, Nabi IR, Wrana JL, Dennis JW (2004). Regulation of cytokine receptors by golgi n-glycan processing and endocytosis. Science.

[89] Wu K, Yang Y, Wang C, Davoli MA, D'Amico M, Li A, Cveklova K, Kozmik Z, Lisanti MP, Russell RG (2003). DACH1 inhibits transforming growth factor-beta signaling through binding Smad4. J Biol Chem.

[90] Bassani D, Pavan M, Federico S, Spalluto G, Sturlese M, Moro S (2022). The multifaceted role of GPCRs in amyotrophic lateral sclerosis: a new therapeutic perspective?. Int J Mol Sci.

[91] Gehrke N, Mertens C, Zillinger T, Wenzel J, Bald T, Zahn S, Tuting T, Hartmann G, Barchet W (2013). Oxidative damage of DNA confers resistance to cytosolic nuclease TREX1 degradation and potentiates STING-dependent immune sensing. Immunity.

[92] Honorat JA, Komorowski L, Josephs KA, Fechner K, St Louis EK, Hinson SR, Lederer S, Kumar N, Gadoth A, Lennon VA (2017). IgLON5 antibody: neurological accompaniments and outcomes in 20 patients. Neurol Neuroimmunol Neuroinflamm.

[93] Tao QQ, Wei Q, Song SJ, Yin XZ (2018). Motor neuron disease-like phenotype associated with anti-IgLON5 disease. CNS Neurosci Ther.

[94] Werner J, Jelcic I, Schwarz EI, Probst-Müller E, Nilsson J, Schwizer B, Bloch KE, Lutterotti A, Jung HH, Schreiner B (2021). Anti-IgLON5 disease: a new bulbar-onset motor neuron mimic syndrome. Neurol Neuroimmunol Neuroinflamm.

[95] Møller HD, Lin L, Xiang X, Petersen TS, Huang J, Yang L, Kjeldsen E, Jensen UB, Zhang X, Liu X (2018). CRISPR-C: circularization of genes and chromosome by CRISPR in human cells. Nucleic Acids Res.

[96] Feneberg E, Oeckl P, Steinacker P, Verde F, Barro C, Van Damme P, Gray E, Grosskreutz J, Jardel C, Kuhle J (2018). Multicenter evaluation of neurofilaments in early symptom onset amyotrophic lateral sclerosis. Neurology.

[97] Magen I, Yacovzada NS, Yanowski E, Coenen-Stass A, Grosskreutz J, Lu CH, Greensmith L, Malaspina A, Fratta P, Hornstein E (2021). Circulating miR-181 is a prognostic biomarker for amyotrophic lateral sclerosis. Nat Neurosci.

[98] Chi L, Gan L, Luo C, Lien L, Liu R (2007). Temporal response of neural progenitor cells to disease onset and progression in amyotrophic lateral sclerosis-like transgenic mice. Stem Cells Dev.

[99] Chi L, Ke Y, Luo C, Li B, Gozal D, Kalyanaraman B, Liu R (2006). Motor neuron degeneration promotes neural progenitor cell proliferation, migration, and neurogenesis in the spinal cords of amyotrophic lateral sclerosis mice. Stem Cells.

[100] Galán L, Gómez-Pinedo U, Guerrero A, García-Verdugo JM, Matías-Guiu J (2017). Amyotrophic lateral sclerosis modifies progenitor neural proliferation in adult classic neurogenic brain niches. BMC Neurol.

[101] Li R, Strykowski R, Meyer M, Mulcrone P, Krakora D, Suzuki M (2012). Male-specific differences in proliferation, neurogenesis, and sensitivity to oxidative stress in neural progenitor cells derived from a rat model of ALS. PLoS ONE.

[102] Wanchai V, Jenjaroenpun P, Leangapichart T, Arrey G, Burnham CM, Tümmler MC, Delgado-Calle J, Regenberg B, Nookaew I (2022). CReSIL: accurate identification of extrachromosomal circular DNA from long-read sequences. Brief Bioinform.

[103] Luebeck J, Ng AWT, Galipeau PC, Li X, Sanchez CA, Katz-Summercorn AC, Kim H, Jammula S, He Y, Lippman SM (2023). Extrachromosomal DNA in the cancerous transformation of Barrett’s oesophagus. Nature.

