# Supplementary material for: A distinct circular DNA profile intersects with proteome changes in the genotoxic stress-related hSOD1G93A model of ALS
Source: Cell Biosci. 2023 Sep 13;13:170. doi: 10.1186/s13578-023-01116-1 (PMC10498603; doi:10.1186/s13578-023-01116-1)
Supplement: Supplementary file 1 — Additional file 1: Figure S1. Oxidative stress-induced chromatin changes in ALS motor neurons. A Representative multichannel photomicrograph of the axially transected cervical myelon in a control (Ctrl) and hSOD1G93A (ALS) mutant. Rarified diseased SMI-32+ motor neurons (MNs; purple) of the anterior horn exhibited a higher frequency of mH2A1+ foci (green) in their nuclei (blue) than control MNs. The mH2A1+ chromatin foci are markers of DDR in response to oxidative DNA damage, with their location indicating the activation of PAR-dependent repair cascades. B Higher resolution single-channel images of an individual MN (dotted square) depicted in a control (Ctrl) and mutant (ALS) specimen. The nuclear Fucci red+ reporter signal assures the cell cycle-arrested, post-replicative state of SMI-32+ MNs. C Quantitative evaluation of mH2A1+ nuclear foci in control (Ctrl) and hSOD1G93A mutant (ALS) MNs. Indicated p-values were assessed by two-way ANOVA. **p < 0.01. For each group, n = 3. Scale bar in A, B: 20 μm. Channel illustration in A, B engaged pseudo-coloring. [file 13578_2023_1116_MOESM1_ESM.pdf]

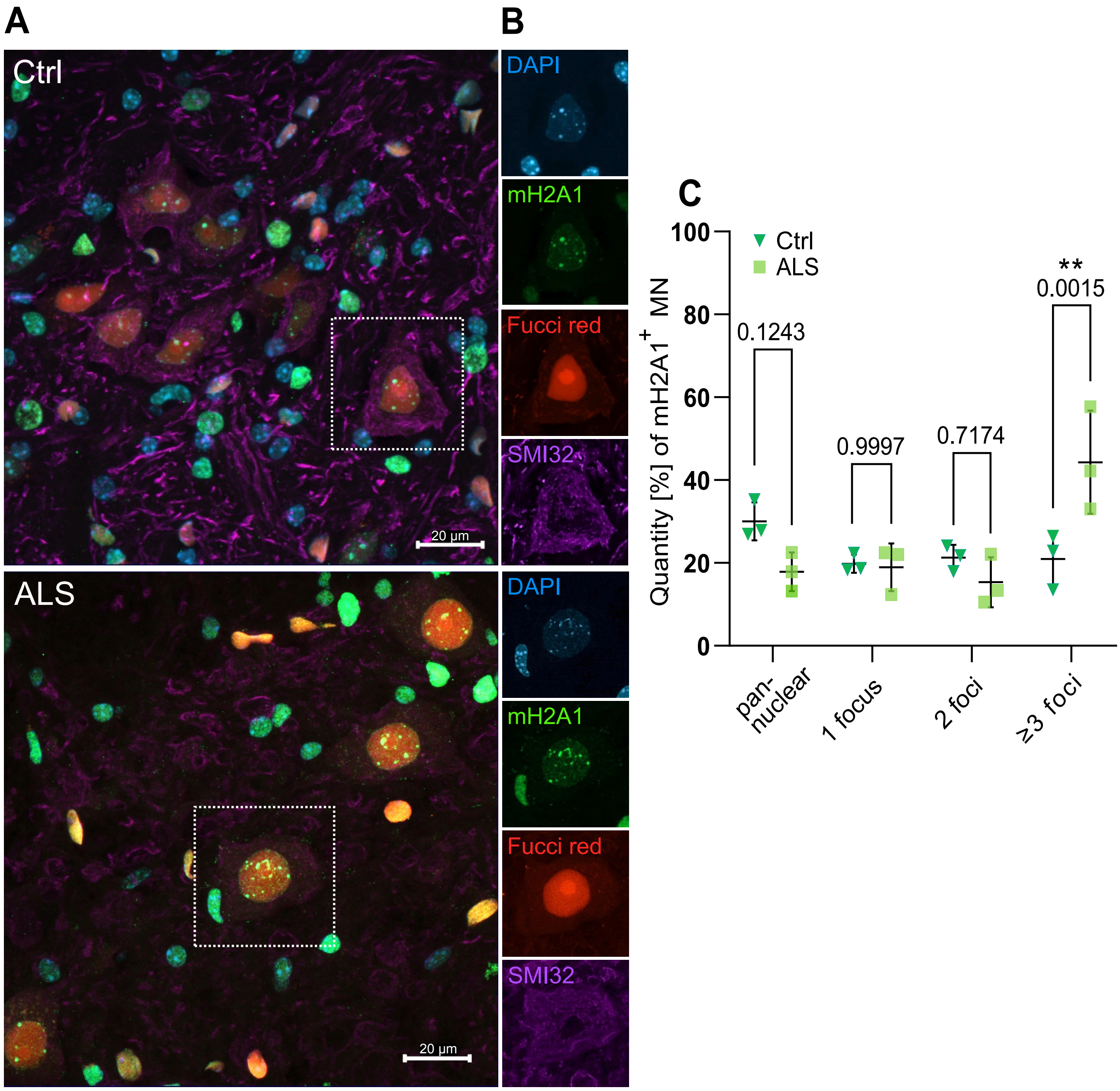

Additional file 1: Figure S1. Oxidative stress-induced chromatin changes in ALS motor neurons. A. Representative multichannel photomicrograph of the axially transected cervical myelon in a control (Ctrl) and *hSOD1*^{G93A} (ALS) mutant. Rarified diseased SMI-32^{+} motor neurons (MNs; purple) of the anterior horn exhibited a higher frequency of mH2A1^{+} foci (green) in their nuclei (blue) than control MNs. The mH2A1^{+} chromatin foci are markers of DDR in response to oxidative DNA damage, with their location indicating the activation of PAR-dependent repair cascades. **B.** Higher resolution single-channel images of an individual MN (dotted square) depicted in a control (Ctrl) and mutant (ALS) specimen. The nuclear Fucci red^{+} reporter signal assures the cell cycle-arrested, post-replicative state of SMI-32^{+} MNs. **C.** Quantitative evaluation of mH2A1^{+} nuclear foci in control (Ctrl) and *hSOD1*^{G93A} mutant (ALS) MNs. Indicated *p*-values were assessed by two-way ANOVA. **, *p* < 0.01. For each group, n = 3. Scale bar in A, B: 20 µm. Channel illustration in A, B engaged pseudo-coloring.
