# Supplementary material for: A distinct circular DNA profile intersects with proteome changes in the genotoxic stress-related hSOD1G93A model of ALS
Source: Cell Biosci. 2023 Sep 13;13:170. doi: 10.1186/s13578-023-01116-1 (PMC10498603; doi:10.1186/s13578-023-01116-1)
Supplement: Supplementary file 2 — Additional file 2: Figure S2. Sample-individual distributions of length-sorted eccDNAs, related to Figure 2. The histograms indicate the eccDNA distributions up to a size of 104 bp. Represented are the results for (A) control (C1-10; blue) and (B) ALS (A1-9; red) samples after removal of mt-DNA sequences and after merging and exclusion of eccDNAs with less than 2 split reads. [file 13578_2023_1116_MOESM2_ESM.pdf]

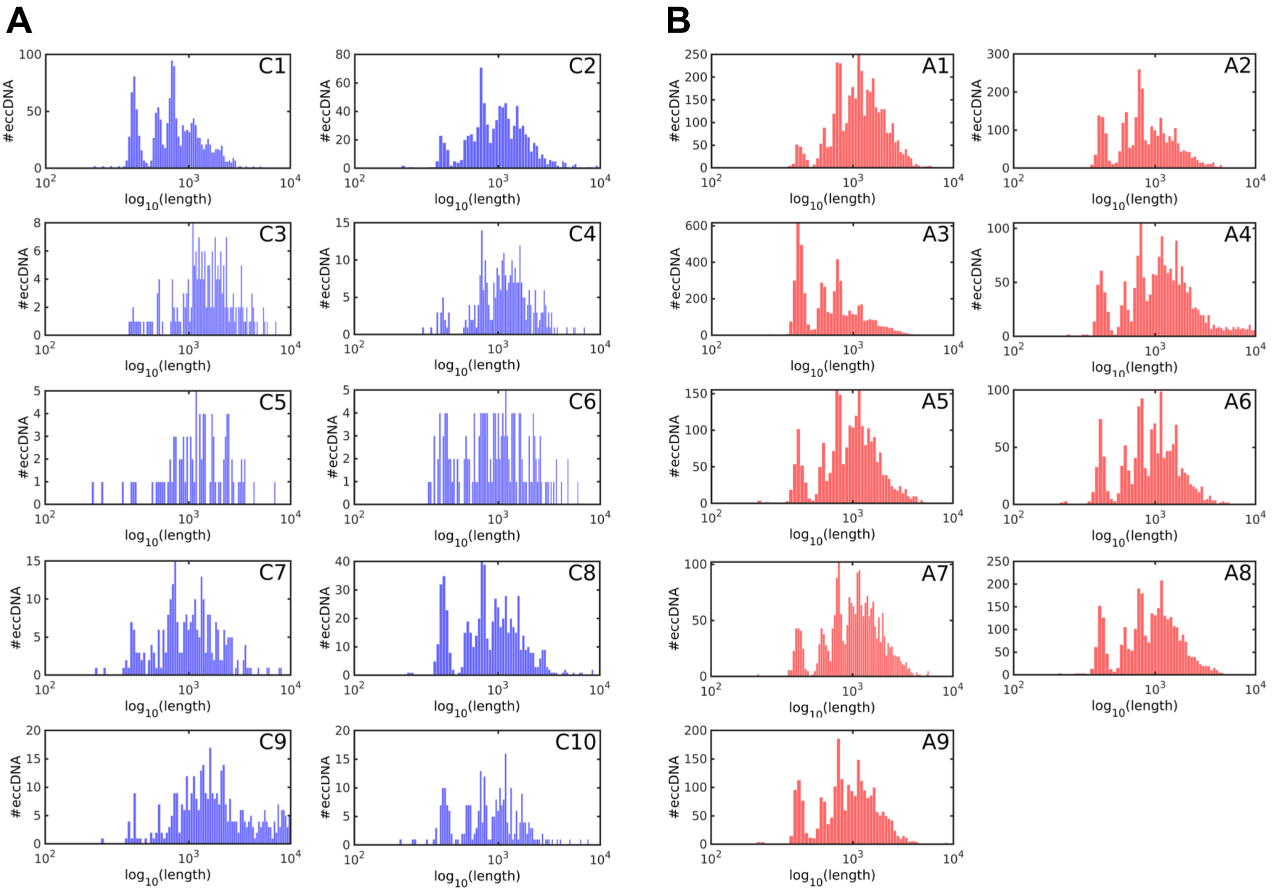

Additional file 2: Figure S2. Sample-individual distributions of length-sorted eccDNAs, related to Figure 2. The histograms indicate the eccDNA distributions up to a size of 10^4 bp. Represented are the results for (A) control (C1-10; blue) and (B) ALS (A1-9; red) samples after removal of mt-DNA sequences and after merging and exclusion of eccDNAs with less than 2 split reads.
