# Supplementary material for: A distinct circular DNA profile intersects with proteome changes in the genotoxic stress-related hSOD1G93A model of ALS
Source: Cell Biosci. 2023 Sep 13;13:170. doi: 10.1186/s13578-023-01116-1 (PMC10498603; doi:10.1186/s13578-023-01116-1)
Supplement: Supplementary file 3 — Additional file 3: Figure S3. Violin plots of the length distribution of eccDNAs < 105 bp for all murine chromosomes and samples. Represented are the results after removal of mt-DNA sequences and after merging and exclusion of eccDNAs with less than 2 split reads. For (A) control (C1-10) and (B) ALS (A1-9) samples, the mean and median eccDNA read lengths cumulatively assigned to a certain chromosome are displayed as red crosses and green squares, respectively. Data points are overexposed in light blue. For A, n = 10 control and B, n = 9 ALS samples. [file 13578_2023_1116_MOESM3_ESM.pdf]

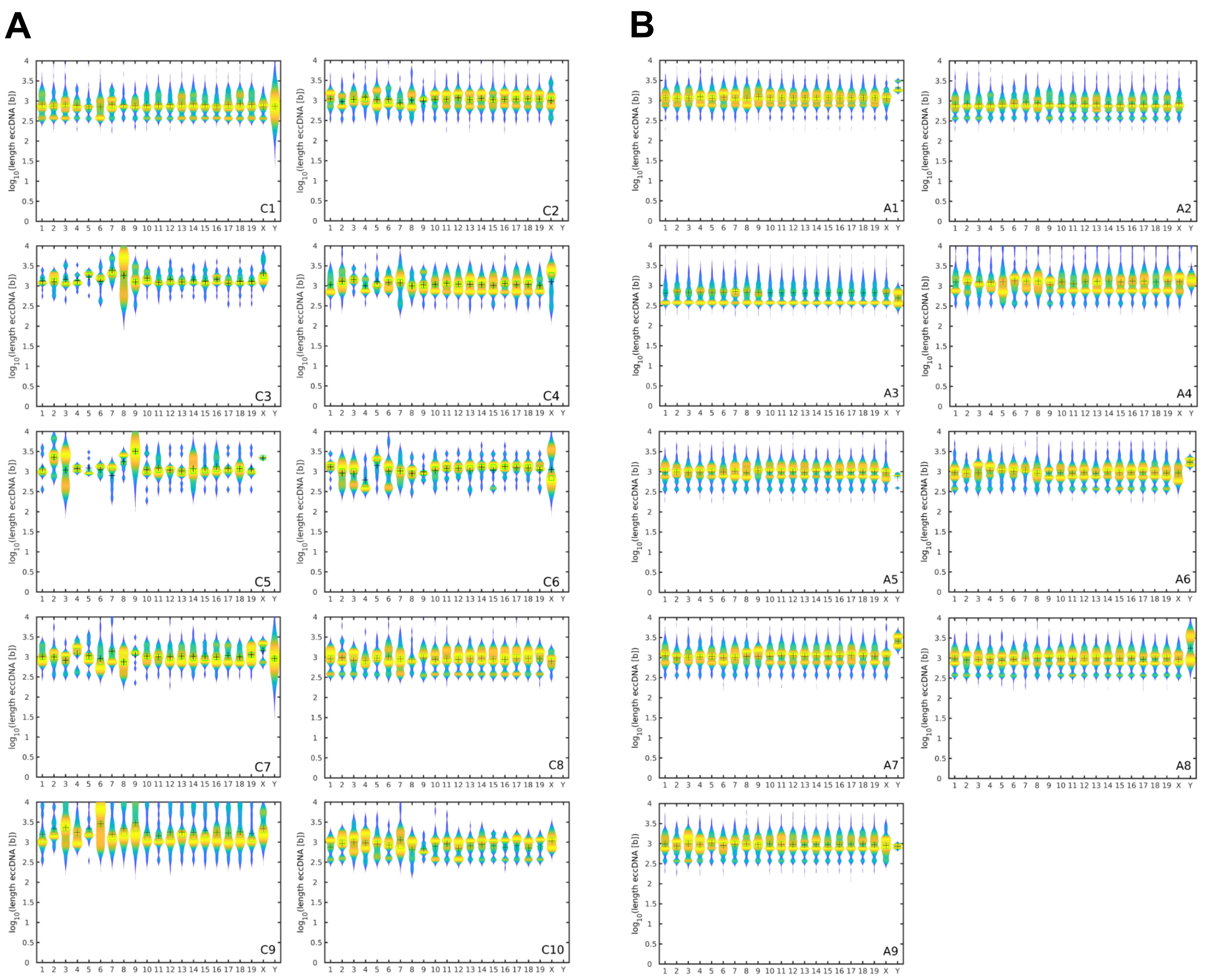

Additional file 3: Figure S3. Violin plots of the length distribution of eccDNAs < 10^5 bp for all murine chromosomes and samples. Represented are the results after removal of mt-DNA sequences and after merging and exclusion of eccDNAs with less than 2 split reads. For (A) control (C1-10) and (B) ALS (A1-9) samples, the mean and median eccDNA read lengths cumulatively assigned to a certain chromosome are displayed as red crosses and green squares, respectively. Data points are overexposed in light blue. For A, n = 10 control and B, n = 9 ALS samples.
