# Supplementary material for: A distinct circular DNA profile intersects with proteome changes in the genotoxic stress-related hSOD1G93A model of ALS
Source: Cell Biosci. 2023 Sep 13;13:170. doi: 10.1186/s13578-023-01116-1 (PMC10498603; doi:10.1186/s13578-023-01116-1)
Supplement: Supplementary file 4 — Additional file 4: Figure S4. Heatmap of the top 1000 most variable eccDNAs produced per gene (PpGCs). The color bar on the top codifies the PpGCs in log2 scale. Higher values of PpGCs correspond to redder color. C, control; A, ALS group. [file 13578_2023_1116_MOESM4_ESM.pdf]

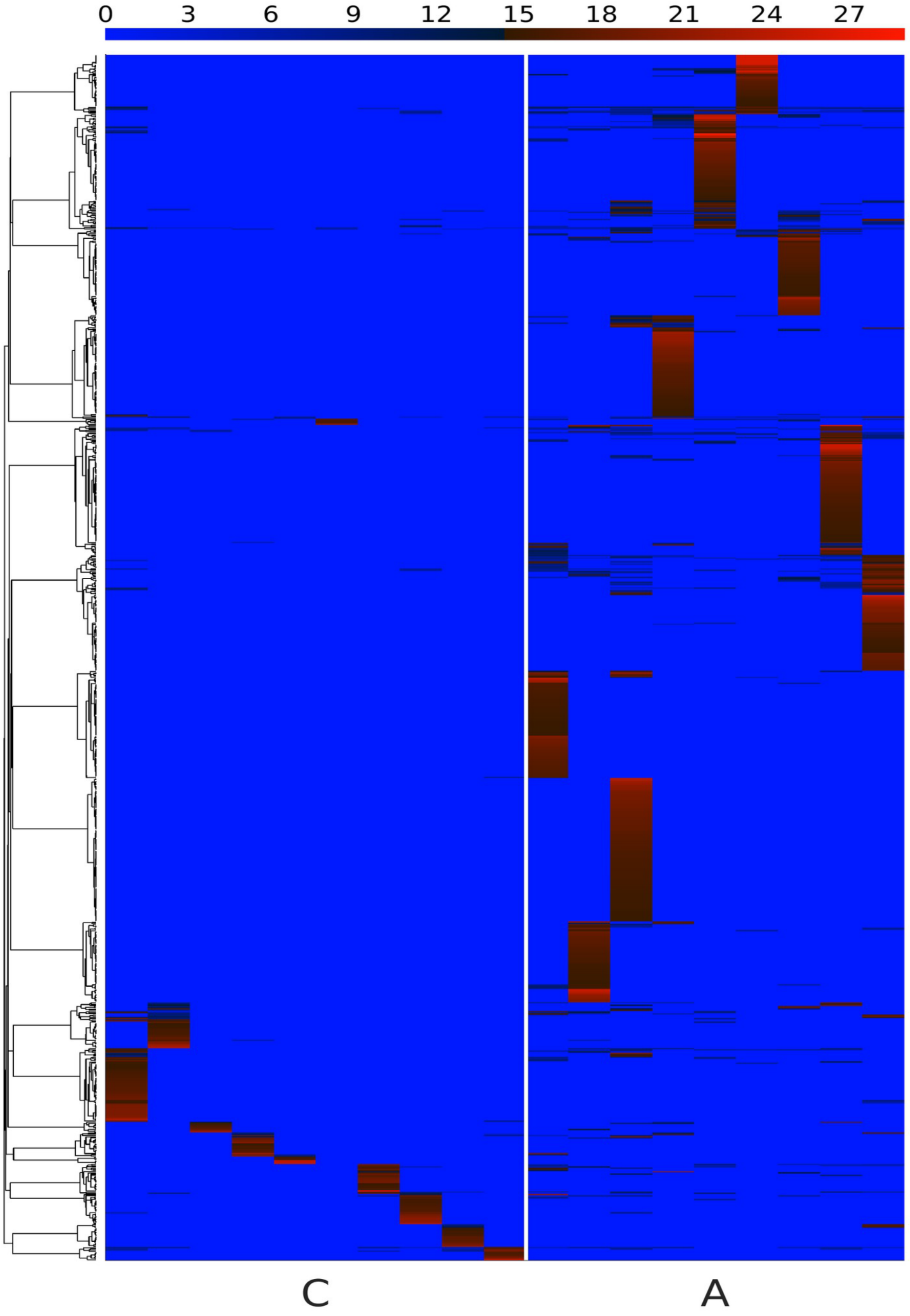

Additional file 4: Figure S4. Heatmap of the top 1000 most variable eccDNAs produced per gene (PpGCs). The color bar on the top codifies the PpGCs in $\log_2$ scale. Higher values of PpGCs correspond to redder color. C, control; A, ALS group.
