# Supplementary material for: A distinct circular DNA profile intersects with proteome changes in the genotoxic stress-related hSOD1G93A model of ALS
Source: Cell Biosci. 2023 Sep 13;13:170. doi: 10.1186/s13578-023-01116-1 (PMC10498603; doi:10.1186/s13578-023-01116-1)
Supplement: Supplementary file 5 — Additional file 5 Figure S5. Topology of the 225 up-DPpGCs in the ALS versus control genome, related to Figure 4. Each linear circular DNA panel aligns the position (black rectangles) of the unique DPpGCs to their originating hotspot genes. Each horizontal line represents the length of a gene; the red lines correspond to ALS (A), the blue lines to control (C) samples. [file 13578_2023_1116_MOESM5_ESM.pdf]

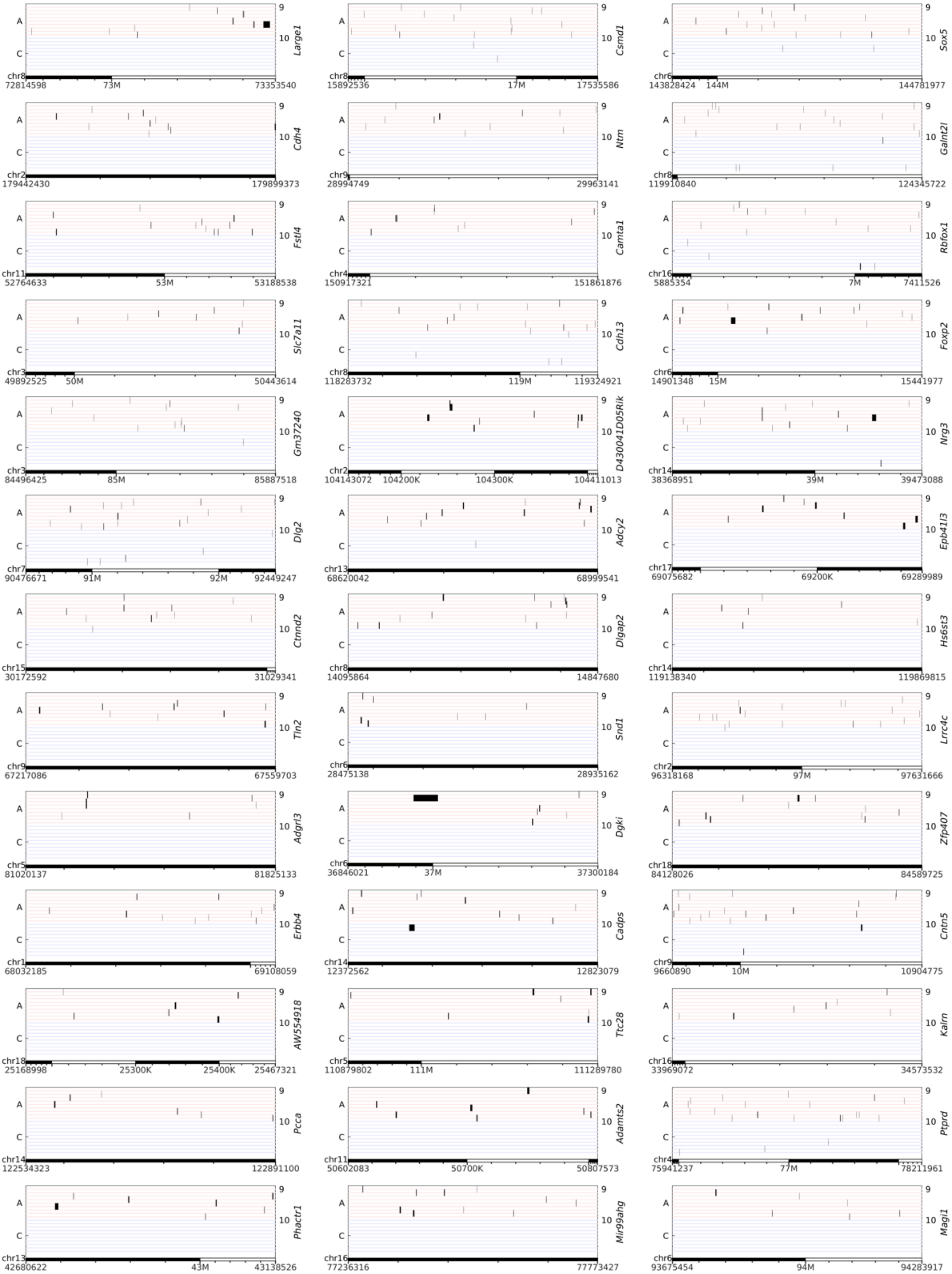
A
C
9
10
Large1
chr8
72814598
73M
73353540
Csmd1
chr8
15892536
17M
17535586
Sox5
chr6
143828424
144M
144781977
Cdh4
chr2
179442430
179899373
Ntm
chr9
28994749
29963141
Galnt2l
chr8
119910840
124345722
Fstl4
chr11
52764633
53M
53188538
Camta1
chr4
150917321
151861876
Rbfox1
chr16
5885354
7M
7411526
Slc7a11
chr3
49892525
50M
50443614
Cdh13
chr8
118283732
119M
119324921
Foxp2
chr6
14901348
15M
15441977
Gm37240
chr3
84496425
85M
85887518
D430041D05Rik
chr2
104143072
104200K
104300K
104411013
Nrg3
chr14
38368951
39M
39473088
Dlg2
chr7
90476671
91M
92M
92449247
Adcy2
chr13
68620042
68999541
Epb41l3
chr17
69075682
69200K
69289989
Ctnnd2
chr15
30172592
31029341
Dlgap2
chr8
14095864
14847680
Hs6st3
chr14
119138340
119869815
Tln2
chr9
67217086
67559703
Snd1
chr6
28475138
28935162
Lrrc4c
chr2
96318168
97M
97631666
Adgrl3
chr5
81020137
81825133
Dgki
chr6
36846021
37M
37300184
Zfp407
chr18
84128026
84589725
Erbb4
chr1
68032185
69108059
Cadps
chr14
12372562
12823079
Cntn5
chr9
9660890
10M
10904775
AW554918
chr18
25168998
25300K
25400K
25467321
Ttc28
chr5
110879802
111M
111289780
Kalrn
chr16
33969072
34573532
Pcca
chr14
122534323
122891100
Adamts2
chr11
50602083
50700K
50807573
Ptprd
chr4
75941237
77M
78211961
Phactr1
chr13
42680622
43M
43138526
Mir99ahg
chr16
77236316
77773427
Magi1
chr6
93675454
94M
94283917

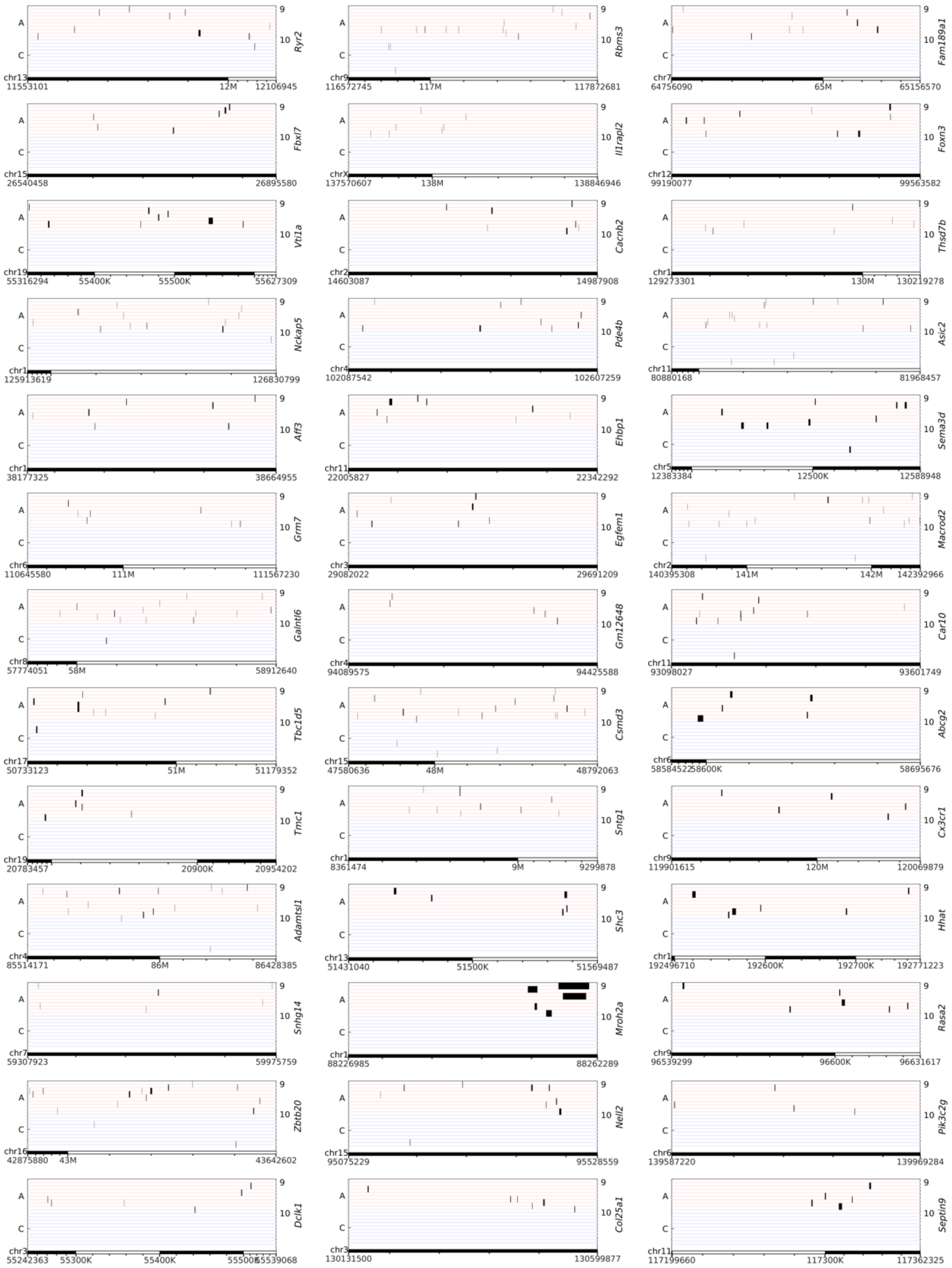
A
C
9
10
Ryr2
chr13
11553101
12M
12106945
Rbms3
chr9
116572745
117M
117872681
Fam189a1
chr7
64756090
65M
65156570
Fbxl7
chr15
26540458
26895580
Il1rapl2
chrX
137570607
138M
138846946
Foxn3
chr12
99190077
99563582
Vti1a
chr19
55316294
55400K
55500K
55627309
Cacnb2
chr2
14603087
14987908
Thsd7b
chr1
129273301
130M
130219278
Nckap5
chr1
125913619
126830799
Pde4b
chr4
102087542
102607259
Asic2
chr11
80880168
81968457
Aff3
chr1
38177325
38664955
Ehbp1
chr11
22005827
22342292
Sema3d
chr5
12383384
12500K
12588948
Grm7
chr6
110645580
111M
111567230
Egfem1
chr3
29082022
29691209
Macrod2
chr2
140395308
141M
142M
142392966
Galntl6
chr8
57774051
58M
58912640
Gm12648
chr4
94089575
94425588
Car10
chr11
93098027
93601749
Tbc1d5
chr17
50733123
51M
51179352
Csmd3
chr15
47580636
48M
48792063
Abcg2
chr6
58584522
58600K
58695676
Tmc1
chr19
20783457
20900K
20954202
Sntg1
chr1
8361474
9M
9299878
Cx3cr1
chr9
119901615
120M
120069879
Adamtsl1
chr4
85514171
86M
86428385
Shc3
chr13
51431040
51500K
51569487
Hhat
chr1
192496710
192600K
192700K
192771223
Snhg14
chr7
59307923
59975759
Mroh2a
chr1
88226985
88262289
Rasa2
chr9
96539299
96600K
96631617
Zbtb20
chr16
42875880
43M
43642602
Nell2
chr15
95075229
95528559
Pik3c2g
chr6
139587220
139969284
Dclk1
chr3
55242363
55300K
55400K
55500K
55539068
Col25a1
chr3
130131500
130599877
Septin9
chr11
117199660
117300K
117362325

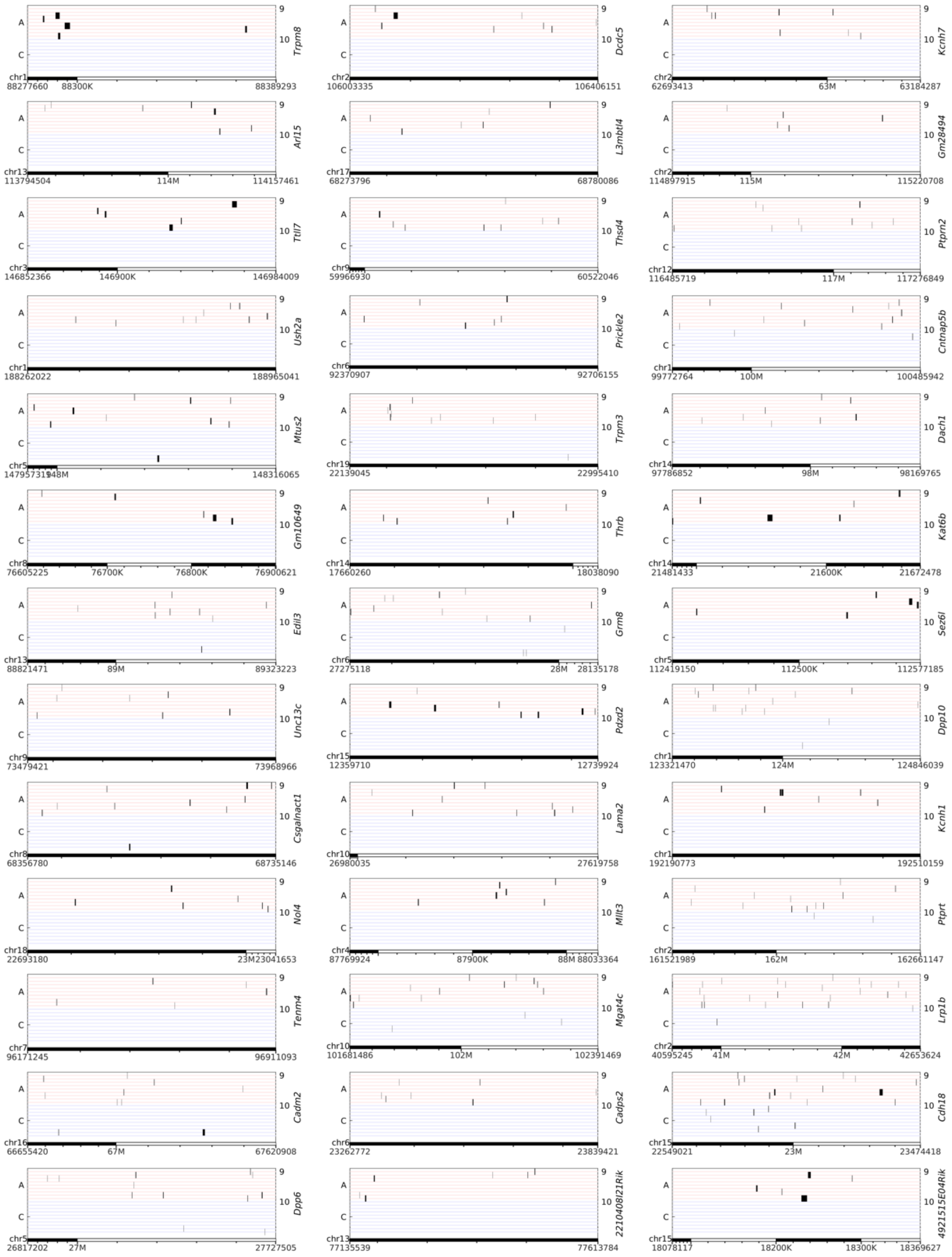
A C 9 10
Trpm8 chr1 88277660 88300K 88389293
Dcdc5 chr2 106003335 106406151
Kcnh7 chr2 62693413 63M 63184287
Arl15 chr13 113794504 114M 114157461
L3mbtl4 chr17 68273796 68780086
Gm28494 chr2 114897915 115M 115220708
Ttll7 chr3 146852366 146900K 146984009
Thsd4 chr9 59966930 60522046
Ptprn2 chr12 116485719 117M 117276849
Ush2a chr1 188262022 188965041
Prickle2 chr6 92370907 92706155
Cntnap5b chr1 99772764 100M 100485942
Mtus2 chr5 147957319 148M 148316065
Trpm3 chr19 22139045 22995410
Dach1 chr14 97786852 98M 98169765
Gm10649 chr8 76605225 76700K 76800K 76900621
Thrb chr14 17660260 18038090
Kat6b chr14 21481433 21600K 21672478
Edil3 chr13 88821471 89M 89323223
Grm8 chr6 27275118 28M 28135178
Sez6l chr5 112419150 112500K 112577185
Unc13c chr9 73479421 73968966
Pdzd2 chr15 12359710 12739924
Dpp10 chr1 123321470 124M 124846039
Csgalnact1 chr8 68356780 68735146
Lama2 chr10 26980035 27619758
Kcnh1 chr1 192190773 192510159
Nol4 chr18 22693180 23M 23041653
Mllt3 chr4 87769924 87900K 88M 88033364
Ptprt chr2 161521989 162M 162661147
Tenm4 chr7 96171245 96911093
Mgat4c chr10 101681486 102M 102391469
Lrp1b chr2 40595245 41M 42M 42653624
Cadm2 chr16 66655420 67M 67620908
Cadps2 chr6 23262772 23839421
Cdh18 chr15 22549021 23M 23474418
Dpp6 chr5 26817202 27M 27727505
2210408I21Rik chr13 77135539 77613784
4921515E04Rik chr15 18078117 18200K 18300K 18369627

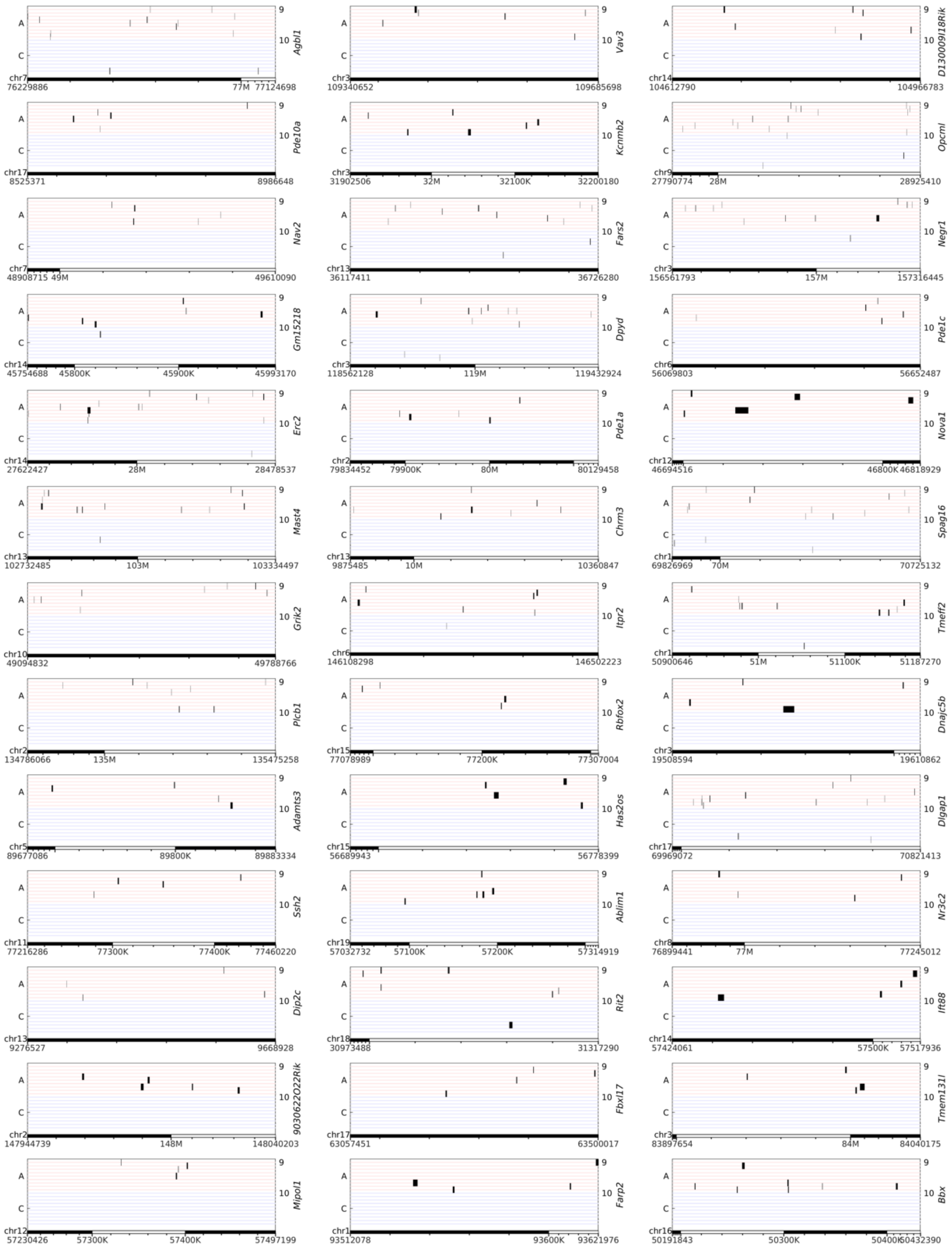

A
C
9
10
Agbl1
chr7 76229886 77M 77124698
Vav3
chr3 109340652 109685698
D130009I18Rik
chr14 104612790 104966783
Pde10a
chr17 8525371 8986648
Kcnmb2
chr3 31902506 32M 32100K 32200180
Opcml
chr9 27790774 28M 28925410
Nav2
chr7 48908715 49M 49610090
Fars2
chr13 36117411 36726280
Negr1
chr3 156561793 157M 157316445
Gm15218
chr14 45754688 45800K 45900K 45993170
Dpyd
chr3 118562128 119M 119432924
Pde1c
chr6 56069803 56652487
Erc2
chr14 27622427 28M 28478537
Pde1a
chr2 79834452 79900K 80M 80129458
Nova1
chr12 46694516 46800K 46818929
Mast4
chr13 102732485 103M 103334497
Chrm3
chr13 9875485 10M 10360847
Spag16
chr1 69826969 70M 70725132
Grik2
chr10 49094832 49788766
Itpr2
chr6 146108298 146502223
Tmeff2
chr1 50900646 51M 51100K 51187270
Plcb1
chr2 134786066 135M 135475258
Rbfox2
chr15 77078989 77200K 77307004
Dnajc5b
chr3 19508594 19610862
Adamts3
chr5 89677086 89800K 89883334
Has2os
chr15 56689943 56778399
Dlgap1
chr17 69969072 70821413
Ssh2
chr11 77216286 77300K 77400K 77460220
Ablim1
chr19 57032732 57100K 57200K 57314919
Nr3c2
chr8 76899441 77M 77245012
Dip2c
chr13 9276527 9668928
Rit2
chr18 30973488 31317290
Ift88
chr14 57424061 57500K 57517936
9030622O22Rik
chr2 147944739 148M 148040203
Fbxl17
chr17 63057451 63500017
Tmem131l
chr3 83897654 84M 84040175
Mipol1
chr12 57230426 57300K 57400K 57497199
Farp2
chr1 93512078 93600K 93621976
Bbx
chr16 50191843 50300K 50400K 50432390

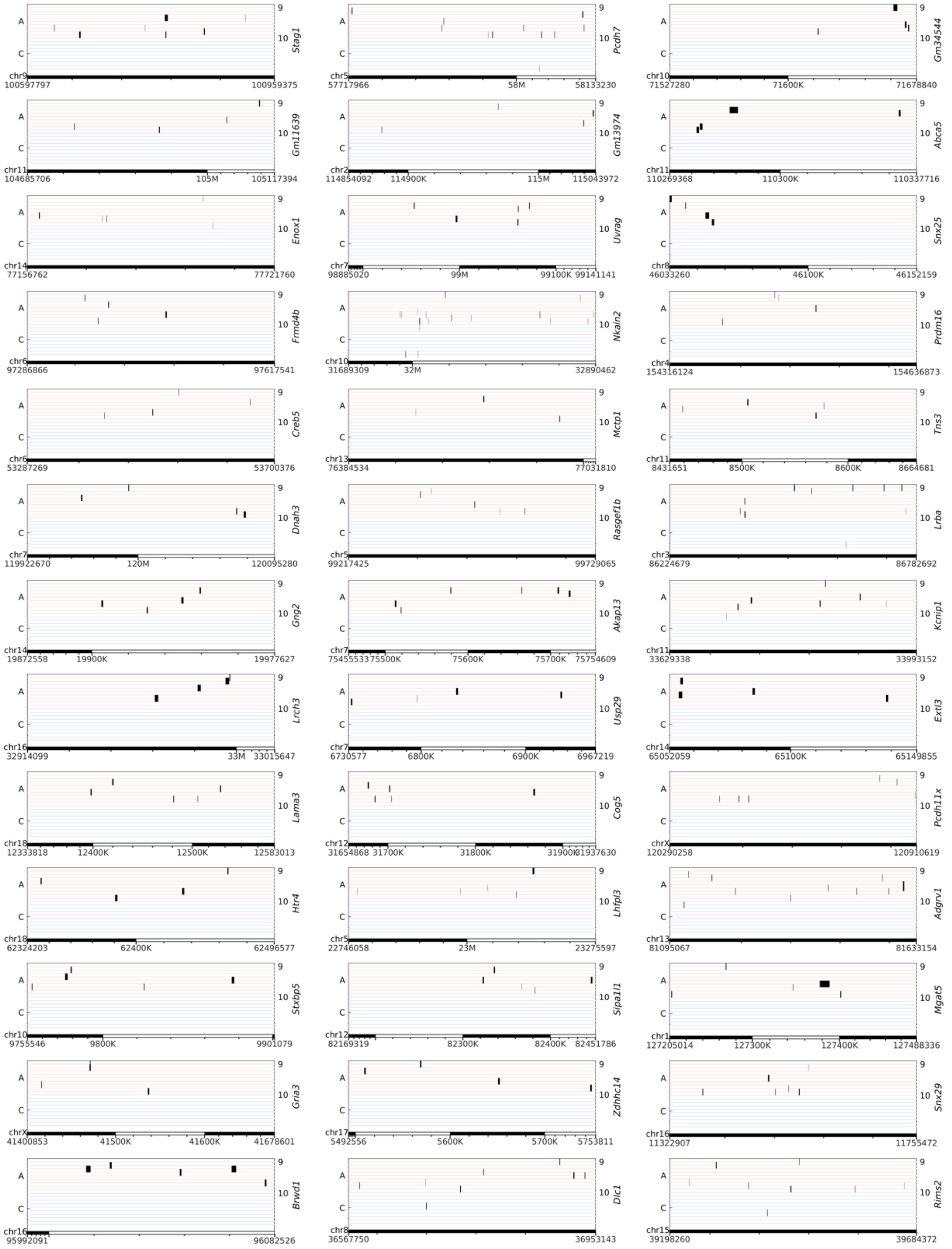

Additional file 5: Figure S5. Topology of the 225 up-DPpGCs in the ALS versus control genome, related to Figure 4. Each linear circular DNA panel aligns the position (black rectangles) of the unique DPpGCs to their originating hotspot genes. Each horizontal line represents the length of a gene; the red lines correspond to ALS (A), the blue lines to control (C) samples.
