# Supplementary material for: A distinct circular DNA profile intersects with proteome changes in the genotoxic stress-related hSOD1G93A model of ALS
Source: Cell Biosci. 2023 Sep 13;13:170. doi: 10.1186/s13578-023-01116-1 (PMC10498603; doi:10.1186/s13578-023-01116-1)
Supplement: Supplementary file 6 — Additional file 6: Figure S6. Whole coding genes on eccDNAs. A Chromosomal landscaping of the genomic loci giving rise to whole coding genes on eccDNAs specific for ALS conditions. The genes in potential loci clusters are marked in magenta. B List of whole coding genes on eccDNAs that are specific for control samples (blue) and ALS samples (red) or found in both groups (green). [file 13578_2023_1116_MOESM6_ESM.pdf]

A

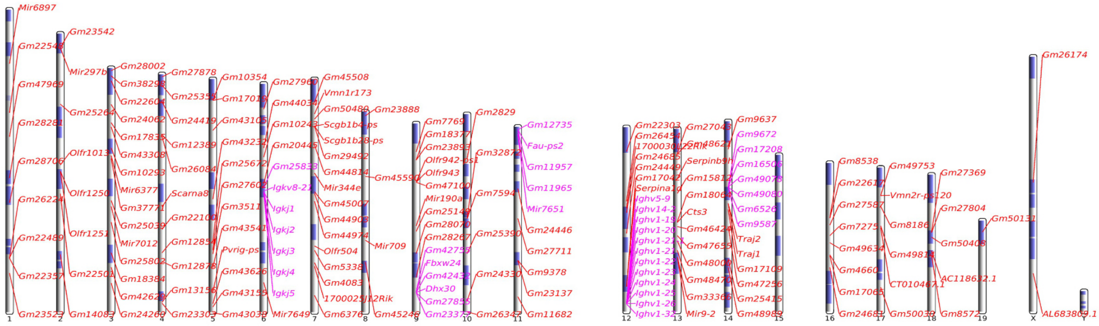

B *1700025J12Rik chr7 1700030L22Rik chr12 4930540E01Rik chr9 AC118632.1 chr18 AL683809.1 chrX CT010467.1 chr17 Cts3 chr13 Dhx30 chr9 Fau-ps2 chr11 Fbxw24 chr9 Ftl1-ps2 chr10 Gm10243 chr6 Gm10293 chr3 Gm10343 chr16 Gm10354 chr5 Gm11682 chr11 Gm11957 chr11 Gm11965 chr11 Gm12389 chr4 Gm12735 chr11 Gm12815 chr4 Gm12854 chr4 Gm12878 chr4 Gm13156 chr4 Gm14083 chr2 Gm15628 chr14 Gm15812 chr13 Gm15982 chr5 Gm16506 chr14 Gm17019 chr5 Gm17042 chr12 Gm17065 chr16 Gm17109 chr14 Gm17208 chr14 Gm17826 chr7 Gm17835 chr3 Gm18064 chr13 Gm18377 chr9 Gm18384 chr3 Gm18387 chr12 Gm18878 chr13 Gm19185 chr3 Gm20445 chr6 Gm22100 chr4 Gm22257 chrX Gm22303 chr12 Gm22304 chr9 Gm22357 chr1 Gm22489 chr1 Gm22498 chr14 Gm22501 chr2 Gm22548 chr1 Gm22604 chr3 Gm22617 chr16 Gm23137 chr11 Gm23303 chr4 Gm23377 chr9 Gm23523 chr1 Gm23542 chr2 Gm23888 chr8 Gm23893 chr9 Gm23972 chr13 Gm24014 chr1 Gm24062 chr3 Gm24195 chr13 Gm24260 chr3 Gm24330 chr10 Gm24419 chr4 Gm24446 chr11 Gm24449 chr12 Gm24681 chr16 Gm24685 chr12 Gm25021 chr6 Gm25039 chr3 Gm25149 chr9 Gm25261 chr4 Gm25264 chr2 Gm25355 chr4 Gm25390 chr10 Gm25415 chr14 Gm25672 chr5 Gm25802 chr3 Gm25833 chr6 Gm26084 chr4 Gm26174 chrX Gm26224 chr1 Gm26347 chr10 Gm26445 chr10 Gm26454 chr12 Gm27043 chr13 Gm27369 chr18 Gm27587 chr16 Gm27602 chr5 Gm27711 chr11 Gm27804 chr18 Gm27855 chr9 Gm27878 chr4 Gm27960 chr6 Gm28002 chr3 Gm28070 chr9 Gm28228 chr19 Gm28267 chr9 Gm28281 chr1 Gm2829 chr10 Gm28706 chr1 Gm29492 chr7 Gm32222 chr6 Gm32872 chr10 Gm33366 chr13 Gm3511 chr5 Gm37527 chr6 Gm37771 chr3 Gm38051 chr1 Gm38298 chr3 Gm38359 chr1 Gm4083 chr7 Gm42432 chr9 Gm42629 chr3 Gm42755 chr9 Gm43038 chr5 Gm43105 chr5 Gm43155 chr5 Gm43232 chr5 Gm43308 chr3 Gm43445 chr3 Gm43541 chr5 Gm43626 chr5 Gm44034 chr6 Gm44063 chr2 Gm44340 chr14 Gm44814 chr7 Gm44903 chr7 Gm44974 chr7 Gm45007 chr7 Gm45246 chr8 Gm45508 chr7 Gm45590 chr8 Gm46424 chr13 Gm4660 chr16 Gm47100 chr9 Gm47256 chr14 Gm47655 chr13 Gm47969 chr1 Gm48000 chr13 Gm48470 chr13 Gm48621 chr13 Gm48989 chr14 Gm49078 chr14 Gm49080 chr14 Gm49485 chr15 Gm49612 chr16 Gm49634 chr16 Gm49753 chr17 Gm49814 chr17 Gm50030 chr17 Gm50131 chr19 Gm50228 chr18 Gm50408 chr18 Gm50480 chr7 Gm5338 chr7 Gm6376 chr7 Gm6526 chr14 Gm7275 chr16 Gm7594 chr10 Gm7769 chr9 Gm7984 chr8 Gm8186 chr17 Gm8538 chr16 Gm8572 chr18 Gm9298 chr14 Gm9378 chr11 Gm9587 chr14 Gm9637 chr14 Gm9672 chr14 Ighv1-19 chr12 Ighv1-20 chr12 Ighv1-21 chr12 Ighv1-21-1 chr12 Ighv1-22 chr12 Ighv1-23 chr12 Ighv1-24 chr12 Ighv1-25 chr12 Ighv1-26 chr12 Ighv1-32 chr12 Ighv1-48 chr12 Ighv14-2 chr12 Ighv5-9 chr12 Igkj1 chr6 Igkj2 chr6 Igkj3 chr6 Igkj4 chr6 Igkj5 chr6 Igkv8-21 chr6 Mir190a chr9 Mir297b chr2 Mir3073a chr12 Mir3073b chr12 Mir344e chr7 Mir6377 chr3 Mir6897 chr1 Mir7012 chr3 Mir7014 chr3 Mir709 chr8 Mir7649 chr6 Mir7651 chr11 Mir9-2 chr13 Olfr1013 chr2 Olfr1052 chr2 Olfr1250 chr2 Olfr1251 chr2 Olfr504 chr7 Olfr942-ps1 chr9 Olfr943 chr9 Pvrig-ps chr5 Rpl26-ps3 chr12 Scarna8 chr4 Scgb1b28-ps chr7 Scgb1b4-ps chr7 Serpina1d chr12 Serpinb9h chr13 Tas2r113 chr6 Traj1 chr14 Traj2 chr14 Traj40 chr14 Traj59 chr14 Traj60 chr14 Trbj2-1 chr6 Trbj2-2 chr6 Trbj2-3 chr6 Trbj2-4 chr6 Trbj2-5 chr6 Trbj2-6 chr6 Trbv11 chr6 Vmn1r173 chr7 Vmn2r-ps120 chr17*.

Additional file 6: Figure S6. Whole coding genes on eccDNAs. A. Chromosomal landscaping of the genomic *loci* giving rise to whole coding genes on eccDNAs specific for ALS conditions. The genes in potential *loci* clusters are marked in magenta. **B.** List of whole coding genes on eccDNAs that are specific for control samples (blue) and ALS samples (red) or found in both groups (green).
