# Supplementary material for: A distinct circular DNA profile intersects with proteome changes in the genotoxic stress-related hSOD1G93A model of ALS
Source: Cell Biosci. 2023 Sep 13;13:170. doi: 10.1186/s13578-023-01116-1 (PMC10498603; doi:10.1186/s13578-023-01116-1)
Supplement: Supplementary file 7 — Additional file 7: Table S1. Main intersections of the 225 up-DPpGCs with DEPs and with ALS-associated loci annotated in GWAS databases, according to the Venn diagram in Fig. 5A. [file 13578_2023_1116_MOESM7_ESM.pdf]

**Additional file 7: Table S1.** Main intersections of the 225 up-DPpGCs with DEPs and with ALS-associated *loci* annotated in GWAS databases, according to the Venn diagram in Fig. 5A.

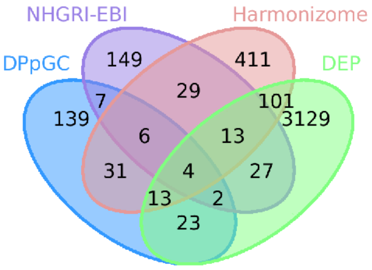


**Sets of comparisons:**

- **Set A:** DPpGC
- **Set B:** NHGRI-EBI
- **Set C:** Harmonizome
- **Set D:** DEPs

**Number of elements in Set A: 225**

Large1, Csmd1, Sox5, Cdh4, Ntm, Galnt2l, Fstl4, Camta1, Rbfox1, Slc7a11, Cdh13, Foxp2, Gm37240, D430041d05rik, Nrg3, Dlg2, Adcy2, Epb41l3, Ctnnd2, Dlgap2, Hs6st3, Tln2, Snd1, Lrrc4c, Adgrl3, Dgki, Zfp407, Erbb4, Cadps, Cntn5, Aw554918, Ttc28, Kalrn, Pcca, Adamts2, Ptprd, Phactr1, Mir99ahg, Magi1, Ryr2, Rbms3, Fam189a1, Fbxl7, Il1rapl2, Foxn3, Vti1a, Cacnb2, Thsd7b, Nckap5, Pde4b, Asic2, Aff3, Ehbp1, Sema3d, Grm7, Egfem1, Macrod2, Galntl6, Gm12648, Car10, Tbc1d5, Csmd3, Abcg2, Tmc1, Sntg1, Cx3cr1, Adamtsl1, Shc3, Hhat, Snhg14, Mroh2a, Rasa2, Zbtb20, Nell2, Pik3c2g, Dclk1, Col25a1, Septin9, Fras1, Rundc3b, Hnf4g, Trpm8, Dcdc5, Kcnh7, Arl15, L3mbtl4, Gm28494, Ttll7, Thsd4, Ptprn2, Ush2a, Prickle2, Cntnap5b, Mtus2, Trpm3, Dach1, Gm10649, Thrb, Kat6b, Edil3, Grm8, Sez6l, Unc13c, Pdzd2, Dpp10, Csgalnact1, Lama2, Kcnh1, Nol4, Mllt3, Ptprt, Tenm4, Mgat4c, Lrp1b, Cadm2, Cadps2, Cdh18, Dpp6, 2210408i21rik, 4921515e04rik, Prim2, Grid1, Slc4a4, Sgcz, Pdzrn4, Rgs7, Agbl1, Vav3, D130009i18rik, Pde10a, Kcnmb2, Opcml, Nav2, Fars2, Negr1, Gm15218, Dpyd, Pde1c, Erc2, Pde1a, Nova1, Mast4, Chrm3, Spag16, Grik2, Itpr2, Tmeff2, Plcb1, Rbfox2, Dnajc5b, Adamts3, Has2os, Dlgap1, Ssh2, Ablim1, Nr3c2, Dip2c, Rit2, Ift88, 9030622o22rik, Fbxl17, Tmem131l, Mipol1, Farp2, Bbx, Wwox, St6gal1, 9530036o11rik, Agap1, Brip1, Svep1, Tiam2, Mcf2l, Zfpm2, Stag1, Pcdh7, Gm34544, Gm11639, Gm13974, Abca5, Enox1, Uvrag, Snx25, Frmd4b, Nkain2, Prdm16, Creb5, Mctp1, Tns3, Dnah3, Rasgef1b, Lrba, Gng2, Akap13, Kcnip1, Lrch3, Usp29, Extl3, Lama3, Cog5, Pcdh11x, Htr4, Lhfpl3, Adgrv1, Stxbp5, Sipa1l1, Mgat5, Gria3, Zdhhc14, Snx29, Brwd1, Dlc1, Rims2, Eya4, Ctnna3, Unc80, Mctp2, Aco1, Agbl4, Mcc, Ptk2, Glis3, Grip1, Zfand3, Kcnq3.

---

**Number of elements in Set B: 237**

Abcc12, Abcg1, Acsl5, Adamtsl1, Adgrd1, Aff1, Aldh1a2, Ank3, Ankrd29, Ankrd34c-as1, Anks1b, Anxa3, Aptx, Arap2, Arhgef2, Arid1a, Arl5ap5, Asic2, Ass1p10, Atp2b2, Atxn1, Atxn3, B4galt6, Bmpr1b-dt, C17orf67, C1orf112, C22orf34, C4orf36, C9orf72, Calml3, Caln1, Camk1g, Camta1, Casc11, Ccdc192, Ccser1, Cdrt15p5, Cenpv, Cfap410, Chodl, Clcn3, Clvs1, Cnot2, Commd10, Cpne4, Creb1, Creb5, Ctdp1, Ctdsp1, Ctnnd2, Dach1, Disc1, Dnajb6p1, Dpp6, Efemp1, Egr1, Epb41, Erbb4, Ergic1, Erich6b, Etnppl, Fhdc1, Folh1b, G2e3, Grid1, Gse1, Hadh, Hla-dqb1, Hoxd10, Hspa9p1, Ide, Ifrd1, Inpp4b, Iqcf1, Iqcf5-as1, Itga9, Itpr2, Kalrn, Kc6, Kcng2, Kcnmb2, Kcnmb2-as1, Kcns3, Kdm4a, Kiaa0513, Kif5a, Kifap3, Klf6, Krt18p24, Krt18p3, Krt18p55, Lama2, Lama3, Laptm4a-dt, Lastr, Ldhc, Lef1, Linc00111, Linc00351, Linc00474, Linc00540, Linc01182, Linc01287, Linc01320, Linc01685, Linc01741, Linc01747, Linc01937, Linc02055, Linc02096, Linc02215, Linc02318, Linc02408, Linc02422, Linc02531, Linc02575, Linc02582, Linc02697, Linc02712, Linc02713, Linc02756, Lipc, Lipc-as1, Lrrc8c, Macrod2, Mapk1, Masp1, Meg8, Mettl21a, Mir1297, Mir99ahg, Mob3b, Mobp, Morn2, Mras, Mtco3p1, Mtnd5p1, Myom2, Nedd4l, Nfasc, Nfatc2, Nme9, Npap1p6, Npepps, Nrxn3, Nsfp1, Nt5c1a, Nudt4, Olfm4, Opcml, Or52k3p, Ostc, Pbdc1, Pcseat, Pcsk5, Pdgfrl, Pdlim5, Pdzph1p, Pigl, Plxna1, Pnpt1, Ppiap34,

Ppp2r2d, Ppy2p, Procr, Psd3, Pth2r, Ptprf, Ptprn2, Pvt1, Rbm19, Rbms1, Reep2, Rgs6, Rn7skp156, Rn7skp252, Rn7skp44, Rn7sl592p, Rnu1-98p, Rnu6-1069p, Rnu6-745p, Rnu7-29p, Rpl13ap25, Rpl23ap28, Rpl30p9, Rpl7p40, Rps10p27, Rps16p2, Rps18p12, Sarm1, Scfd1, Scn7a, Sec61gp1, Sell, Slc18a1, Slc25a12, Slc39a11, Slc9a8, Slc9a9, Smim2-as1, Sod1, Spp2, Sqle, St3gal3, St6galnac3, Stk36, Ston1, Ston1-gtf2a1l, Susd1, Susd2, Synpo2, Taf8, Tbc1d1, Tbk1, Tbxas1, Tcl6, Tfap2a, Tiam1, Tmem132b, Tnip1, Tpi1p1, Trpm8, Tspan9, Tubb4bp4, Tyw3, U3, Unc13a, Vil1, Wapl, Washc5, Wspar, Ypel1, Zbtb40, Zfp64, Zfyve26, Znf746, Znf767p.

-----------------------------------------------------------------------------------------------------------------------

**Number of elements in Set A∩B: 19**

Adamtsl1, Asic2, Camta1, Creb5, Ctnnd2, Dach1, Dpp6, Erbb4, Grid1, Itpr2, Kalrn, Kcnmb2, Lama2, Lama3, Macrod2, Mir99ahg, Opcml, Ptprn2, Trpm8.

-----------------------------------------------------------------------------------------------------------------------

**Number of elements in Set C: 608**

Depdc5, Dph6, Pcsk6, Fam13a, Shroom3, Itpr2, Adarb2, Cdh23, Dab1, Dcc, Slit3, Park2, Farp1, Iqcj, Irak3, Cadm2, Fam189a1, Kif13b, Grin2b, Aspa, App, Pxdnl, Malrd1, Fgf12, Anks1b, Luzp2, Atxn1, Hs3st4, Nrxn3, Tmem132d, Rbfox3, Cacna2d3, Ctif, Mdga2, Ptprd, Glis3, Galntl6, Macrod2, Sox5, Nxph1, Osbpl1a, Sez6l, Erbb4, Pcdh15, Zfpm2, Fam19a5, Znf652, Tnik, Dclk1, Adh7, Oprd1, Lrguk, Rnf14, Wdfy2, Pcnx, Ttc3, Iqgap1, Sun1, Trim44, Pald1, Myh11, Rbms2, Sgpp2, Erc1, Plgrkt, Blk, Sgms1, Nsmaf, Ergic1, Adrbk2, Akap7, Man2a1, Ttc29, Arap2, Scn8a, Cenpf, Stac, Anxa5, Ubr3, Celf4, Grm3, Srrm4, Apbb2, Atp2c2, Ddx31, Hcg26, Upf2, Zbtb38, Gpr176, Olfm3, Slc9a8, Piwil4, Dtd1, Cabin1, Col4a6, Gls2, Kcnmb1, Mid1, Tgoln2, Loc400958, Mir4500hg, Tbl1x, Tvp23c-cdrt4, Mkl2, Gucy2ep, Rnf17, Pigl, Frem3, Loc100507662, Kctd8, Neil3, Sell, Nptxr, Samd5, C2cd2, Oprm1, Map3k15, Shroom4, Neo1, Rtfdc1, Tango6, Capn9, Erich6b, Bbs9, Dhrs3, Kifap3, Adamts20, Chchd3, Lrpprc, Prlr, Dpf3, Capn13, Colec12, Tgif1, Abat, Asph, Zak, Cacna2d4, Kcnn3, Jph3, Sept9, Klhl29, Dtna, Kiaa1804, Pcsk5, Pkd1l1, Syne3, Habp2, Rapgef5, Lipc, Bre, Plekha7, Pacrg, Cntn6, Tacc2, Large, Plcb1, Grid2, Sdk1, Camk1d, Cpne4, Arhgef3, Kcnq1, Kcnma1, Agbl1, Csmd1, Rbfox1, Lrp1b, Spag16, Ccser1, Nav2, Grm7, Cdh4, Cdh13, Cntn5, Cntnap2, Hdac4, Unc13c, Dscam, Cdkal1, Ctnna3, Fhit, Robo2, Wwox, Nell1, Fam155a, Sgcz, Gpc6, Rora, Lingo2, Tenm2, Prkag2, Grm8, Cntn4, Dmd, Alk, Cdh12, Ssbp3, Ksr2, Nkain2, Fam19a1, Nalcn, Erg, Nrxn1, Ldlrad4, Creb5, Sntg2, Dock8, Abcc4, Hecw1, Sorcs2, Sorbs2, Acoxl, Grik2, Asic2, Gna14, Slc39a11, Agap1, Agbl4, Thsd7a, Sptlc3, Iqgap2, Pdzd2, Myo10, Snx29, Plxdc2, Tanc1, Ptprn2, Nckap5, Ptprt, Tiam1, Caln1, Znf804b, Pde4b, Wbscr17, Znf423, Kalrn, Fmn2, Mtus2, Fto, Opcml, Gmds, Trpm1, Prkca, Mast4, Naaladl2, Tp63, Ctnnd2, Zbtb20, Atxn7l1, Gabbr2, Mthfd1l, Prkcq, Myo1d, Sugct, Abca13, Myo18b, Znf385b, Gfra1, Fmn1, Creb3l2, Linc01121, Mgll, Nav3, Pla2g4e, Bank1, Galnt2, Cobll1, Fam149a, Mkln1, Slc24a2, Akap12, Slc6a5, Btbd16, Kdm4b, Adamts14, Slc2a13, Gpr116, Pax7, Gria3, Allc, Rbks, Gnat2, Plekhg6, Rtn4r, T, Il1a, Nrf1, Ptprf, Endod1, Kdm4a, Arhgap8, Fer, Lrrfip1, C9orf72, Ppp2r2c, Ccdc3, Lrig1, Tmem132c, Disc1, Rimbp2, Ntrk3, Col4a2, Osbp2, Ifi44l, Loc100128554, Fhdc1, Atp6v1e2, Unc13a, Unq6494, Parvb, Dpyd, Slc35f3, Fgf1, Mpzl1, Lama3, Ano1, Capza1, Npr3, Kcne1, Sytl3, Dcun1d2, Il1rapl2, Phc1, Tle2, Loc100129636, Galnt15, C1orf106, Zdhhc23, Atp2b3, Ppp1r12b, Stx17, Ak8, Acot9, Efhc2, Prdm16, Capg, Vsnl1, Igfn1, Gabra3, Glt8d2, Pcdhgb4, Nhsl1, Ccdc73, Iqce, Nek2, Arsg, Ltbp2, Kiaa0040, Dnm1l, Frmpd4, Zfp64, Col14a1, Diaph2, Ippk, Wdfy3, Slc25a26, Loc100506272, Palm2, Sgsm1, C3orf20, Eya1, Ppp1r14c, Il1rapl1, St3gal3, Grip1, Exo1, Vcan, Phf21a, Bnc2, Gli2, Shroom2, Spata16, Pdlim5, Grik4, Rap1gap2, Slc6a1, Cast, Mob3b, Btbd11, Insr, Pfkp, Loc100128714, C4orf19, Tmtc2, Loc285692, Cc2d2a, Tek, Zhx2, Col19a1, Abca9, Gxylt2, Elmsan1, Slco4a1, Enosf1, Linc00545, Pak4, Med16, Zdhhc13, Dchs1, Ift74, Adck4, Nkap, Nkrf, Alas2, Ankrd29, Znf414, Znf724p, Cxorf58, Fnbp1, Gdpd2, Znf271, Znf382, Zmat1, Trmt2b, Tmprss13, Tmem91,

Anks4b, Tmem200a, Tcea1, Syn1, Cenpv, Susd2, Stag2, Sstr5, Snrpd3, C21orf62, Glra2, Slc35c1, Sept6, Dhx35, Satl1, Rps6ka6, Rpl39, Robo3, Rnf126, Rnf122, Rab9a, Rab40b, Pla2g12b, Pcdhga11, Fam13b, Or8g5, Pcdh19, Nol4l, Or1e2, Or2j3, Col7a1, Morn2, Mis18a, Lurap1l, Magea11, Med17, Loc728392, Klhl15, Krt18p55, Lamp2, Klf8, Kiaa0513, Igsf1, Igbp1, Arhgef2, Bex5, Hdac8, Cdk13, Champ1, Znf839, Fndc3a, Frs2, Klhl4, Cd99l2, Prkx, Rgs9bp, Pof1b, Nampt, Rngtt, Rpf2, Tlr8, Awat2, Erich5, Adnp2, Apool, Clec2a, Nt5c1a, Stk36, Bcl6, Maob, Htr2a, Sytl4, Loc100652894, Upb1, Mapk1, Iqsec2, Mxra5, Zscan30, Stard8, Loc100652824, Rxra, C6orf183, Sarm1, Hiatl1, Csrp1, Arhgef37, Cep104, Magea10-magea5, Tubb, Orc5, Reps2, Cnot2, Synj2, Adora1, Trim27, C6orf58, Cep44, Nrk, Pasd1, Fbxo8, Crhbp, Rapgef2, Tenm1, Apoo, Trim2, Ptchd1, Tvp23c, C7orf57, Cd1a, Fmr1nb, Sun3, Tnmd, Ogt, Fam133a, Kcnmb3, Ptchd1-as, Gng7, Sh3kbp1, Nploc4, Cd1e, Ins-igf2, C2orf70, Ramp3, Ccl25, Prps2, Spam1, Myo1f, Kl, Spata22, Fgf13, Tiparp, Bard1, Ppp1r16b, Il36b, Jarid2, Thtpa, Arnt2, Evc2, Hmcn2, Susd1, Cdc42bpa, Col28a1, Csgalnact1, Nlgn4x, Ifrd1, Tlr1, Tlr10, Atp6v0d2, Mettl24, Fer1l6-as1, Vash2, Adcyap1r1, Daoa-as1, Tulp4, Gucd1, Znf185, Nub1, Hla-doa, Wwp2, Pbx1, Rapgef4, Tnpo3, Loc100506023, Tmtc1, Aven, Susd3, Lasp1, Mgam, Fam126a, Adcy2, Rgs6, Kcnip4, Col27a1, Rab3c, Fhod3, Srgap3, Acyp2, Krt4, Atp1a4, Gas6, Kdm5a, Gpc4, Slc38a1, Eda, Fam19a4, Fam49a, Plxna2, Sh3tc2, Prex1, Hnf1b, Stap1.

---

**Number of elements in Set A∩C: 54**

Adcy2, Agap1, Agbl1, Agbl4, Asic2, Cadm2, Cdh13, Cdh4, Cntn5, Creb5, Csgalnact1, Csmd1, Ctnna3, Ctnnd2, Dclk1, Dpyd, Erbb4, Fam189a1, Galntl6, Glis3, Gria3, Grik2, Grip1, Grm7, Grm8, Il1rapl2, Itpr2, Kalrn, Lama3, Lrp1b, Macrod2, Mast4, Mtus2, Nav2, Nckap5, Nkain2, Opcml, Pde4b, Pdzd2, Plcb1, Prdm16, Ptprd, Ptprn2, Ptprt, Rbfox1, Sez6l, Sgcz, Snx29, Sox5, Spag16, Unc13c, Wwox, Zbtb20, Zfpm2.

---

**Number of elements in Set A∩D: 42**

Arl15, Cadm2, Cadps, Cdh13, Cdh4, Ctnna3, Dclk1, Dlg2, Dpp10, Dpp6, Edil3, Ehbp1, Epb41l3, Erc2, Gng2, Gria3, Grm7, Grm8, Itpr2, Lama2, Lama3, Macrod2, Magi1, Mipol1, Negr1, Nova1, Ntm, Pcca, Pde1c, Plcb1, Prdm16, Ptprn2, Rbfox1, Sipa1l1, Slc4a4, Slc7a11, Stxbp5, Tbc1d5, Tenm4, Tln2, Wwox, Zbtb20.

---

**Number of elements in Set A∩B∩C: 10**

Asic2, Creb5, Ctnnd2, Erbb4, Itpr2, Kalrn, Lama3, Macrod2, Opcml, Ptprn2.

---

**Number of elements in Set A∩B∩D: 6**

Dpp6, Itpr2, Lama2, Lama3, Macrod2, Ptprn2.

---

**Number of elements in Set A∩C∩D: 17**

Cadm2, Cdh13, Cdh4, Ctnna3, Dclk1, Gria3, Grm7, Grm8, Itpr2, Lama3, Macrod2, Plcb1, Prdm16, Ptprn2, Rbfox1, Wwox, Zbtb20.

---

**Number of elements in Set A∩B∩C∩D: 4**

Itpr2, Lama3, Macrod2, Ptprn2.

---

**Number of elements in Set A-A∩B-A∩C-A∩D+A∩B∩C+A∩B∩D+A∩C∩D-A∩B∩C∩D: 139**

2210408i21rik, 4921515e04rik, 9030622o22rik, 9530036o11rik, Abca5, Abcg2, Ablim1, Aco1, Adamts2, Adamts3, Adgrl3, Adgrv1, Aff3, Akap13, Aw554918, Bbx, Brip1, Brwd1, Cacnb2, Cadps2, Car10, Cdh18, Chrm3, Cntnap5b, Cog5, Col25a1, Csmd3, Cx3cr1, D130009i18rik,

D430041d05rik, Dcdc5, Dgki, Dip2c, Dlc1, Dlgap1, Dlgap2, Dnah3, Dnajc5b, Egfem1, Enox1, Extl3, Eya4, Farp2, Fars2, Fbxl17, Fbxl7, Foxn3, Foxp2, Fras1, Frmd4b, Fstl4, Galnt2l, Gm10649, Gm11639, Gm12648, Gm13974, Gm15218, Gm28494, Gm34544, Gm37240, Has2os, Hhat, Hnf4g, Hs6st3, Htr4, Ift88, Kat6b, Kcnh1, Kcnh7, Kcnip1, Kcnq3, L3mbtl4, Large1, Lhfpl3, Lrba, Lrch3, Lrrc4c, Mcc, Mcf2l, Mctp1, Mctp2, Mgat4c, Mgat5, Mllt3, Mroh2a, Nell2, Nol4, Nr3c2, Nrg3, Pcdh11x, Pcdh7, Pde10a, Pde1a, Pdzrn4, Phactr1, Pik3c2g, Prickle2, Prim2, Ptk2, Rasa2, Rasgef1b, Rbfox2, Rbms3, Rgs7, Rims2, Rit2, Rundc3b, Ryr2, Sema3d, Septin9, Shc3, Snd1, Snhg14, Sntg1, Snx25, Ssh2, St6gal1, Stag1, Svep1, Thrb, Thsd4, Thsd7b, Tiam2, Tmc1, Tmeff2, Tmem131l, Tns3, Trpm3, Ttc28, Ttll7, Unc80, Ush2a, Usp29, Uvrag, Vav3, Vti1a, Zdhhc14, Zfand3, Zfp407.

---

**Number of elements in Set A∩B-A∩B∩C-A∩B∩D+A∩B∩C∩D: 7**

Adamtsl1, Camta1, Dach1, Grid1, Kcnmb2, Mir99ahg, Trpm8.

---

**Number of elements in Set A∩B∩C-A∩B∩C∩D: 6**

Asic2, Creb5, Ctnnd2, Erbb4, Kalrn, Opcml.

---

**Number of elements in Set A∩C-A∩B∩C-A∩C∩D+A∩B∩C∩D: 31**

Adcy2, Agap1, Agbl1, Agbl4, Cntn5, Csgalnact1, Csmd1, Dpyd, Fam189a1, Galntl6, Glis3, Grik2, Grip1, Il1rapl2, Lrp1b, Mast4, Mtus2, Nav2, Nckap5, Nkain2, Pde4b, Pdzd2, Ptprd, Ptprt, Sez6l, Sgcz, Snx29, Sox5, Spag16, Unc13c, Zfpm2.

---

**Number of elements in Set A∩C∩D-A∩B∩C∩D: 13**

Cadm2, Cdh13, Cdh4, Ctnna3, Dclk1, Gria3, Grm7, Grm8, Plcb1, Prdm16, Rbfox1, Wwox, Zbtb20.

---

**Number of elements in Set A∩B∩D-A∩B∩C∩D: 2**

Dpp6, Lama2.

---

**Number of elements in Set A∩D-A∩B∩D-A∩C∩D+A∩B∩C∩D: 23**

Arl15, Cadps, Dlg2, Dpp10, Edil3, Ehbp1, Epb41l3, Erc2, Gng2, Magi1, Mipol1, Negr1, Nova1, Ntm, Pcca, Pde1c, Sipa1l1, Slc4a4, Slc7a11, Stxbp5, Tbc1d5, Tenm4, Tln2.

---

**Number of elements in Set B∩C: 52**

Ankrd29, Anks1b, Arap2, Arhgef2, Asic2, Atxn1, C9orf72, Caln1, Ccser1, Cenpv, Cnot2, Cpne4, Creb5, Ctnnd2, Disc1, Erbb4, Ergic1, Erich6b, Fhdc1, Ifrd1, Itpr2, Kalrn, Kdm4a, Kiaa0513, Kifap3, Krt18p55, Lama3, Lipc, Macrod2, Mapk1, Mob3b, Morn2, Nrxn3, Nt5c1a, Opcml, Pcsk5, Pdlim5, Pigl, Ptprf, Ptprn2, Rgs6, Sarm1, Sell, Slc39a11, Slc9a8, St3gal3, Stk36, Susd1, Susd2, Tiam1, Unc13a, Zfp64.

---

**Number of elements in Set B∩D: 46**

Acsl5, Ank3, Anxa3, Arid1a, Atp2b2, Atxn3, Cenpv, Clvs1, Cnot2, Dpp6, Epb41, Ergic1, Etnppl, Hadh, Ide, Itpr2, Kiaa0513, Kif5a, Kifap3, Lama2, Lama3, Macrod2, Mobp, Mras, Nedd4l, Nfasc, Npepps, Nrxn3, Ostc, Pbdc1, Pdlim5, Psd3, Ptprf, Ptprn2, Reep2, Rgs6, Sarm1, Scfd1, Slc25a12, Sod1, Susd2, Synpo2, Tbc1d1, Tiam1, Unc13a, Wapl.

---

**Number of elements in Set B∩C∩D: 17**

Cenpv, Cnot2, Ergic1, Itpr2, Kiaa0513, Kifap3, Lama3, Macrod2, Nrxn3, Pdlim5, Ptprf, Ptprn2, Rgs6, Sarm1, Susd2, Tiam1, Unc13a.

---

**Number of elements in Set B-A∩B-B∩C-B∩D+A∩B∩C+A∩B∩D+B∩C∩D-A∩B∩C∩D: 149**

Abcc12, Abcg1, Adgrd1, Aff1, Aldh1a2, Ankrd34c-as1, Aptx, Arl5ap5, Ass1p10, B4galt6, Bmpr1b-dt, C17orf67, C1orf112, C22orf34, C4orf36, Calml3, Camk1g, Casc11, Ccdc192, Cdrt15p5, Cfap410, Chodl, Clcn3, Commd10, Creb1, Ctdp1, Ctdsp1, Dnajb6p1, Efemp1, Egr1, Folh1b, G2e3, Gse1, Hla-dqb1, Hoxd10, Hspa9p1, Inpp4b, Iqcf1, Iqcf5-as1, Itga9, Kc6, Kcng2, Kcnmb2-as1, Kcns3, Klf6, Krt18p24, Krt18p3, Laptm4a-dt, Lastr, Ldhc, Lef1, Linc00111, Linc00351, Linc00474, Linc00540, Linc01182, Linc01287, Linc01320, Linc01685, Linc01741, Linc01747, Linc01937, Linc02055, Linc02096, Linc02215, Linc02318, Linc02408, Linc02422, Linc02531, Linc02575, Linc02582, Linc02697, Linc02712, Linc02713, Linc02756, Lipc-as1, Lrrc8c, Masp1, Meg8, Mettl21a, Mir1297, Mtco3p1, Mtnd5p1, Myom2, Nfatc2, Nme9, Npap1p6, Nsfp1, Nudt4, Olfm4, Or52k3p, Pcseat, Pdgfrl, Pdzph1p, Plxna1, Pnpt1, Ppiap34, Ppp2r2d, Ppy2p, Procr, Pth2r, Pvt1, Rbm19, Rbms1, Rn7skp156, Rn7skp252, Rn7skp44, Rn7sl592p, Rnu1-98p, Rnu6-1069p, Rnu6-745p, Rnu7-29p, Rpl13ap25, Rpl23ap28, Rpl30p9, Rpl7p40, Rps10p27, Rps16p2, Rps18p12, Scn7a, Sec61gp1, Slc18a1, Slc9a9, Smim2-as1, Spp2, Sqle, St6galnac3, Ston1, Ston1-gtf2a1l, Taf8, Tbk1, Tbxas1, Tcl6, Tfap2a, Tmem132b, Tnip1, Tpi1p1, Tspan9, Tubb4bp4, Tyw3, U3, Vil1, Washc5, Wspar, Ypel1, Zbtb40, Zfyve26, Znf746, Znf767p.

---

**Number of elements in Set B∩C-A∩B∩C-B∩C∩D+A∩B∩C∩D: 29**

Ankrd29, Anks1b, Arap2, Arhgef2, Atxn1, C9orf72, Caln1, Ccser1, Cpne4, Disc1, Erich6b, Fhdc1, Ifrd1, Kdm4a, Krt18p55, Lipc, Mapk1, Mob3b, Morn2, Nt5c1a, Pcsk5, Pigl, Sell, Slc39a11, Slc9a8, St3gal3, Stk36, Susd1, Zfp64.

---

**Number of elements in Set B∩C∩D-A∩B∩C∩D: 13**

Cenpv, Cnot2, Ergic1, Kiaa0513, Kifap3, Nrxn3, Pdlim5, Ptprf, Rgs6, Sarm1, Susd2, Tiam1, Unc13a.

---

**Number of elements in Set B∩D-A∩B∩D-B∩C∩D+A∩B∩C∩D: 27**

Acsl5, Ank3, Anxa3, Arid1a, Atp2b2, Atxn3, Clvs1, Epb41, Etnppl, Hadh, Ide, Kif5a, Mobp, Mras, Nedd4l, Nfasc, Npepps, Ostc, Pbdc1, Psd3, Reep2, Scfd1, Slc25a12, Sod1, Synpo2, Tbc1d1, Wapl.

---

**Number of elements in Set C∩D: 131**

Acot9, Acyp2, Akap12, Akap7, Anxa5, Apbb2, App, Arsg, Aspa, Asph, Atp1a4, Aven, Btbd11, Cacna2d3, Cadm2, Capg, Capza1, Cast, Cdc42bpa, Cdh13, Cdh4, Cenpv, Cep104, Chchd3, Cnot2, Cntnap2, Cobll1, Csrp1, Ctif, Ctnna3, Dab1, Dclk1, Dcun1d2, Dnm1l, Dtna, Endod1, Erc1, Ergic1, Fam49a, Farp1, Fgf1, Fgf12, Fto, Gabbr2, Gabra3, Gng7, Gpc4, Gria3, Grm3, Grm7, Grm8, Hecw1, Igbp1, Iqgap1, Iqsec2, Itpr2, Kctd8, Kiaa0513, Kifap3, Lama3, Lamp2, Lrpprc, Macrod2, Mgll, Myh11, Nampt, Nploc4, Nrxn1, Nrxn3, Ogt, Olfm3, Orc5, Osbpl1a, Pald1, Palm2, Parvb, Pdlim5, Pfkp, Plcb1, Plgrkt, Plxdc2, Ppp1r16b, Ppp2r2c, Prdm16, Prex1, Prkag2, Prkca, Ptprf, Ptprn2, Rab3c, Rap1gap2, Rapgef2, Rapgef4, Rbfox1, Rbms2, Reps2, Rgs6, Rimbp2, Rnf126, Rnf14, Rpf2, Rpl39, Sarm1, Scn8a, Sept6, Sept9, Sgsm1, Sh3kbp1, Shroom2, Slc6a1, Snrpd3, Sorbs2, Sorcs2, Srgap3, Susd2, Syn1, Syne3, Synj2, Sytl4, Tbl1x, Tcea1, Thsd7a, Tiam1, Tnpo3, Ubr3, Unc13a, Vcan, Vsnl1, Wwox, Zbtb20, Zhx2.
